# Supplementary material for: Auxin‐dependent regulation of cell division rates governs root thermomorphogenesis
Source: EMBO J. 2023 Apr 18;42(11):e111926. doi: 10.15252/embj.2022111926 (PMC10233379; doi:10.15252/embj.2022111926)
Supplement: Supplementary file 1 — Expanded View Figures PDF [file EMBJ-42-e111926-s004.pdf]

Expanded View Figures

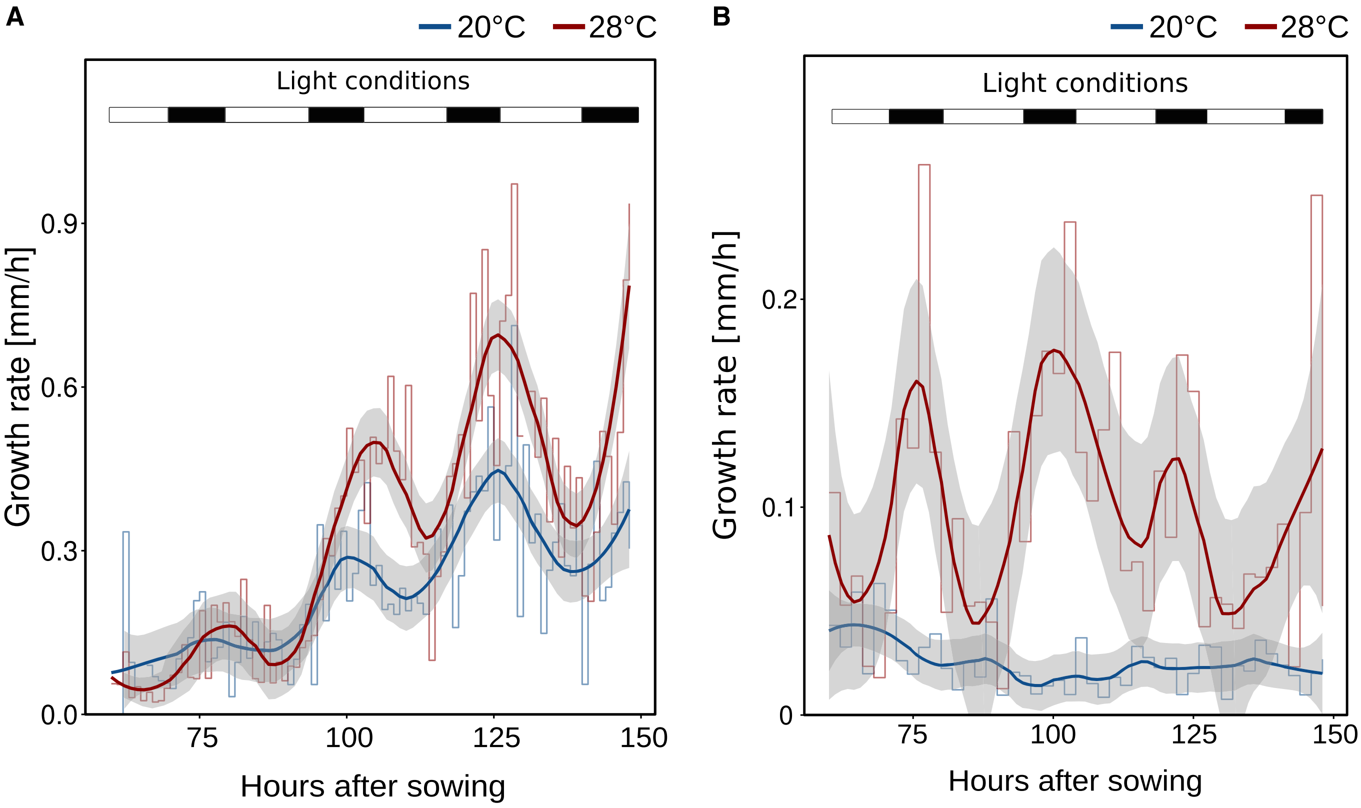

Figure EV1. Growth dynamics in response to different temperatures.

A, B Growth rates of seedling (A) roots and (B) hypocotyls between days 2 and 7 were assessed by hourly and 2-hourly infra-red real-time imaging, respectively. Mean growth rates are shown as step-wise lines (n = 7–8) that were fitted with a "loess" smoothing function shown as solid lines and the respective 95% confidence intervals are shown as gray ribbons. Long-day lighting is schematically depicted by white (light) and black (dark) bars on the top.

Source data are available online for this figure.

 © 2023 The Authors

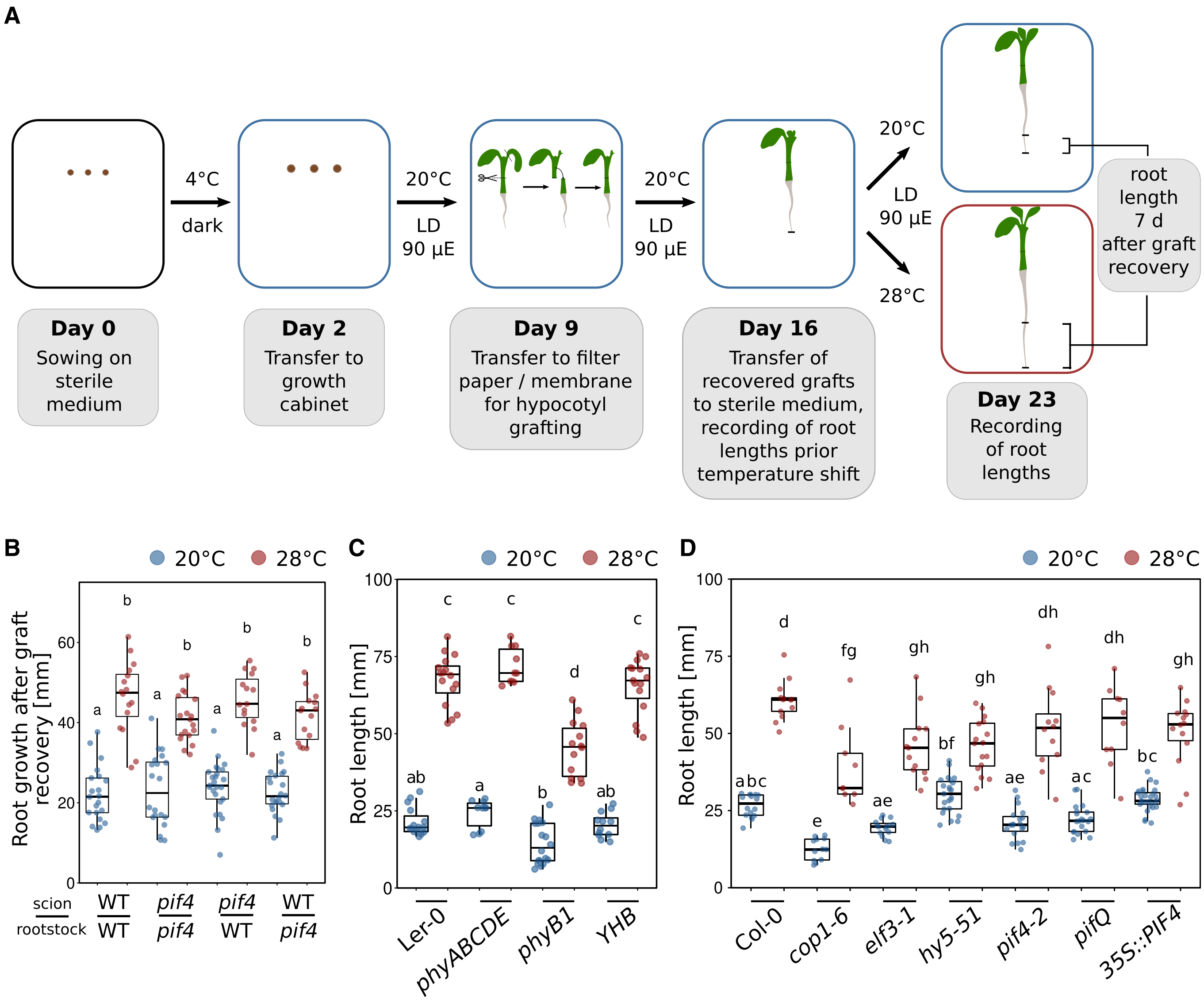

Figure EV2. Micrografting assays and root temperature responses in selected mutants.

A, B (A) Schematic representation of the grafting experiments shown in (B) and Fig 2B–D. Temperature-induced root elongation of (B) *pif4-2* mutants grafted with their corresponding wild-type Col-0 (n = 15–25).

C, D Root growth assay of seedlings grown for 7 days (C: n = 10–18, D: n = 9–29) at the indicated temperatures. The *phyB* Y276H mutant (YHB; Su & Lagarias, 2007) in (C) adopts a light-insensitive, physiologically active conformation and can be described as constitutively active. (B–D) Boxplots show medians, interquartile ranges, and min–max values with individual data points superimposed as colored dots. Different letters denote statistical differences at $P < 0.05$ as assessed by two-way ANOVA and Tukey's HSD *post hoc* test.

Source data are available online for this figure.

© 2023 The Authors

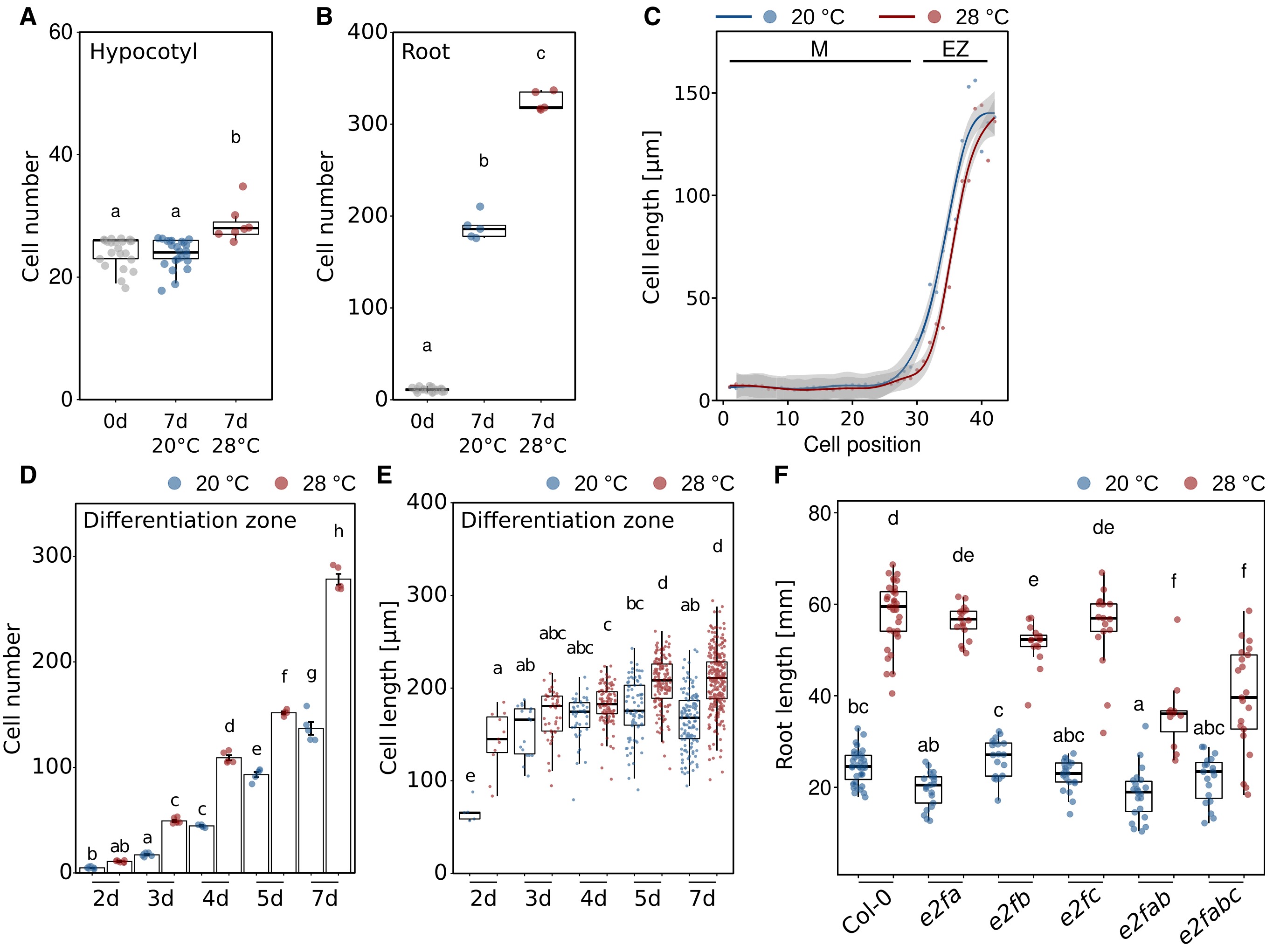

Figure EV3. Changes in root and shoot cell numbers from mature embryos to 7-day-old seedlings.

A, B Number of cells in a consecutive cell file of (A) hypocotyls and (B) roots of mature embryos prior to germination (0 days) and in 7-day-old seedlings grown at 20 or 28°C.

C Close-up view of the first 43 cells comprising meristem (M) and elongation zone (EZ) of data shown in Fig 3D.

D Total number of cells in a longitudinal cortex cell file of the differentiation zone between days 2 and 7 after sowing. Barplots show mean values, error bars indicate SEM. Individual data points are plotted as colored dots (n = 5–7).

E Mean lengths of all cells in a consecutive cell file of the differentiation zone measured in 5–7 individual roots. Number of cells ranges from n = 5 (2 days, 20°C) to n = 272 (7 days, 28°C).

F Temperature-induced root elongation in 7-day-old wild-type, single-, and higher-order *e2f* mutants (n = 11–39). Boxplots show medians, interquartile ranges, and min–max values with individual data points superimposed as colored dots. Different letters denote statistical differences at $P < 0.05$ as assessed by one-way ANOVA (A, B) or two-way ANOVA (D–F) and Tukey's HSD *post hoc* test.

Source data are available online for this figure.

 © 2023 The Authors

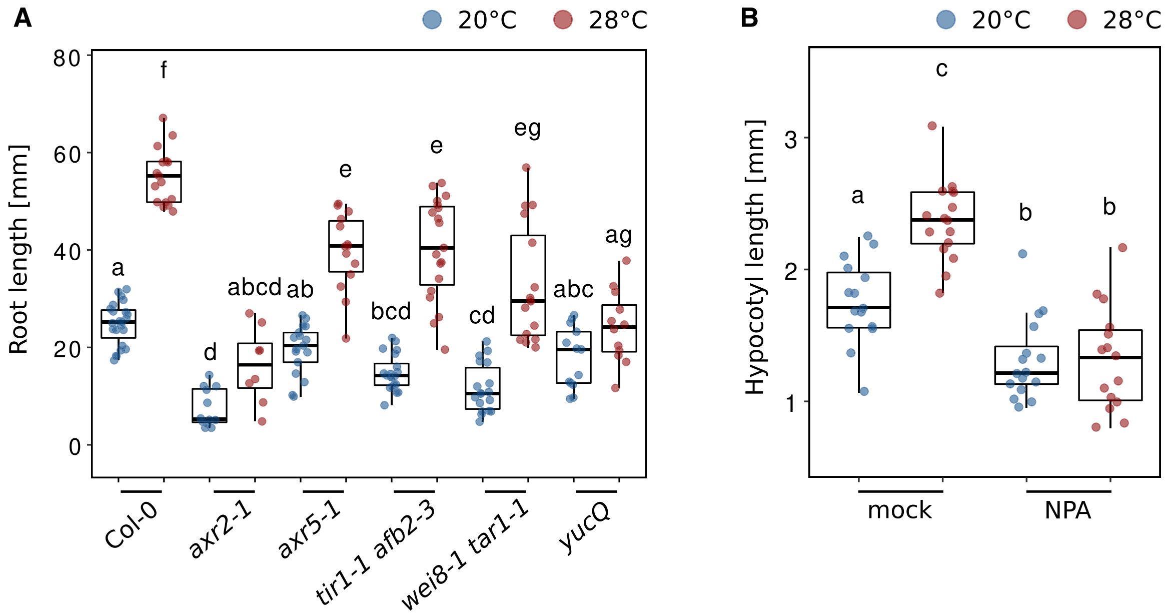

Figure EV4. Temperature-induced root elongation in auxin biosynthesis and signaling mutants.

A Root length of 7-day-old Arabidopsis seedlings grown in LD at 20 or 28°C (n = 8–23).

B Temperature-induced hypocotyl elongation in 8-day-old seedlings with mock or 0.5 mM NPA applied to the cotyledons (n = 15–16). (A, B) Boxplot shows medians, interquartile ranges, and min–max values with individual data points superimposed as colored dots. Different letters denote statistical differences at $P < 0.05$ as assessed by two-way ANOVA and Tukey's HSD *post hoc* test.

Source data are available online for this figure.

© 2023 The Authors

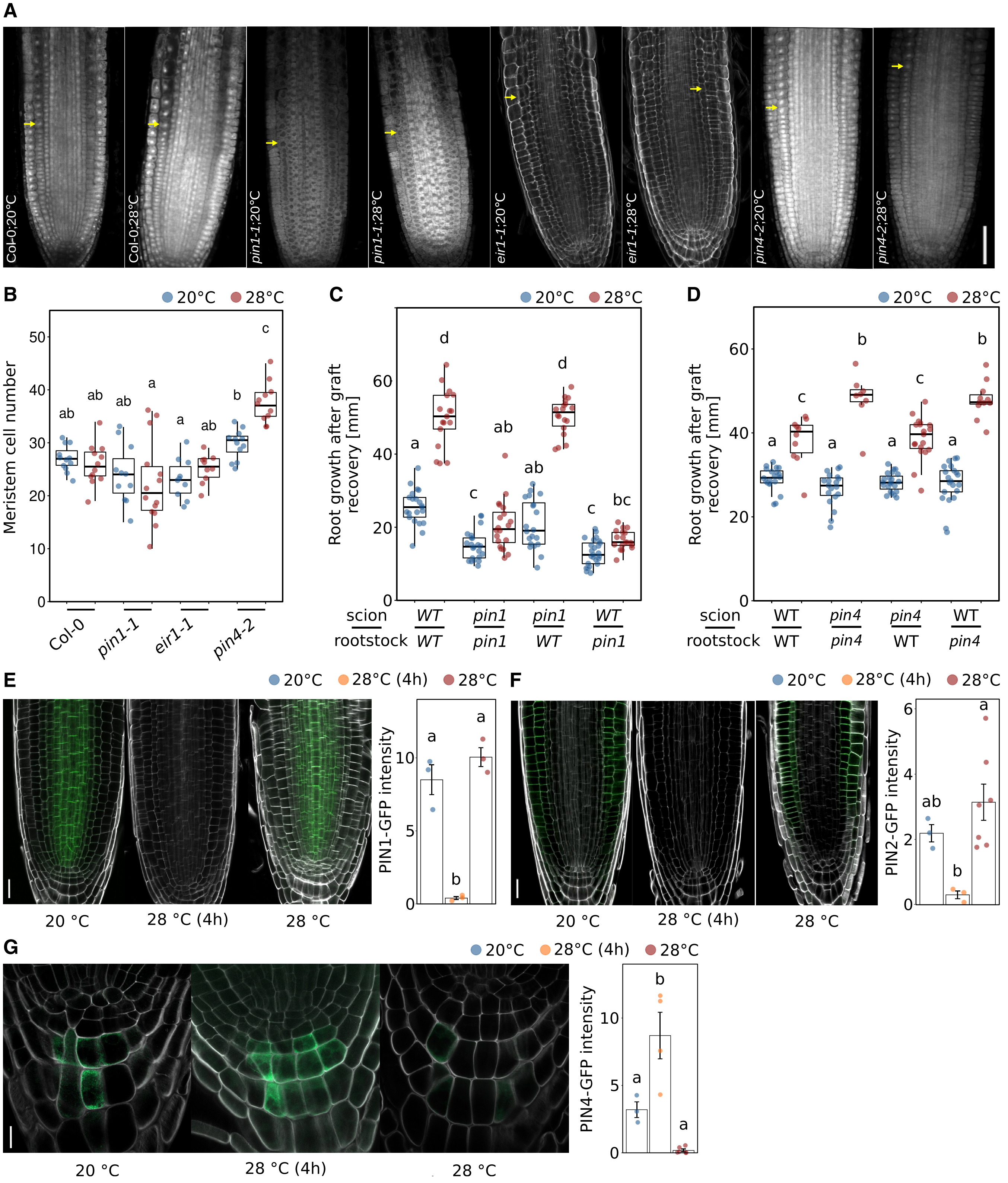

Figure EV5.

 © 2023 The Authors

Figure EV5. PIN1 and PIN2 positively regulate root thermomorphogenesis.

A Microscopic photographs of root tips of 5-day-old seedlings grown in LD at 20 or 28°C. Yellow arrows mark the end of the meristem. Scale bar = 50 μm.

B Quantification of meristem cell numbers in consecutive cortex cell files. Root growth assay of 7-day-old seedlings (n = 13–30) grown at the indicated temperatures.

C, D Nine-day-old seedlings grown at 20°C were hypocotyl grafted, recovered for 7 days, and then cultivated at 20 or 28°C for another 7 days (C: n = 17–25, D: n = 8–23).

E–G Temperature effects on PIN1-GFP (E, scale bar = 20 μm), PIN2-GFP (F, scale bar = 20 μm), and PIN4-GFP (G, scale bar = 10 μm) levels. Seedlings were grown for 5 days at constant temperatures (20 or 28°C) or grown at 20°C for 5 days and shifted to 28°C for 4 h (n = 3–7). (B–G) Boxplots show medians, interquartile ranges, and min–max values (B–D). Barplots show mean values and error bars show SEM (E–G). Individual data points superimposed as colored dots. Different letters denote statistical differences at $P < 0.05$ as assessed by one-way (E–G) or two-way (B–D) ANOVA and Tukey's HSD *post hoc* test.

Source data are available online for this figure.

© 2023 The Authors
