# Supplementary material for: Auxin‐dependent regulation of cell division rates governs root thermomorphogenesis
Source: EMBO J. 2023 Apr 18;42(11):e111926. doi: 10.15252/embj.2022111926 (PMC10233379; doi:10.15252/embj.2022111926)
Supplement: Supplementary file 2 — Source Data for Expanded View [file EMBJ-42-e111926-s001.zip › FigureEV1/FigureEV1_README.rtf]

FigureEV1A-B: Growth rates of roots and hypocotylsSeeds were surface sterilized, rinsed with sterile water, and then imbibed and stratified for 3 days at 4°C in deionized water before sowing. Vertically oriented ATS plates (with 2 days-old seedlings grown at 20°C or 28°C) were put perpendicular to the camera (Panasonic G5 with hotmirror filter replaced by an IR filter, enabling only IR light to reach the sensor; www.irrecams.de). To monitor root growth dynamics, pictures were automatically taken every hour (roots) or every two hours (hypocotyls). Root and hypocotyl length were measured in RootDetection (www.labutils) and lengths were used to calculate growth rates.
