# Supplementary material for: Auxin‐dependent regulation of cell division rates governs root thermomorphogenesis
Source: EMBO J. 2023 Apr 18;42(11):e111926. doi: 10.15252/embj.2022111926 (PMC10233379; doi:10.15252/embj.2022111926)
Supplement: Supplementary file 2 — Source Data for Expanded View [file EMBJ-42-e111926-s001.zip › FigureEV2/FigureEV2_README.rtf]

FigureEV2B: Root length of pif4 grafted seedlingsSeeds were sown on ATS medium at 4°C darkness, stratified for 2 days at 4°C and then shifted to 20°C in a growth cabinet for another 7 days under long-day photoperiods (16 h of light/8 h of dark) with 90 µmol m− s− white light (T5 4000K). Next, seedlings were grafted and recovered for 7 days on a water mounted filter paper/membrane. Successfully recovered grafted plants were selected, transferred to new ATS medium and cultivated at 20°C or 28°C, respectively, under the same conditions described above for another 7 days. Root growth after graft recovery was then determined by measuring the root growth difference between day 16 and day 23 and given in mm. FigureEV2C-D: Root length in selected mutants with altered shoot temperature responsesSurface sterilized seeds were rinsed with sterile water and then imbibed and stratified for 3 days at 4°C in deionized water before sowing on solid Arabidopsis thaliana solution (ATS, Lincoln et al., 1990) nutrient medium including 1 % (w/v) sucrose on vertically oriented plates under long-day conditions (16 h of light/8 h of dark) with 90 µmol m− s− photosynthetically active radiation (PAR) from white fluorescent lamps (T5 4000K) at 20 or 28°C. Measurements were based on digital photographs of plates after 7 days of cultivation using RootDetection (www.labutils.de) and depict the total length of the root in mm. 
