# Supplementary material for: Auxin‐dependent regulation of cell division rates governs root thermomorphogenesis
Source: EMBO J. 2023 Apr 18;42(11):e111926. doi: 10.15252/embj.2022111926 (PMC10233379; doi:10.15252/embj.2022111926)
Supplement: Supplementary file 2 — Source Data for Expanded View [file EMBJ-42-e111926-s001.zip › FigureEV3/FigureEV3_README.rtf]

FigureEV3Cell measurements and counts were conducted by staining seedlings with Calcofluor White (Merck, 18909-100ML-F). Briefly, seedlings were fixed in pure ethanol for 2 h to overnight, washed twice with 1x PBS, followed by a permeabilization step using 3 % Triton X-100 + 10 % DMSO in 1x PBS for 30 min to 1 h. Next, 0.1 % Calcofluor White in 1x PBS was freshly prepared and the seedlings were stained for 30 min. Subsequently, seedlings were washed twice in 1x PBS with gentle shaking. To image Calcofluor White, we used 405 nm excitation and detected signals at 425 - 475 nm. All measurements were performed on all individual cells of a consecutive cortex cell file using the ZEN 3.1 software (Zeiss) for 8 - 12 independent seedlings per experiment. The meristematic zone was defined as the zone between the quiescent centre and the last cell that did not yet double its size in comparison with the previous cell. The elongation zone followed the meristematic zone and was defined as the zone from first cell with double the size of the previous cell to the last cell before root hair bulges became visible. The following differentiation and maturation zone was defined as the zone from first cell below the first trichoblast bulge to the root-shoot junction.FigureEV3C: Cell length in meristem and elongation zone of the rootThis is a subset of the data shown in Figure3D (Cell position 1-43). Raw data can be found in the respective file.FigureEV3F:Surface-sterilized seeds were placed on ATS medium after stratification for 3 days at 4°C. Seedlings were grown at 20°C or 28°C and root length was determined 7 days. All measurements were based on digital photographs of plates using RootDetection (www.labutils.de) and depict the total length of the root in mm. 
