# Supplementary material for: Auxin‐dependent regulation of cell division rates governs root thermomorphogenesis
Source: EMBO J. 2023 Apr 18;42(11):e111926. doi: 10.15252/embj.2022111926 (PMC10233379; doi:10.15252/embj.2022111926)
Supplement: Supplementary file 2 — Source Data for Expanded View [file EMBJ-42-e111926-s001.zip › FigureEV4/FigureEV4_README.rtf]

FigureEV4A:Surface-sterilized seeds were placed on ATS medium after stratification for 3 days at 4°C. Seedlings were grown at 20°C or 28°C and root length was determined 7 days. All measurements were based on digital photographs of plates using RootDetection (www.labutils.de) and depict the total length of the root in mm. FigureEV4B:Surface-sterilized seeds were placed on ATS medium after stratification for 3 days at 4°C. seedlings were pre-grown at 20°C for 5 days and then NPA strips were applied for an additional 3 days at either 20°C or 28°C, respectively. For NPA strips thin tissue strips were soaked in lukewarm ATS medium with or without the addition of 0.5 mM NPA (Duchefa). Strips were carefully placed across petioles/cotyledons or the root-shoot junction. Hypocotyl length were measured using RootDetection (labutils) based on digital photographs and length are given in mm.
