# Supplementary material for: Auxin‐dependent regulation of cell division rates governs root thermomorphogenesis
Source: EMBO J. 2023 Apr 18;42(11):e111926. doi: 10.15252/embj.2022111926 (PMC10233379; doi:10.15252/embj.2022111926)
Supplement: Supplementary file 2 — Source Data for Expanded View [file EMBJ-42-e111926-s001.zip › FigureEV5/FigureEV5_README.rtf]

FigureEV5A: Meristem size in pin mutantsSurface-sterilized seeds were placed on ATS medium after stratification for 3 days at 4°C. Seedlings were grown at 20°C or 28°C for 5 days. Seedlings were fixed in pure ethanol for 2 hrs to overnight, washed twice with 1x PBS, followed by a permeabilization step using 3 % Triton X-100 + 10 % DMSO in 1x PBS for 30 min to 1 h. Next, 0.1 mg ml− propidium iodide in 1x PBS was freshly prepared and the seedlings were stained for 3 min. Subsequently, seedlings were washed twice in 1x PBS with gentle shaking. To image Propidium iodide, we used 488nm excitation and detected signals at 600 - 637nm.FigureEV5B: Meristem cell number in pin mutantsSurface-sterilized seeds were placed on ATS medium after stratification for 3 days at 4°C. Seedlings were grown at 20°C or 28°C and root length was determined 7 days. All measurements were based on digital photographs of plates using RootDetection (www.labutils.de) and depict the total length of the root in mm. FigureEV5C-D: Root length of grafted pin mutantsSeeds were sown on ATS medium at 4°C darkness, stratified for 2 days at 4°C and then shifted to 20°C in a growth cabinet for another 7 days under long-day photoperiods (16 h of light/8 h of dark) with 90 µmol m− s− white light (T5 4000K). Next, seedlings were grafted and recovered for 7 days on a water mounted filter paper/membrane. Successfully recovered grafted plants were selected, transferred to new ATS medium and cultivated at 20°C or 28°C, respectively, under the same conditions described above for another 7 days. Root growth after graft recovery was then determined by measuring the root growth difference between day 16 and day 23 and given in mm. FigureEV5E-G: Confocal microscopy of PIN-GFP reportersPlants were stratified at 4°C for 2 days, grown on standard ½ MS media containing 1 % sucrose in a 16/8 hrs light/dark cycle at 20°C or 28°C for 5 days. For short term 20°C to 28°C shift experiments, plants grown at 20°C were incubated for an additional 4 h at 28°C. All plants were fixed and cleared using a previously established Clearsee-based protocol (Kurihara et al., 2015) modified to include Calcofluor White for staining of cell walls (Ursache et al., 2018). Briefly, plants were fixed in 4 % paraformaldehyde in 1x PBS for 1 h followed by three brief washes in 1x PBS and incubated overnight in Clearsee solution containing Calcofluor White. Images were acquired on a Zeiss LSM-980 confocal system equipped with an Airyscan 2 detector using either a 40x (1.0 NA) water immersion objective or a 63x (1.4 NA) oil immersion for Airyscan images. Calcofluor White signal was detected using a 405 nm laser for excitation and an emission window from 420 - 430 nm. For GFP, excitation was achieved using a 488 nm laser and the emission window was 500 - 525nm. For RFP, excitation was achieved at 561 nm and emission collected at 580 - 620 nm. All images were acquired as non-saturated 16-bit sequential scans for further quantification.
