# Supplementary figures and images for: Auxin‐dependent regulation of cell division rates governs root thermomorphogenesis
Source: EMBO J. 2023 Apr 18;42(11):e111926. doi: 10.15252/embj.2022111926 (PMC10233379; doi:10.15252/embj.2022111926)

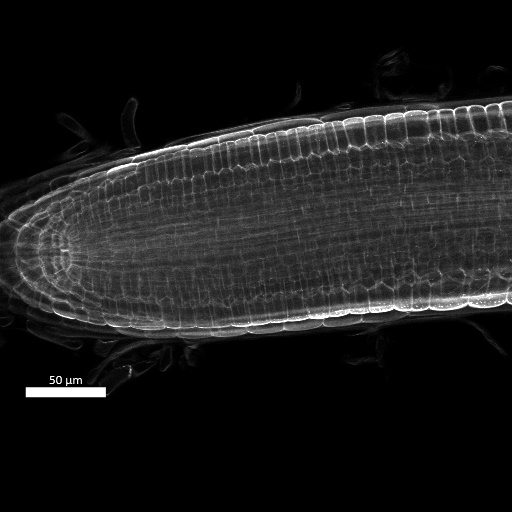

Supplement: Supplementary file 2 — Source Data for Expanded View [file EMBJ-42-e111926-s001.zip › FigureEV5/EV5A/Col-0_20c.jpg]

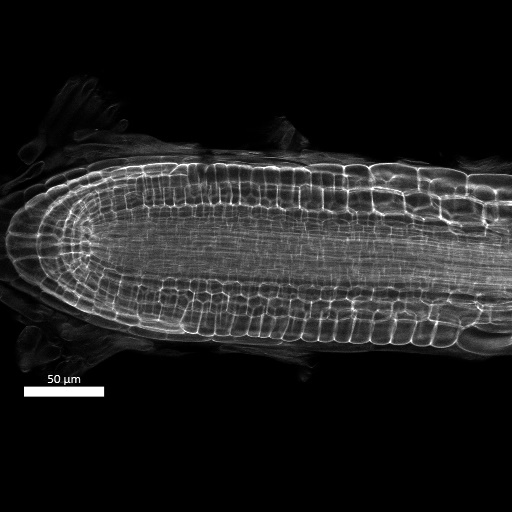

Supplement: Supplementary file 2 — Source Data for Expanded View [file EMBJ-42-e111926-s001.zip › FigureEV5/EV5A/pin1-1_28c.jpg]

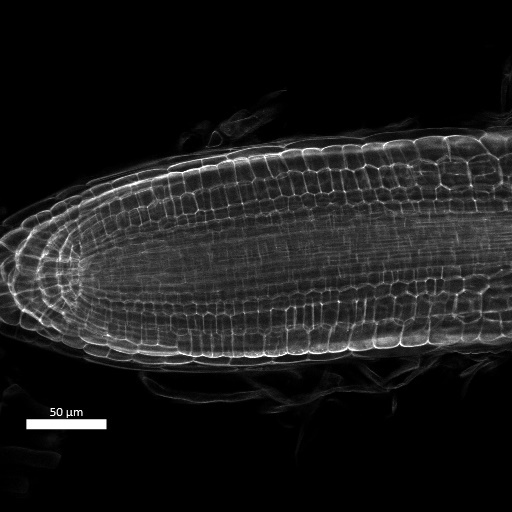

Supplement: Supplementary file 2 — Source Data for Expanded View [file EMBJ-42-e111926-s001.zip › FigureEV5/EV5A/eir1-1_28c.jpg]

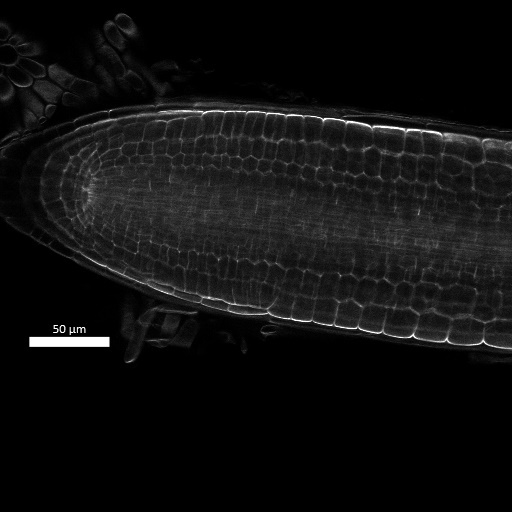

Supplement: Supplementary file 2 — Source Data for Expanded View [file EMBJ-42-e111926-s001.zip › FigureEV5/EV5A/pin4-2_20c.jpg]

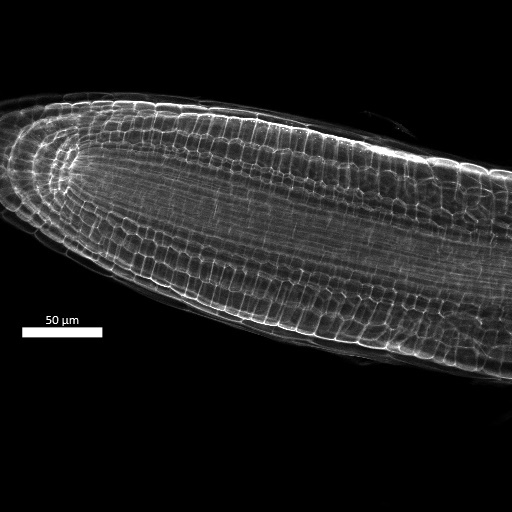

Supplement: Supplementary file 2 — Source Data for Expanded View [file EMBJ-42-e111926-s001.zip › FigureEV5/EV5A/Col-0_28c.jpg]

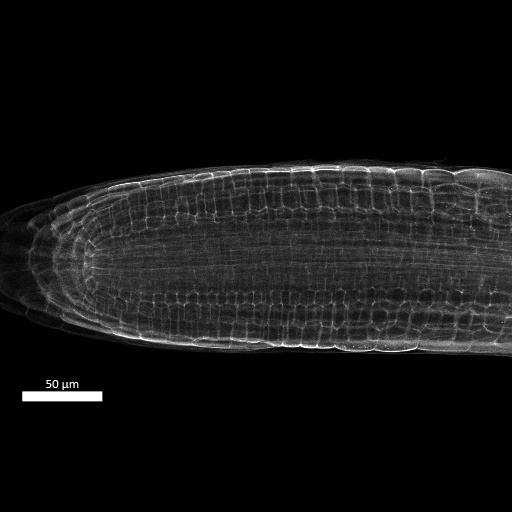

Supplement: Supplementary file 2 — Source Data for Expanded View [file EMBJ-42-e111926-s001.zip › FigureEV5/EV5A/pin1-1_20c.jpg]

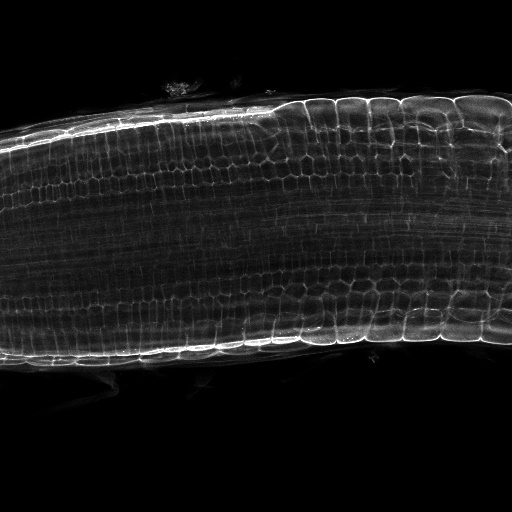

Supplement: Supplementary file 2 — Source Data for Expanded View [file EMBJ-42-e111926-s001.zip › FigureEV5/EV5A/pin4-2_28c_02.jpg]

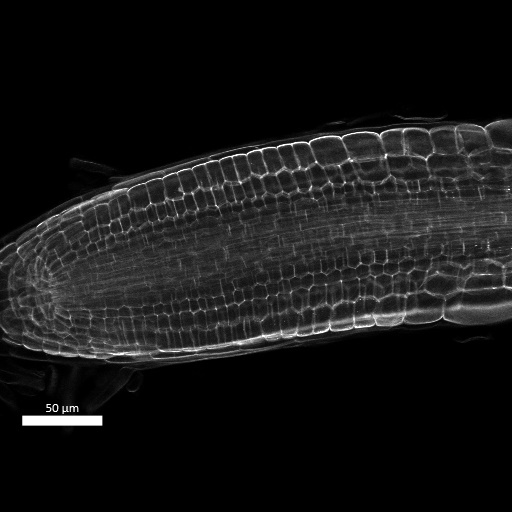

Supplement: Supplementary file 2 — Source Data for Expanded View [file EMBJ-42-e111926-s001.zip › FigureEV5/EV5A/eir1-1_20c.jpg]

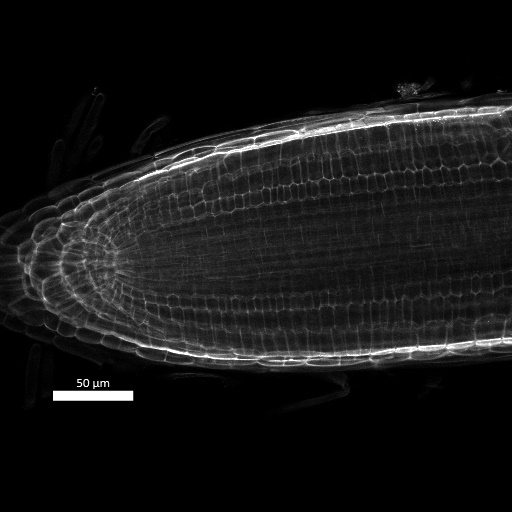

Supplement: Supplementary file 2 — Source Data for Expanded View [file EMBJ-42-e111926-s001.zip › FigureEV5/EV5A/pin4-2_28c_01.jpg]

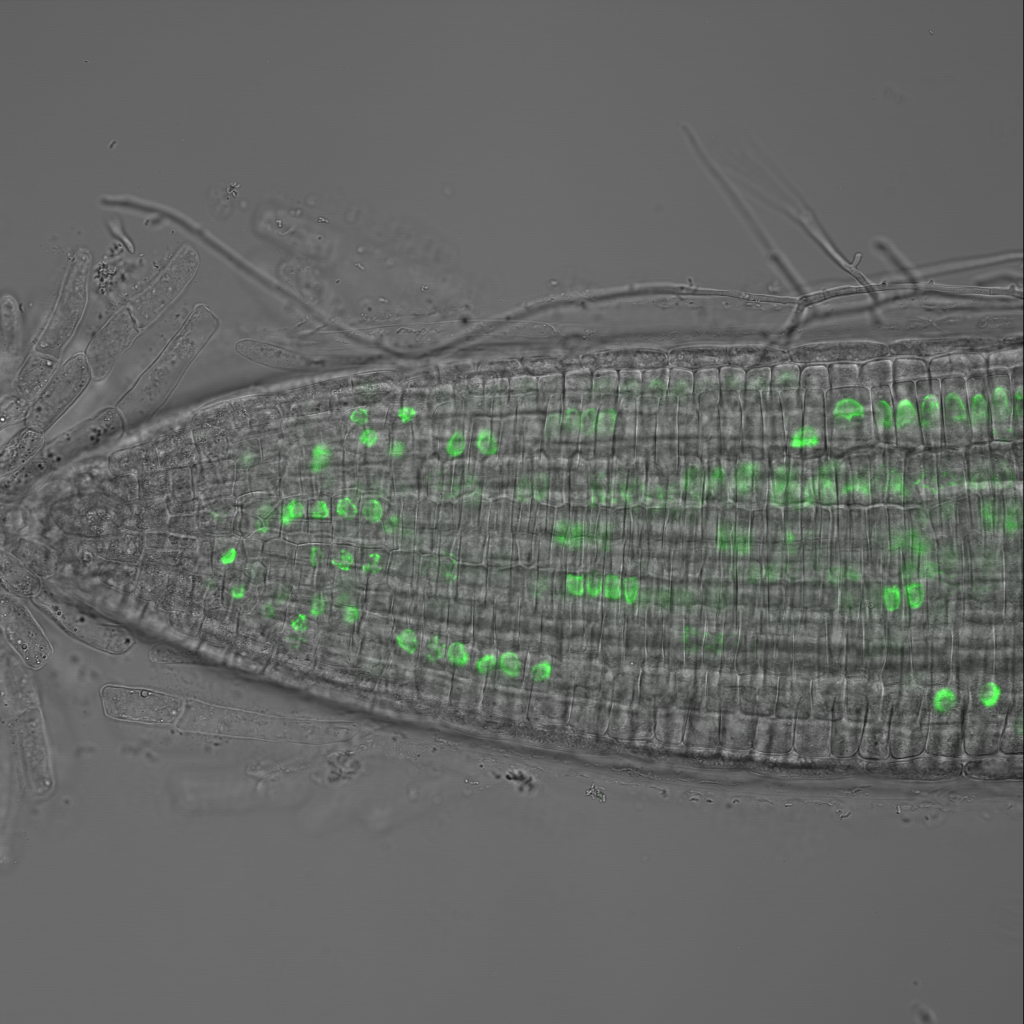

Supplement: Supplementary file 6 — Source Data for Figure 3 [file EMBJ-42-e111926-s009.zip › Figure3/3E/root_microscopy_20c.tif]

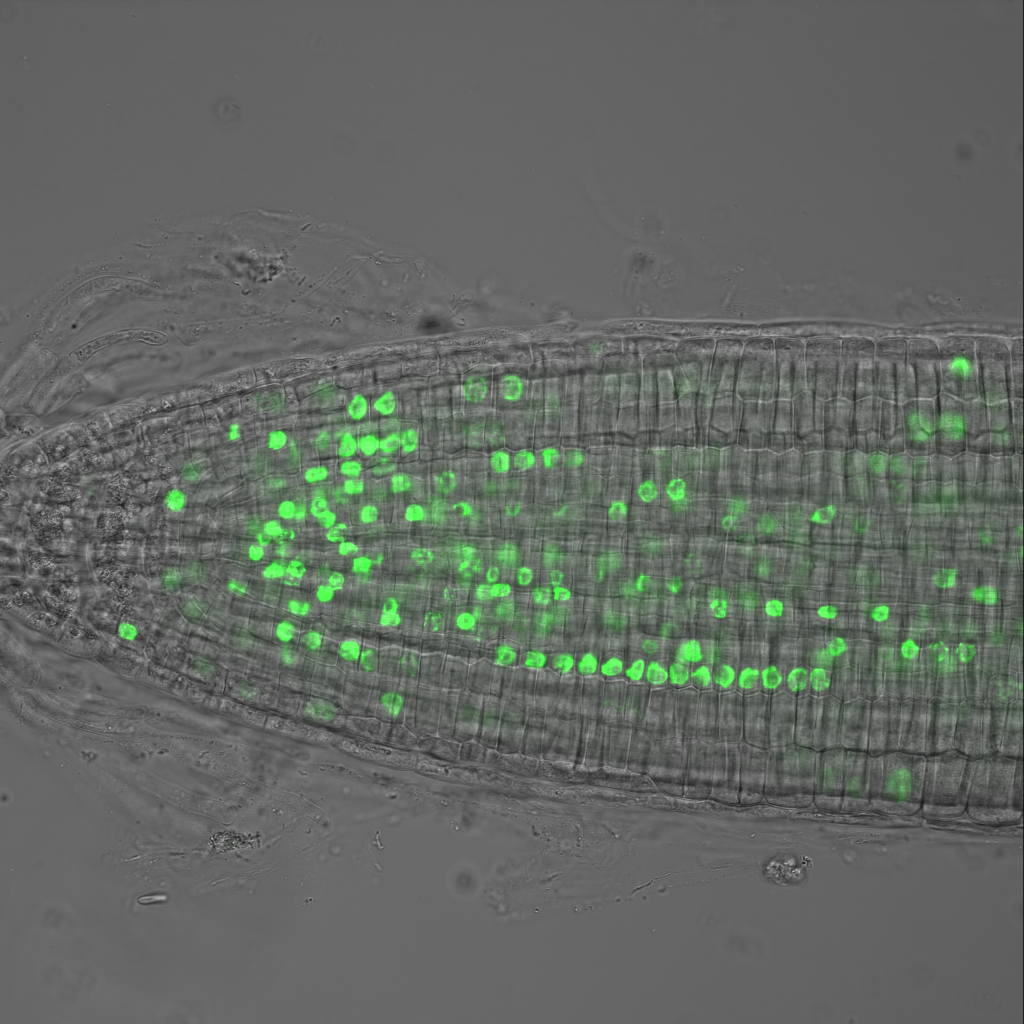

Supplement: Supplementary file 6 — Source Data for Figure 3 [file EMBJ-42-e111926-s009.zip › Figure3/3E/root_microscopy_28c.tif]

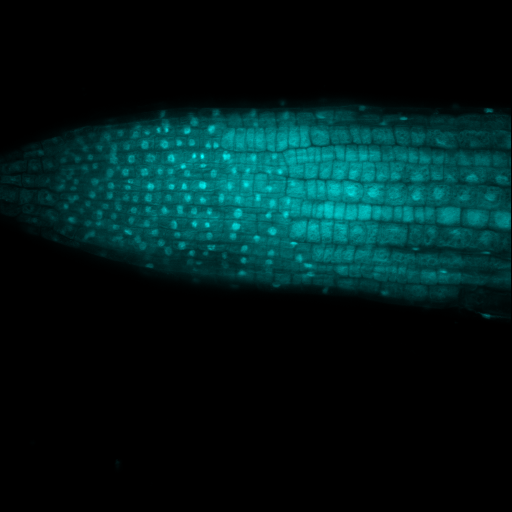

Supplement: Supplementary file 6 — Source Data for Figure 3 [file EMBJ-42-e111926-s009.zip › Figure3/3F/DAPI_microscopy_20c.tif]

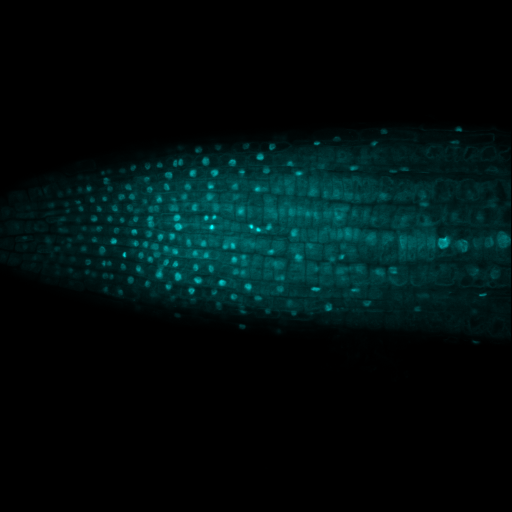

Supplement: Supplementary file 6 — Source Data for Figure 3 [file EMBJ-42-e111926-s009.zip › Figure3/3F/DAPI_microscopy_28c.tif]

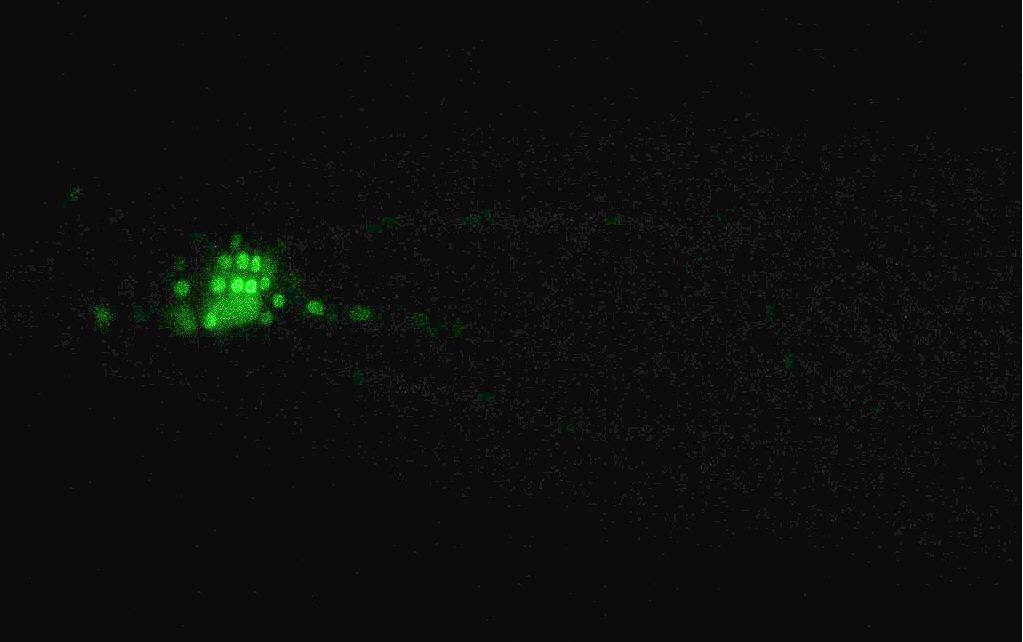

Supplement: Supplementary file 7 — Source Data for Figure 4 [file EMBJ-42-e111926-s006.zip › Figure4/4C/weitar_28C.tiff]

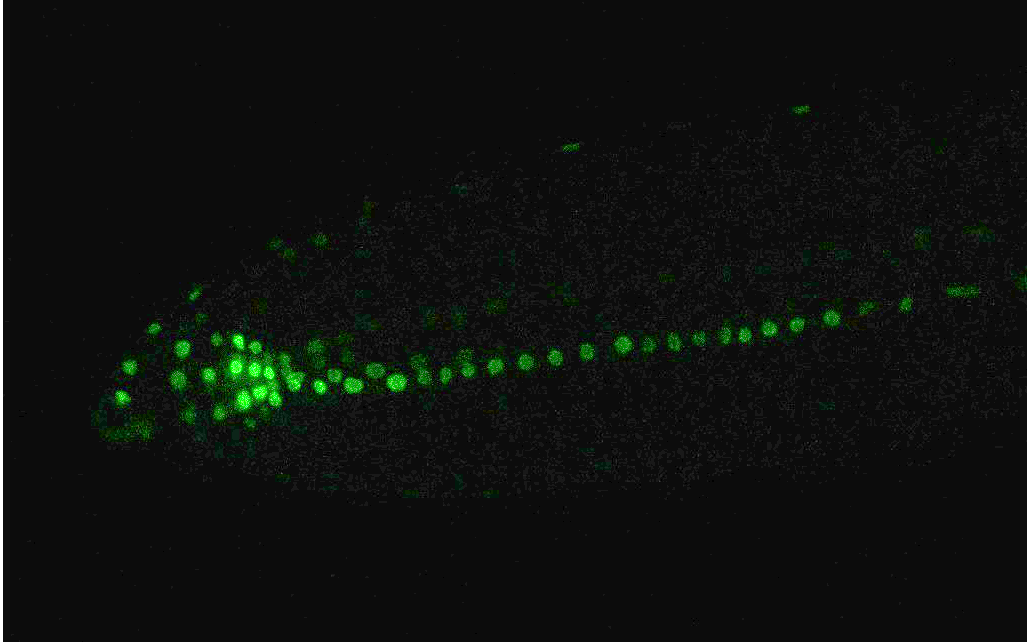

Supplement: Supplementary file 7 — Source Data for Figure 4 [file EMBJ-42-e111926-s006.zip › Figure4/4C/Col_20C.tiff]

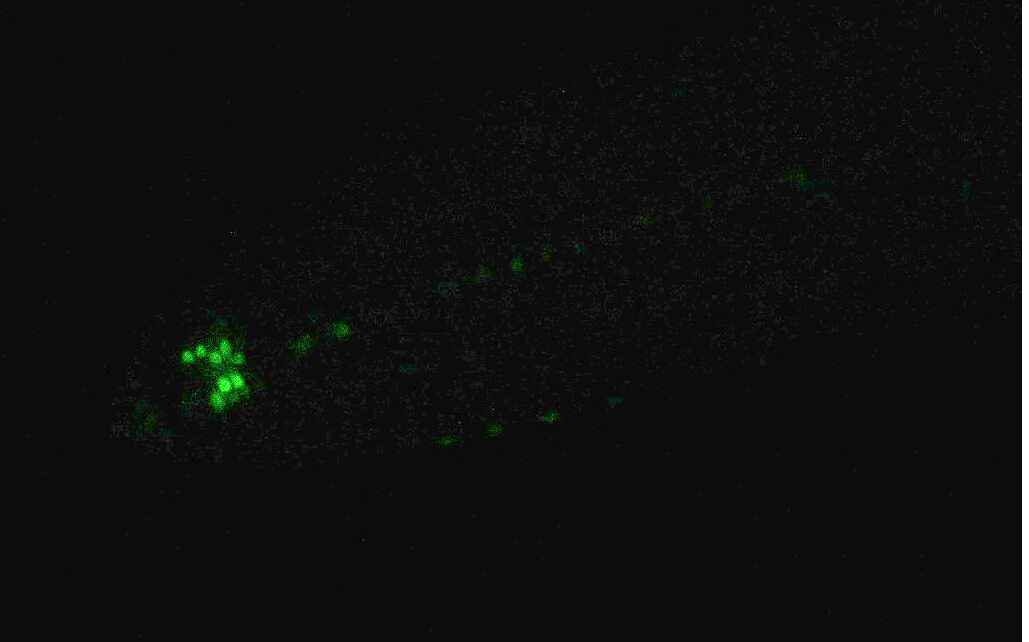

Supplement: Supplementary file 7 — Source Data for Figure 4 [file EMBJ-42-e111926-s006.zip › Figure4/4C/weitar_20C.tiff]

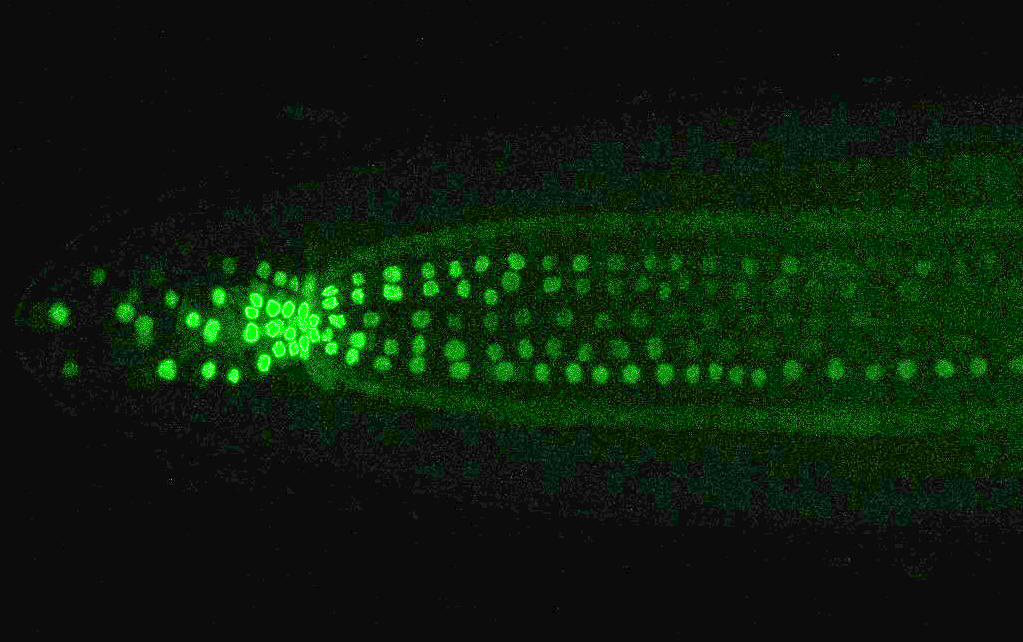

Supplement: Supplementary file 7 — Source Data for Figure 4 [file EMBJ-42-e111926-s006.zip › Figure4/4C/Col_28C.tiff]

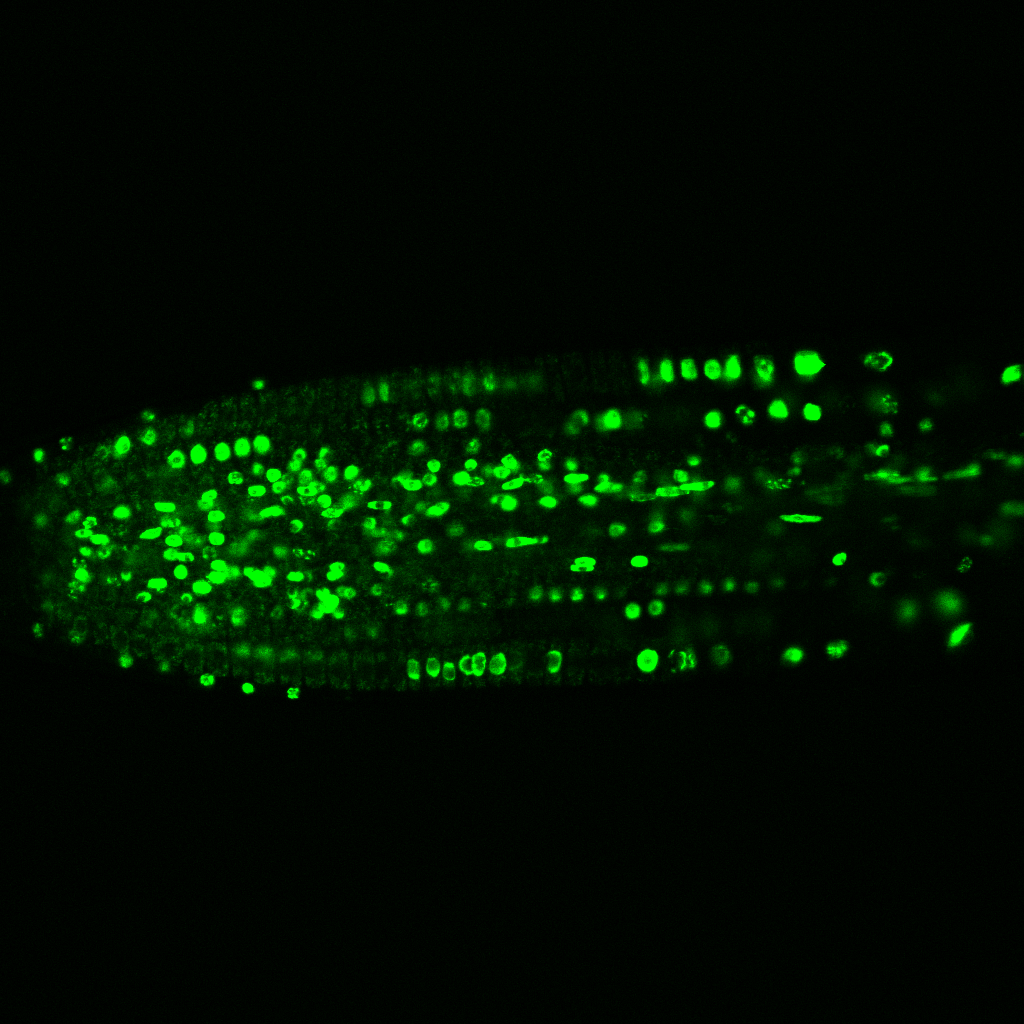

Supplement: Supplementary file 7 — Source Data for Figure 4 [file EMBJ-42-e111926-s006.zip › Figure4/4G/EdU_NAA20c.tif]

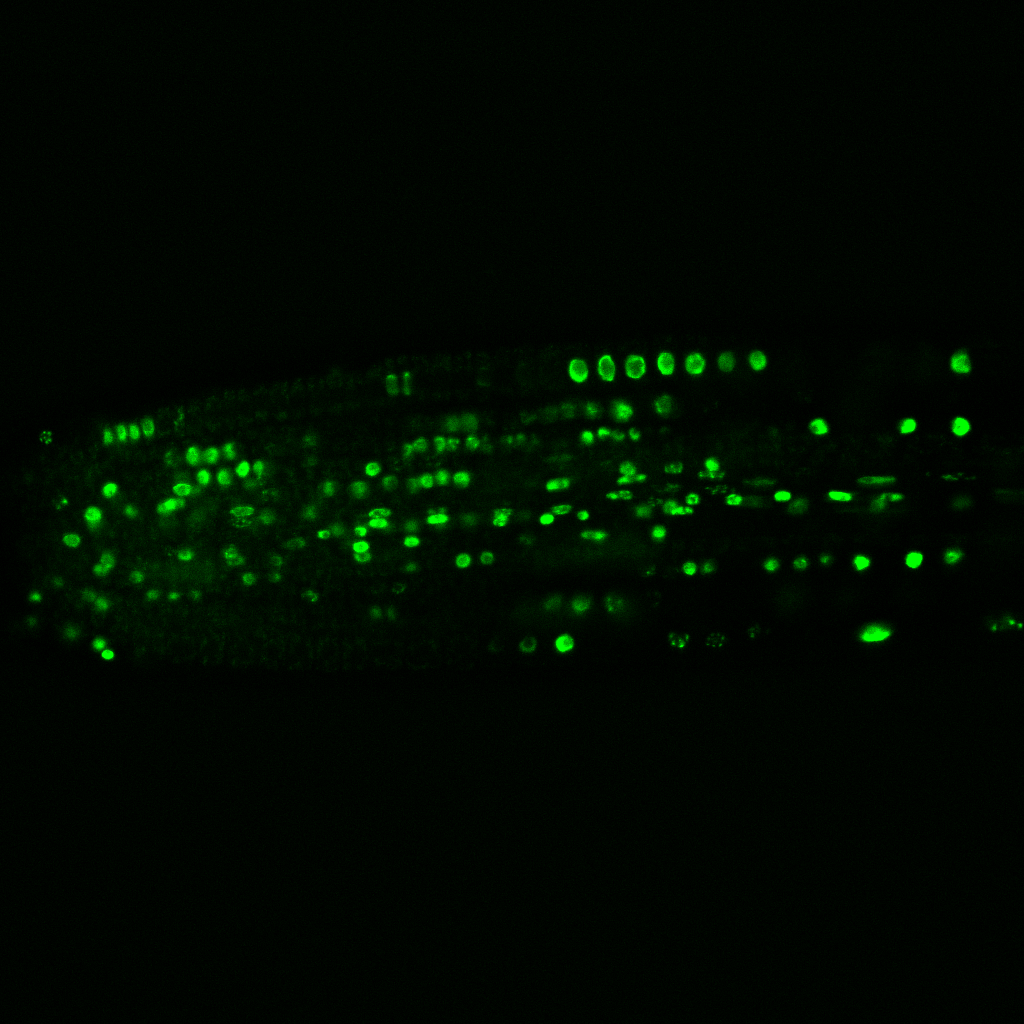

Supplement: Supplementary file 7 — Source Data for Figure 4 [file EMBJ-42-e111926-s006.zip › Figure4/4G/EdU_mock20c.tif]

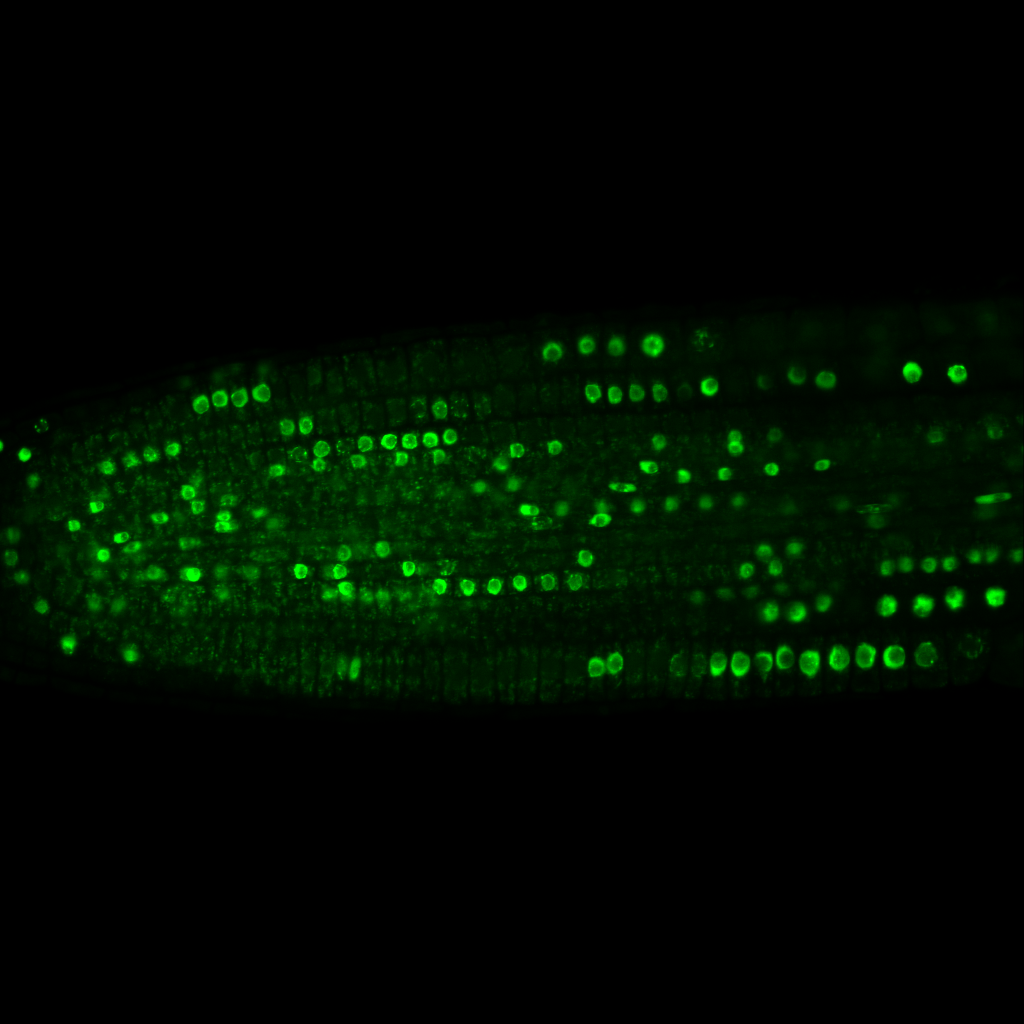

Supplement: Supplementary file 7 — Source Data for Figure 4 [file EMBJ-42-e111926-s006.zip › Figure4/4G/EdU_peoiaa28c.tif]

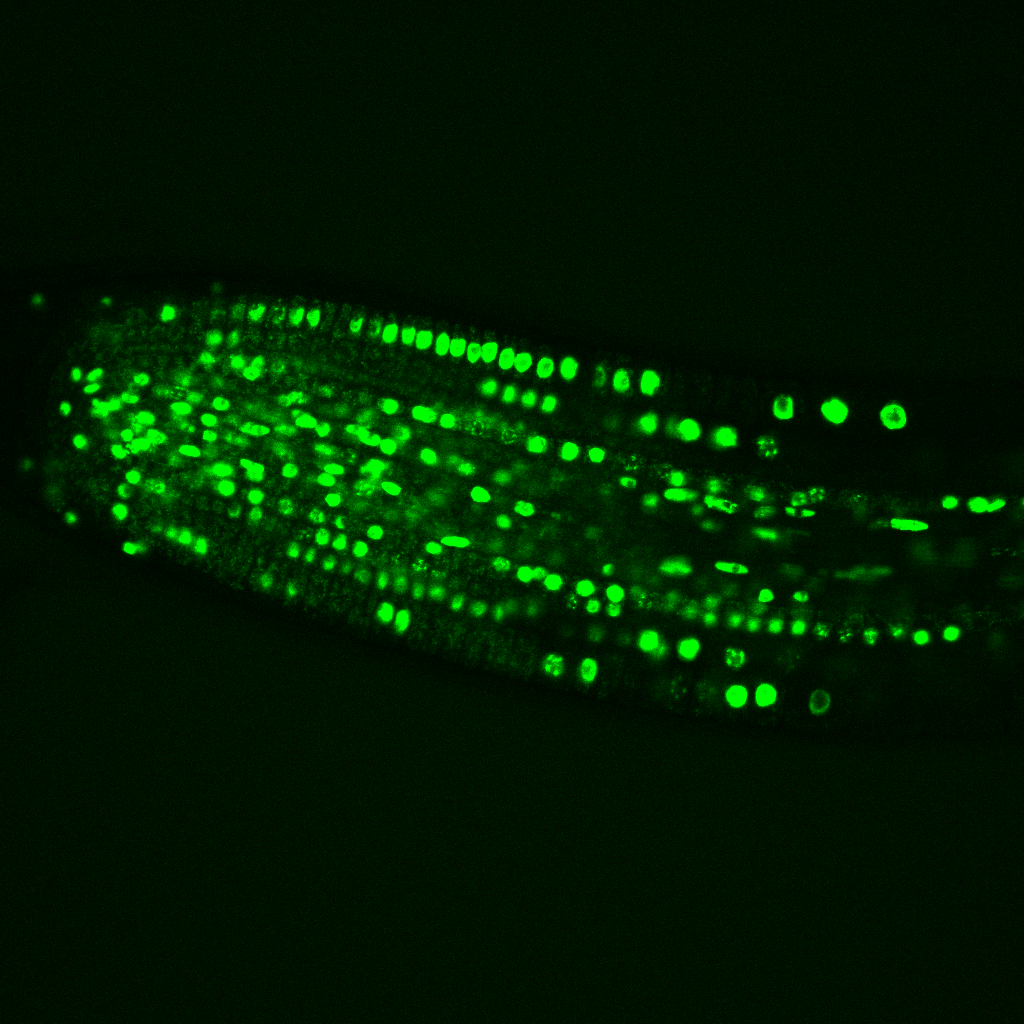

Supplement: Supplementary file 7 — Source Data for Figure 4 [file EMBJ-42-e111926-s006.zip › Figure4/4G/EdU_mock28c.tif]

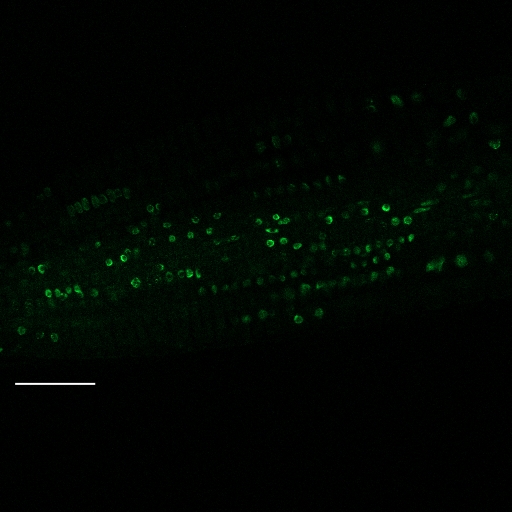

Supplement: Supplementary file 8 — Source Data for Figure 5 [file EMBJ-42-e111926-s005.zip › Figure5/5C/Col_0_20c.jpg]

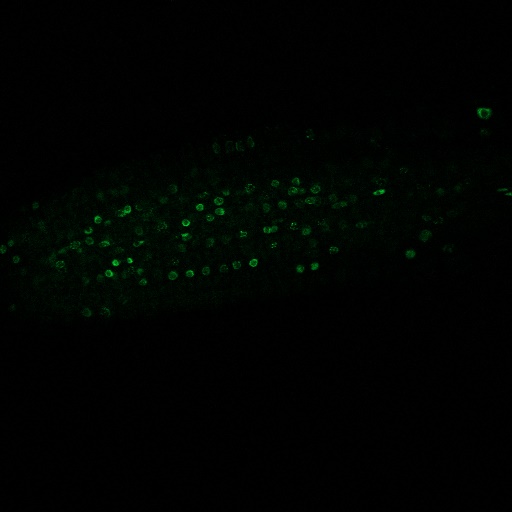

Supplement: Supplementary file 8 — Source Data for Figure 5 [file EMBJ-42-e111926-s005.zip › Figure5/5C/pin1-1_28c.jpg]

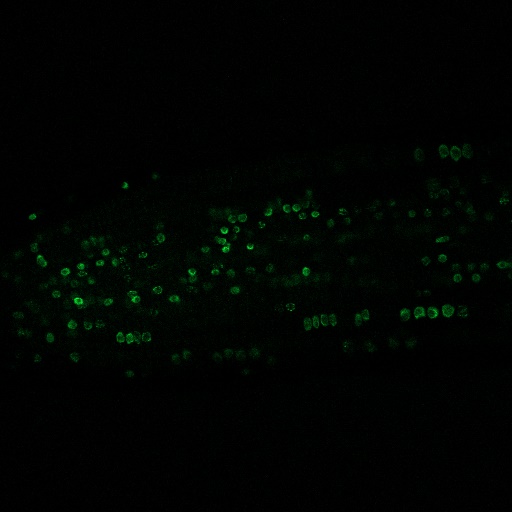

Supplement: Supplementary file 8 — Source Data for Figure 5 [file EMBJ-42-e111926-s005.zip › Figure5/5C/eir1-1_28c.jpg]

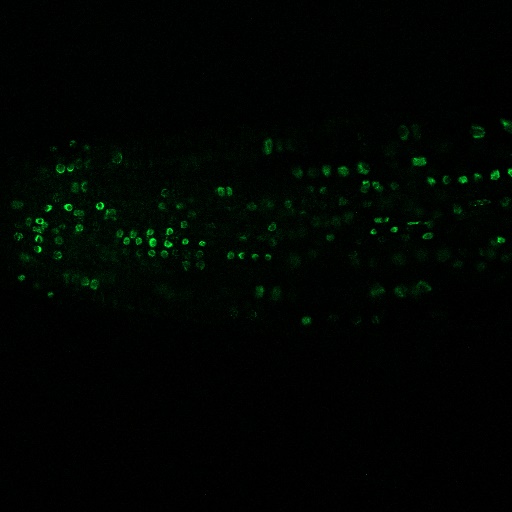

Supplement: Supplementary file 8 — Source Data for Figure 5 [file EMBJ-42-e111926-s005.zip › Figure5/5C/pin4-2_20c.jpg]

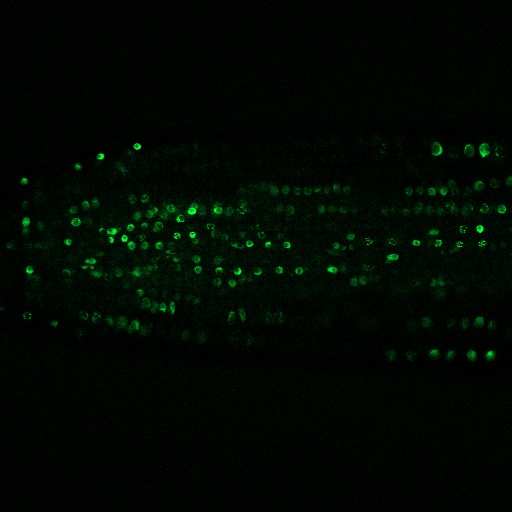

Supplement: Supplementary file 8 — Source Data for Figure 5 [file EMBJ-42-e111926-s005.zip › Figure5/5C/Col_0_28c.jpg]

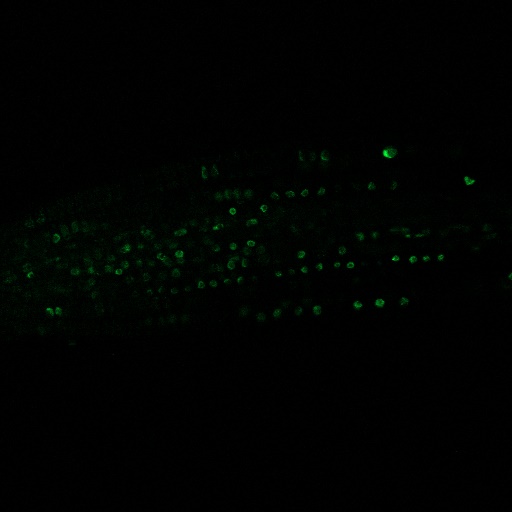

Supplement: Supplementary file 8 — Source Data for Figure 5 [file EMBJ-42-e111926-s005.zip › Figure5/5C/pin1-1_20c.jpg]

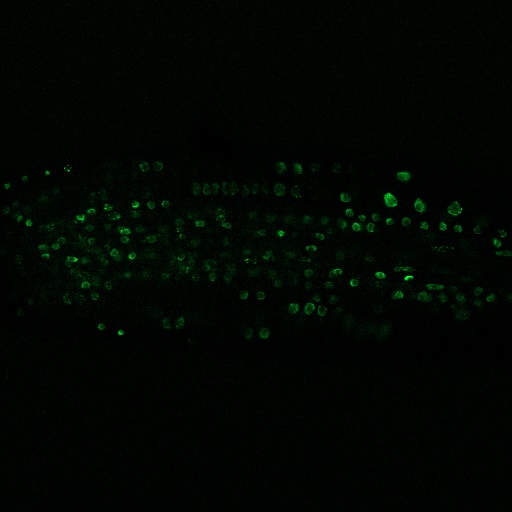

Supplement: Supplementary file 8 — Source Data for Figure 5 [file EMBJ-42-e111926-s005.zip › Figure5/5C/eir1-1_20c.jpg]

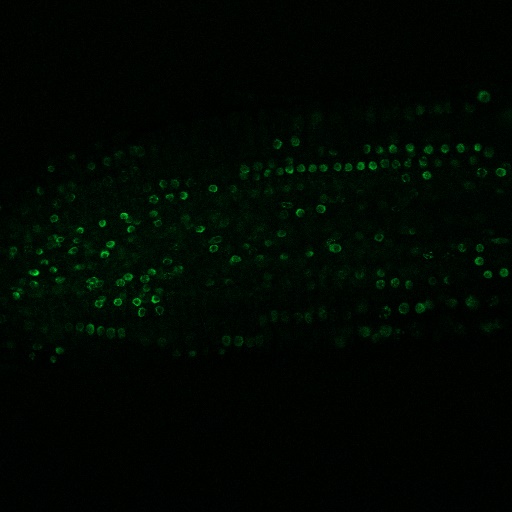

Supplement: Supplementary file 8 — Source Data for Figure 5 [file EMBJ-42-e111926-s005.zip › Figure5/5C/pin4-2_28c.jpg]

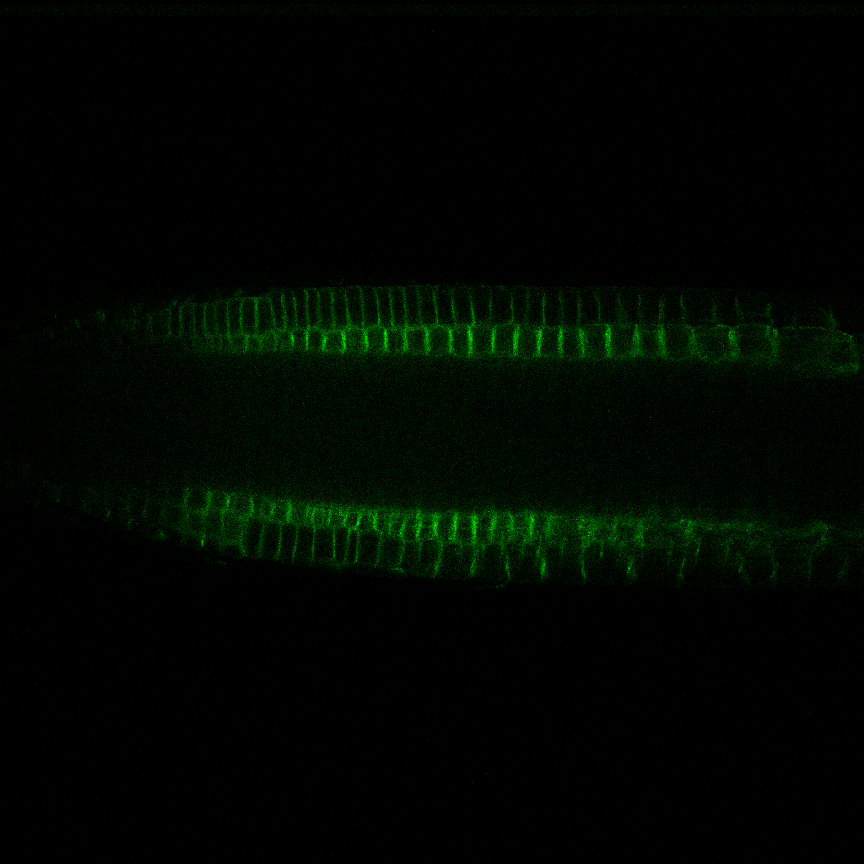

Supplement: Supplementary file 8 — Source Data for Figure 5 [file EMBJ-42-e111926-s005.zip › Figure5/5E/PIN2GFP_28C.tif]

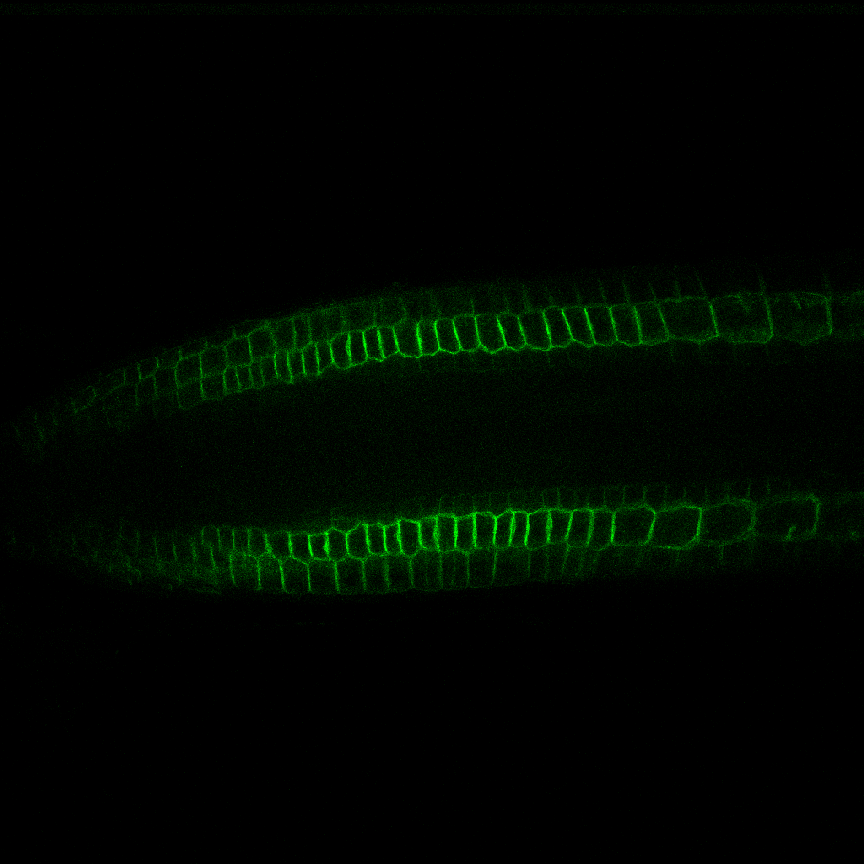

Supplement: Supplementary file 8 — Source Data for Figure 5 [file EMBJ-42-e111926-s005.zip › Figure5/5E/PIN2GFP_20C.tif]

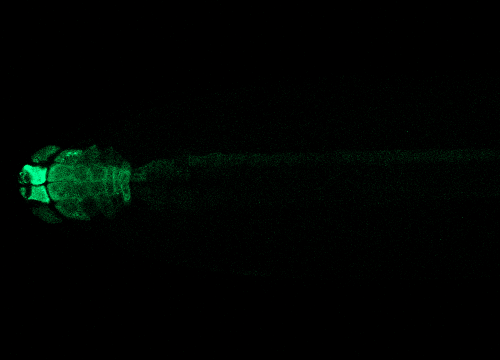

Supplement: Supplementary file 8 — Source Data for Figure 5 [file EMBJ-42-e111926-s005.zip › Figure5/5B/pin1_20C.tiff]

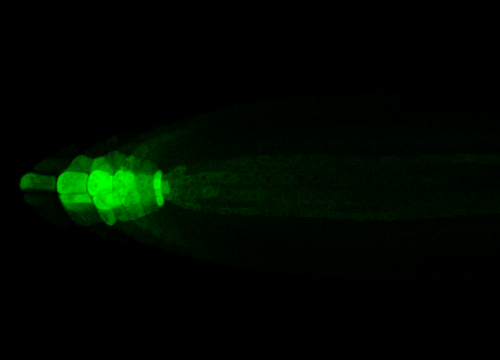

Supplement: Supplementary file 8 — Source Data for Figure 5 [file EMBJ-42-e111926-s005.zip › Figure5/5B/pin4_28C.tiff]

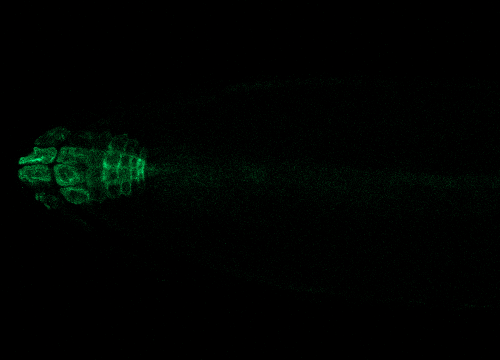

Supplement: Supplementary file 8 — Source Data for Figure 5 [file EMBJ-42-e111926-s005.zip › Figure5/5B/col_20C.tiff]

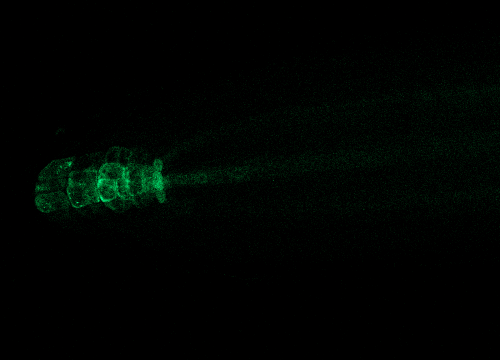

Supplement: Supplementary file 8 — Source Data for Figure 5 [file EMBJ-42-e111926-s005.zip › Figure5/5B/pin1_28C.tiff]

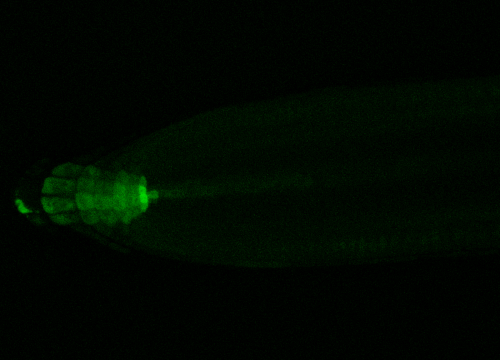

Supplement: Supplementary file 8 — Source Data for Figure 5 [file EMBJ-42-e111926-s005.zip › Figure5/5B/pin4_20C.tiff]

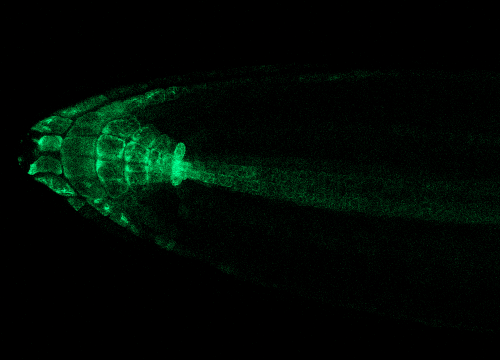

Supplement: Supplementary file 8 — Source Data for Figure 5 [file EMBJ-42-e111926-s005.zip › Figure5/5B/col_28C.tiff]
