# Supplementary material for: Auxin‐dependent regulation of cell division rates governs root thermomorphogenesis
Source: EMBO J. 2023 Apr 18;42(11):e111926. doi: 10.15252/embj.2022111926 (PMC10233379; doi:10.15252/embj.2022111926)
Supplement: Supplementary file 3 — PDF+ [file EMBJ-42-e111926-s003.pdf]

Article

SOURCE DATA | TRANSPARENT PROCESS | OPEN ACCESS

THE EMBO JOURNAL

Auxin-dependent regulation of cell division rates governs root thermomorphogenesis

Haiyue Ai[1], Julia Bellstaedt[1], Kai Steffen Bartusch[2], Lennart Eschen-Lippold[1], Steve Babben[1], Gerd Ulrich Balcke[3], Alain Tissier[3], Bettina Hause[3], Tonni Grube Andersen[4], Carolin Delker[1] & Marcel Quint[1,*]

Abstract

Roots are highly plastic organs enabling plants to adapt to a changing below-ground environment. In addition to abiotic factors like nutrients or mechanical resistance, plant roots also respond to temperature variation. Below the heat stress threshold, *Arabidopsis thaliana* seedlings react to elevated temperature by promoting primary root growth, possibly to reach deeper soil regions with potentially better water saturation. While above-ground thermomorphogenesis is enabled by thermo-sensitive cell elongation, it was unknown how temperature modulates root growth. We here show that roots are able to sense and respond to elevated temperature independently of shoot-derived signals. This response is mediated by a yet unknown root thermosensor that employs auxin as a messenger to relay temperature signals to the cell cycle. Growth promotion is achieved primarily by increasing cell division rates in the root apical meristem, depending on *de novo* local auxin biosynthesis and temperature-sensitive organization of the polar auxin transport system. Hence, the primary cellular target of elevated ambient temperature differs fundamentally between root and shoot tissues, while the messenger auxin remains the same.

Keywords *Arabidopsis thaliana*; auxin; cell division; root; thermomorphogenesis
Subject Category Plant Biology
DOI 10.15252/embj.2022111926 | Received 21 June 2022 | Revised 16 March 2023 | Accepted 21 March 2023 | Published online 18 April 2023
The EMBO Journal (2023) 42: e111926

Introduction

With increasing concern over global warming, awareness of its impact on living organisms has steadily grown in the past 30 years. Plants can acclimate to warm temperatures with wide-ranging physiological and morphological adjustments. These acclimation processes can enhance plant fitness, but high ambient temperatures still exert a negative effect on plant growth and development, and likewise on crop yields (Lippmann *et al*, 2019). Thermomorphogenesis describes these acclimations and thereby the effect of ambient temperature on plant morphogenesis (Delker *et al*, 2014). In the model plant *Arabidopsis thaliana*, hypocotyl elongation and increased leaf hyponasty are among the earliest thermomorphogenic changes in response to elevated temperatures (reviewed in Quint *et al*, 2016; Casal & Balasubramanian, 2019). Such morphological adjustments result in an open rosette structure with increased ventilation enabling evaporative cooling which effectively reduces leaf temperatures (Crawford *et al*, 2012). Across land plants, the ecophysiological advantage of these acclimation responses may be to confer some sort of metabolic plasticity, mitigating the effects of high temperature (Ludwig *et al*, 2021). The major signaling hub controlling these morphological responses in *A. thaliana* is the basic helix–loop–helix transcription factor PHYTOCHROME-INTERACTING FACTOR 4 (PIF4) (Koini *et al*, 2009). In concert with downstream hormonal action (reviewed in Castroverde & Dina, 2021), PIF4 transcriptionally regulates genes promoting cell elongation. PIF4 itself is regulated by various thermosensors. At low temperature, PIF4 is repressed by numerous mechanisms that affect PIF4 gene expression, protein levels, and activity. Important thermosensors that directly repress PIF4 are PHYTOCHROME B (PHYB, Jung *et al*, 2016; Legris *et al*, 2016) and EARLY FLOWERING 3 (ELF3, Jung *et al*, 2020). High temperature relieves this inhibition and induces *PIF4* transcription and other PIF4 regulatory mechanisms (reviewed in Delker *et al*, 2022), opening the route to thermo-induced elongation growth. So far, these discoveries describe thermomorphogenic responses only in shoot tissues.

Similar to hypocotyls and petioles, seedling roots likewise respond to elevated ambient temperature with growth promotion (e.g., Quint *et al*, 2005; Quint *et al*, 2009; Hanzawa *et al*, 2013; Wang *et al*, 2016; Ibañez *et al*, 2017; Martins *et al*, 2017; Feraru *et al*, 2019; Gaillochet *et al*, 2020; reviewed in de Lima *et al*, 2021). While shoot thermomorphogenesis is meanwhile reasonably well understood with regard to both ecophysiological as well as signaling levels, root thermomorphogenesis is not. Given that in natural

1 Institute of Agricultural and Nutritional Sciences, Martin Luther University Halle-Wittenberg, Halle (Saale), Germany
2 Department of Biology, Institute of Molecular Plant Biology, ETH Zürich, Zürich, Switzerland
3 Department of Cell and Metabolic Biology, Leibniz Institute of Plant Biochemistry, Halle (Saale), Germany
4 Max Planck Institute for Plant Breeding Research, Cologne, Germany
*Corresponding author. Tel: +49 0345 5522739; E-mail: marcel.quint@landw.uni-halle.de

© 2023 The Authors. Published under the terms of the CC BY 4.0 license.

habitats warm top soils often co-occur with drought, it makes sense to seek the ecophysiological explanation for this response in the exploration of deeper soil layers to reach potentially available water resources (Martins *et al*, 2017; Ludwig *et al*, 2021). Knowledge about physiological mechanisms regulating temperature-responsive elongation growth in roots is, however, rather fragmentary. It is, for example, unclear whether temperature-responsive root growth is primarily a consequence of promotion of cell elongation, as in hypocotyls and petioles, or whether it is more likely to be caused by increased rates of cell division or a combination thereof. Classic cytological studies in various plant species support a promoting effect of increasing temperature on cell division rates most likely by affecting cell cycle duration (Erickson, 1959; Murin, 1966, 1981; Silk *et al*, 1989; Silk, 1992). A large metastudy on the duration of the cell cycle of 170 angiosperms supports this observation (Grif *et al*, 2002). In fact, Grif & Valovich (1973) already argued 50 years ago that the only reason for root growth retardation at low temperature is the extension of cell cycle duration. Turning this argument around, it would predict that growth promotion at high temperature is caused by accelerating the cell cycle and thereby increasing rates of cell division. While a recent and very elegant kinematic study on Arabidopsis roots supports the promotional effect of temperature on cell division rates (Yang *et al*, 2017), the authors, however, interpreted this as a compensation for a shorter meristematic zone and suggested a significant role for increased cell elongation in root growth promotion at elevated temperatures. Shortening of the Arabidopsis root apical meristematic zone at elevated temperatures has also been reported by Feraru *et al* (2019), while Zhu *et al* (2015) observed the same phenomenon for lower temperatures, and Hanzawa *et al* (2013) described a larger area of cell division activity and increased cell flux at high temperature. Given these partially contradicting observations, a conclusive picture has yet to emerge. Although conflicting data have been reported also on hormonal regulation of root thermomorphogenesis (Martins *et al*, 2017), it seems to be consensus that auxin plays a central role. In support of this, nuclear auxin responses as measured by reporter assays increase with temperature (Hanzawa *et al*, 2013; Feraru *et al*, 2019; Gaillochet *et al*, 2020), and selected auxin-related mutants have been reported to show (mostly mild) defects in temperature-induced root elongation (Wang *et al*, 2016; Feraru *et al*, 2019; Gaillochet *et al*, 2020). Auxin-related mutants with severe, and ideally conditional, phenotypes for temperature-induced root elongation are rare. While this may be due to genetic redundancy and/or pleiotropy of important players, it makes it difficult to genetically evaluate auxin's importance for this response, which may be anywhere between being essential to being moderate. Likewise, the downstream processes and the precise cellular mechanism via which auxin controls root growth at elevated temperature are yet elusive.

Taken together, the vast majority of studies that have addressed root thermomorphogenesis in the past each focus on a specific aspect which is analyzed in great detail. A comprehensive model spanning temperature perception, signaling, the function of auxin in such a signaling pathway and the cellular process which regulates root growth is as of yet lacking. In this study, we, therefore, aim to develop such a comprehensive model and ask the following questions: Can roots sense temperature autonomously or do they rely on shoot–root communication of signals that may have been perceived in the shoot? Is temperature-induced root elongation caused by increased cell elongation or cell division or a combination thereof? How is the temperature signal transduced from a sensor to the cellular growth response? How important is auxin in root thermomorphogenesis and what is its mechanistic role? Answers to these questions may enable connecting the above-described dots of fragmentary knowledge to derive a comprehensive mechanistic working model of the primary physiological process regulating root thermomorphogenesis.

Results

Root growth dynamics in response to different temperatures

Based on recently published work from other labs (e.g., Martins *et al*, 2017; Feraru *et al*, 2019; Gaillochet *et al*, 2020; Lee *et al*, 2021) and our own previous comprehensive phenotypic analyses (including root growth) of various *A. thaliana* accessions grown in a range of different ambient temperatures (Ibañez *et al*, 2017), we tried to better understand how temperature specifically affects root growth. In the temperature range investigated (20 vs. 28°C), elevated ambient temperature promoted primary root elongation in not only *A. thaliana* accession Col-0 (Fig 1A and B) but also across eudicots (Fig 1C and D), demonstrating that temperature-induced root elongation is a universal response. Daily measurements of root length in *A. thaliana* suggested that growth differences were absent in the first 4 days of cultivation (Fig 1B). To substantiate this observation, we captured root growth rates continuously between days 2 and 7 after germination. Infrared imaging enabled hourly documentation of growth behavior also in darkness. Similar to the end-point analysis presented in Fig 1B, we observed differential growth rates only after day 4 (Fig EV1A). Independent of temperature, root growth rates displayed a pronounced diurnal fluctuation. Under the long-day photoperiods applied, root growth rates increased in the late afternoon and peaked at the end of the night, which was mirrored by high-temperature hypocotyl growth patterns in the same plants (Fig EV1B). These data indicated that (under our conditions) root growth rates increase during early seedling development, but temperature sensitivity of growth rates seems to be gated during the first few days.

Roots are independently sensing and responding to elevated ambient temperature

We had previously suggested that roots are able to autonomously sense and respond to temperature without the need for shoot-derived signals (Bellstaedt *et al*, 2019). However, Gaillochet *et al* (2020) proposed a central role of shoot-to-root communication for root thermomorphogenesis. To revisit this question and to assess whether or not the root response requires such long-distance signals from a temperature-sensing shoot, we first tested temperature-induced root elongation assays with excised roots (only the root, no shoot attached) in Col-0. In confirmation of our previously published data (Bellstaedt *et al*, 2019), we found that detached roots were not only able to continue growing in the absence of shoot tissue, they even elongated more at 28°C than at 20°C (Fig 2A). This suggests that roots can be regarded as autonomous in terms of

© 2023 The Authors

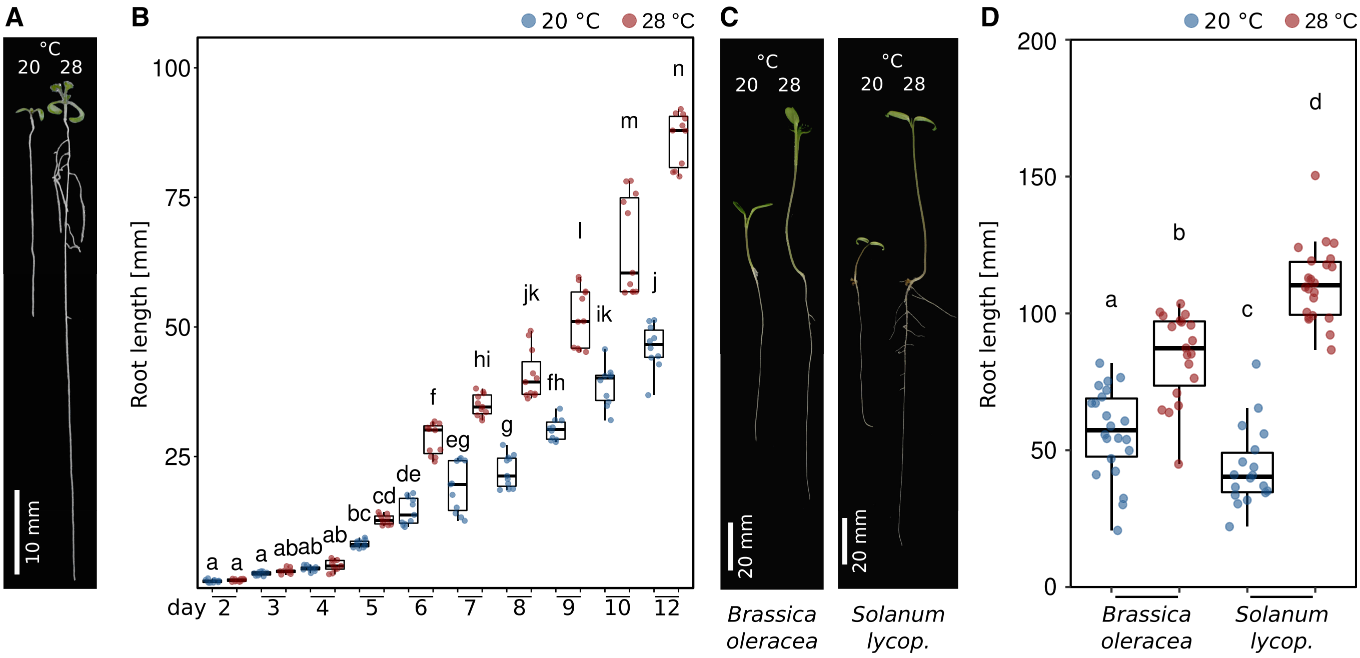

Figure 1. Root growth dynamics in response to different temperatures.

A Arabidopsis seedlings grown for 7 days in LD at 20 or 28°C.

B Root lengths of seedlings from days 2 to 12 after sowing (grown as in A).

C Seedlings of *Brassica oleracea* and *Solanum lycopersicum* (lycop.) grown for 7 days at 20 or 28°C.

D Root lengths of 7-day-old seedlings grown at 20 or 28°C (n = 18–22). (B, D) Boxplots show medians, interquartile ranges, and min–max values with individual data points superimposed as colored dots. Different letters denote statistical differences at $P < 0.05$ assessed by two-way ANOVA and Tukey's HSD *post hoc* test. The data are representative of two independent experiments.

Source data are available online for this figure.

sensing and responding to temperature cues. This behavior was similar in *Brassica oleracea* and *Solanum lycopersicum* (Fig 2A), indicating its conservation across species.

As this assay is rather "rude," we next performed a likewise invasive but certainly less drastic hypocotyl micrografting assay to substantiate these results, also in different genetic backgrounds (Fig EV2A). Here, a shoot mutant that is unable to transduce temperature cues would be informative, as in such a genetic background the relay of temperature-induced signals from the shoot to the root is unlikely. For these experiments, we first used the essentially temperature-blind quadruple *pifQ* (*pifQ* = *pif1-1 pif3-7 pif4-2 pif5-3*) mutant. Fig 2B shows that all possible *pifQ* and wt scion > rootstock combinations displayed a wild type-like root elongation in response to elevated temperature. Not surprisingly, this behavior was mirrored by a *pif4-2* single mutant (Fig EV2B). Likewise, *hy5-51* mutant shoots on wild-type rootstocks behaved like wild type, arguing against a role for shoot-localized or shoot-derived HY5 in root thermomorphogenesis (Fig 2C). Similarly, wild-type shoots on *hy5-51* roots did not differ from the wild-type self-graft. Only *hy5-51* self-grafts showed shorter roots at both temperatures (Fig 2C). Together, these data argue for a general role of *HY5* in root growth, albeit under our conditions not in a temperature context. A mutant with a potential shoot-to-root effect in these experiments was the shoot thermosensory mutant *phyB-9*. While wild-type shoots on *phyB-9* rootstocks did not differ from the wild-type self-grafts, graft combinations including *phyB-9* shoots (on either wild-type or *phyB-9* rootstocks) displayed significantly shorter roots at high temperature when compared to graft combinations with wild-type shoots (Fig 2D). However, these grafting combinations were still able to respond to the temperature stimulus, suggesting a rather minor role for shoot localized or derived PHYB in this process. It has to be noted that the growth conditions and times needed for grafting assays (Fig EV2A) differ from the experimental setup for intact seedlings. Together with the still invasive character of micrografting and some hypocotyl tissue that remains with the rootstock, this should be taken into account when interpreting these data. Furthermore, we are aware that at this point there is no true negative control available showing a severe root elongation defect in response to temperature. Taken together, although a shoot-derived function of PHYB cannot be ruled out, the thermo-responsive behavior of micrografted seedlings with thermo-sensing/-signaling defective scions on wild-type rootstocks largely supports an autonomous character of roots in terms of their capability to sense and respond to elevated ambient temperature.

In intact seedlings, we found that none of the shoot thermomorphogenesis mutants tested (*phy* loss- and gain-of-function lines, *cop1-6*, *elf3-1*, and *pif* loss-of-function, and overexpression lines) showed a root response defect that was remotely reminiscent of their severe shoot phenotypes (Fig EV2C and D; Delker *et al*, 2014; Jung *et al*, 2016; Legris *et al*, 2016). While we did observe statistically significant differences for selected mutant lines in comparison to the wild type (see also Gaillochet *et al*, 2020), these differences

© 2023 The Authors

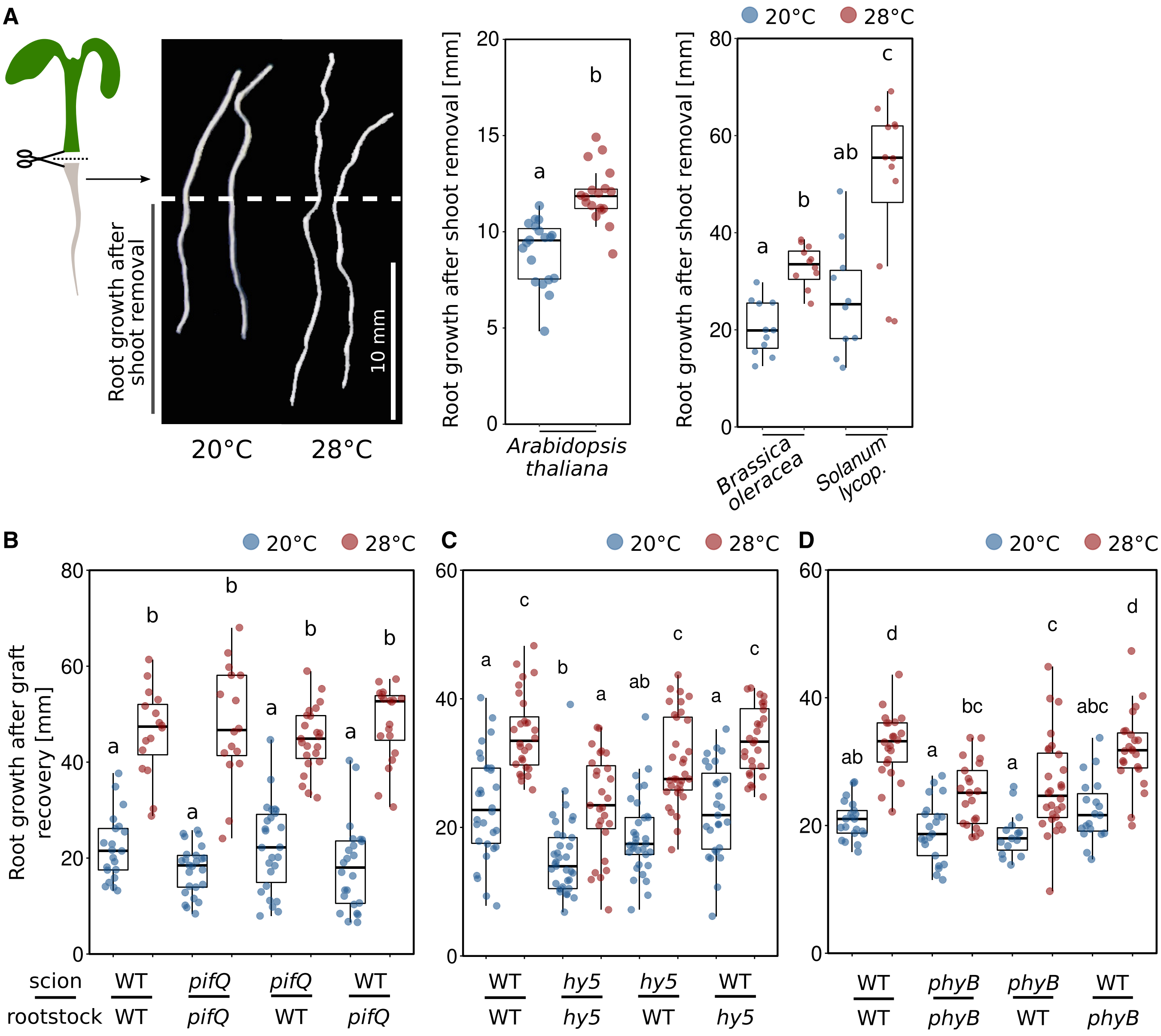

Figure 2. The root is able to autonomously sense temperature and respond to it.

A Elongation responses of detached roots. Shoots were removed from 4-day-old *Arabidopsis thaliana* (n = 18–19), 4-day-old *Brassica oleracea* (n = 11), and 5-day-old *Solanum lycopersicum* (lycop., n = 10–12) seedlings grown at 20°C. Detached roots were grown for additional 4 days at 20 or 28°C, scale bar = 10 mm.

B–D Nine-day-old seedlings were hypocotyl grafted, recovered for 7 days, and then cultivated at 20 or 28°C for additional 7 days (n = 16–26). (A–D) Boxplots show medians, interquartile ranges, and min–max values with individual data points superimposed as colored dots. Different letters denote statistical differences at $P < 0.05$ as assessed by one-way (A, Arabidopsis) or two-way (A–D) ANOVA and Tukey's HSD *post hoc* test. The data are representative of two (A) or three (B–D) independent experiments.

Source data are available online for this figure.

were mostly subtle. All tested lines were still able to sense elevated temperatures and respond with root growth promotion. Again, unavailability of a temperature-unresponsive control complicates interpretation of these data. It has to be noted, however, that others have provided solid evidence, suggesting that at least HY5 seems to play a role in root thermomorphogenesis, albeit as a positive regulator in contrast to its repressive function in the shoot (Gaillochet *et al*, 2020; Lee *et al*, 2021). Taken together, under the expectation of severe phenotypes in loss-of-function backgrounds of important signaling components, we conclude so far that roots are able to autonomously sense and respond to temperature, most likely via a signaling pathway that is different from the pathway regulating the shoot temperature response. While a role for phytochrome-dependent thermo-signaling, which dominates shoot thermomorphogenesis, cannot be generally ruled out, this role is likely of secondary or indirect nature in thermo-responsive root growth.

 © 2023 The Authors

Temperature-induced root growth is largely due to promotion of cell proliferation

Theoretically, temperature-induced primary root elongation may be a consequence of temperature-promoted cell elongation (as in the shoot), temperature-promoted cell division or a combination thereof. To assess the potential of both processes in our experimental setup, we first compared the number of hypocotyl or radicle/root cells in mature embryos versus 7-day-old seedlings (Col-0). We observed that the number of cortical hypocotyl cells along a longitudinal cell file from the root–shoot junction to the shoot apical meristem was only marginally larger in seedlings compared to mature embryos, with a significant but minor temperature effect (Fig EV3A). This incremental increase in hypocotyl cell numbers during early seedling development practically leaves only cell elongation as the primary mechanism of vertical organ growth in response to warmth. Cell division can therefore be neglected. In roots, the picture changed dramatically. The embryonic radicle consists of only a few cells, while cell division generates hundreds of new root cells post germination along a longitudinal cell file (Fig EV3B), and therefore thousands of cells in the whole three-dimensional root. The number of root cells further increased at 28°C. This indicates that cell division has the potential to contribute substantially to thermo-responsive root growth.

We, therefore, investigated cellular growth patterns in more detail and first measured the length of the meristematic zone, the elongation zone, and the remaining part of the root up to the root–shoot junction (referred to as differentiation zone) on a daily basis from days 2 to 7 after germination. For the meristematic zone, we find that 28°C grown seedlings displayed a slightly longer meristematic zone until day 5 when meristem length seemed to level out (Fig 3A). After day 5, only meristems of 20°C grown seedlings elongated further resulting in a slightly longer meristematic zone after day 7. While we regard these differences as subtle and therefore minor, we are aware that they may explain some of the discrepancies in the literature regarding temperature effects on the size of the root apical meristem. The length of the elongation zone behaved similarly with likewise only minor differences between temperatures (Fig 3B). In contrast, the differentiation zone was substantially extended in roots of seedlings grown at 28°C at all analyzed time points with differences increasing over time (Fig 3C).

To better understand potential reasons for these observations, we next counted and measured root cells along a longitudinal cell file from the quiescent center to the root–shoot junction in 5-day-old wild-type seedlings. While differences between temperatures in cell length seemed negligible across root zones (Figs 3D and EV3C), the differentiation zone between the end of the elongation zone and the root–shoot junction consisted of many more cells in high-temperature grown seedlings (Figs 3D and EV3D). When seedlings were grown for an additional 2 days, also moderate but significant differences in average cell length across the differentiation zone began to emerge (Fig EV3E). Hence, we did not observe any notable temperature-induced differences in (i) cell length throughout the root, and (ii) cell numbers in meristematic and elongation zones within the first 5 days of seedling development. We did, however, detect dramatic differences in the number of cells in the differentiation zone, suggesting that at elevated temperatures an increased rate of cell division in the root apical meristem causes the release of more cells to the elongation zone in a defined time period. Although temperature-sensitive cell elongation was also observed in older seedlings (Fig EV3E), we regard temperature-sensitive cell division as the driving force of temperature-induced root growth, representing a fundamental difference to cell elongation-driven shoot thermomorphogenesis. This again suggests that temperature sensing and signaling may involve different pathways in roots and shoots, which is supported by largely different transcriptome responses to elevated temperature in root versus shoot tissues we observed previously (Bellstaedt *et al*, 2019).

To test this hypothesis and to assess whether cell division rates increase at elevated ambient temperatures, we quantified the number of meristematic cells that are either entering the cell cycle (S-phase) or actively dividing (M-phase) in the root apical meristem. 5-ethynyl-2′-deoxyuridine (EdU), a thymidine analog, is widely used in DNA proliferation assays. EdU is incorporated into newly synthesized DNA and stains meristematic cells in the S-phase. When EdU-labeled cells divide, each daughter cell also contains EdU-labeled nuclei. Consequently, an increased number of labeled cells indicates a higher cell division rate. On the other hand, 4′,6-diamidino-2-phenylindole (DAPI) is a fluorescent stain that binds strongly to adenine-/thymine-rich regions in DNA allowing identification of cells in mitosis (M-phase). The experiments were conducted with 5-day-old seedlings, which is after the temperature response gates opened as shown above (Figs 1B and EV1A). Wild-type seedlings were grown at either constant 20°C or constant 28°C. Fig 3E shows that high temperature significantly increased the number of cells entering the cell cycle. Likewise, the number of cells actively dividing increased significantly at high temperature (Fig 3F). To seek genetic support for a substantial role of the cell cycle as a target of ambient temperature, we screened a number of cell cycle regulator mutants in their ability to respond to elevated temperatures with root growth promotion. We found that mutants defective in E2F regulators of cell cycle entry displayed a conditional temperature response phenotype (Fig EV3F). Together, these cell cycle marker and genetic data support the hypothesis stated above that higher temperature increases cell division rates.

Auxin transfers temperature information to the cell cycle

While these data indicated that the increase of cell division rates is an important driver of root growth at elevated temperatures, it remained unclear how temperature information is perceived in the root and how it reaches the cell cycle. A likely intermediate signal between a yet unknown root thermosensor and the cell cycle that has been shown to be involved in both root thermomorphogenesis (Hanzawa *et al*, 2013; Wang *et al*, 2016; Feraru *et al*, 2019; Gaillochet *et al*, 2020) and cell cycle regulation (reviewed in Perrot-Rechenmann, 2010; del Pozo & Manzano, 2014) is the phytohormone auxin.

The majority of auxin mutants that can be considered for temperature-responsive root growth are either already severely affected at control temperatures (which makes them in our opinion less informative) or showed rather mild phenotypes (e.g., Hanzawa *et al*, 2013; Martins *et al*, 2017; Feraru *et al*, 2019; Gaillochet *et al*, 2020). To overcome genetic redundancies and to assess the impact of several levels of auxin biology on temperature-induced root growth, we followed a pharmacological approach. To test the

© 2023 The Authors

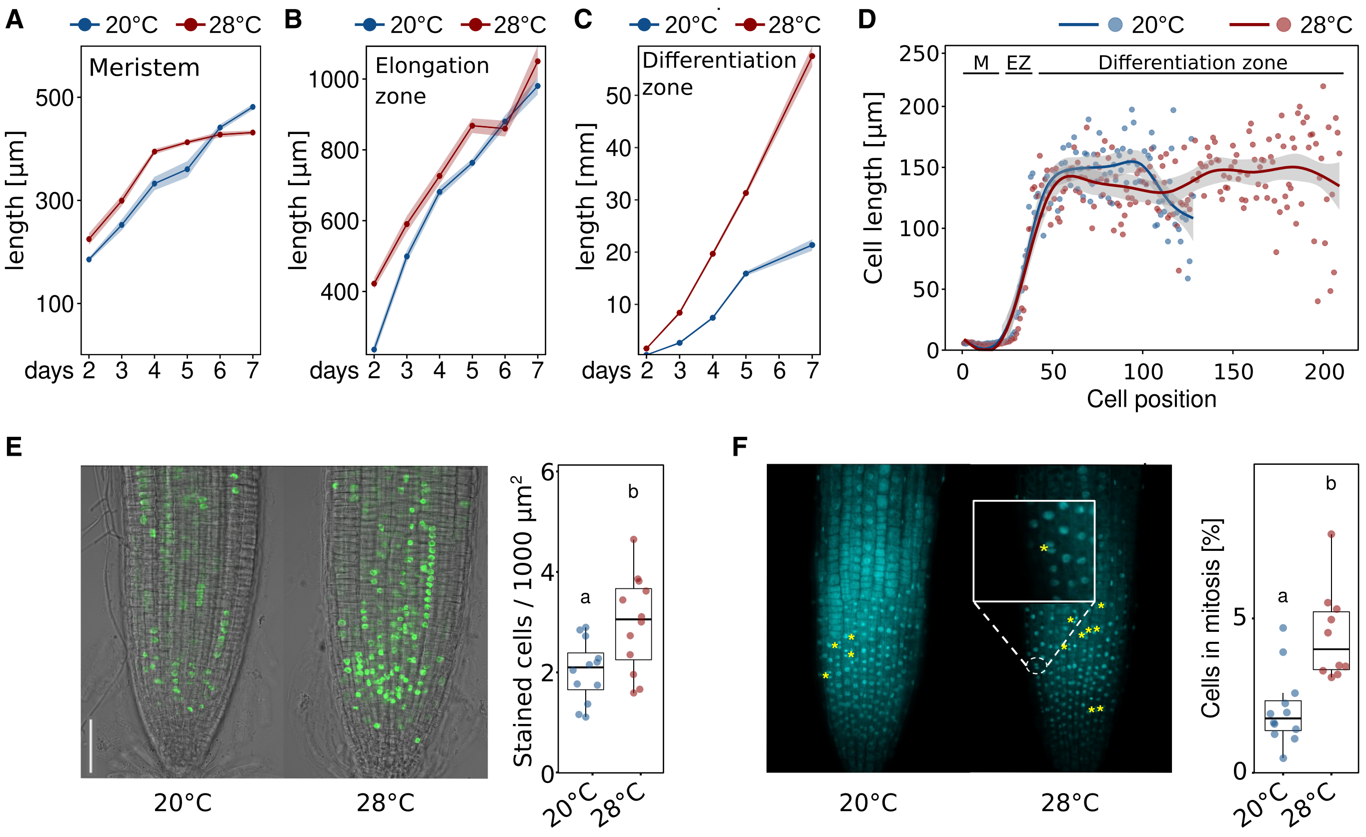

Figure 3. Temperature effects on root zones, cell size, cell number, and cell proliferation.

Total length of root zones between days 2 and 7 after sowing of Arabidopsis seedlings grown in LD at 20 or 28°C.

A–C (A) Meristem, (B) Elongation zone, and (C) Differentiation zone. (A–C) Solid lines and points show mean root zone lengths with half-transparent ribbons denoting SEM (n = 5–7 individual roots).

D Cell lengths in consecutive cortex cell files of 5-day-old seedlings starting from the root tip (quiescent center = position 1) spanning the meristem (M), elongation zone (EZ), and differentiation zone up to the root–shoot junction. Individual dots represent mean cell lengths (n = 8), and lines show a fitted smoothing function (generalized additive models) with the 95% confidence intervals shown in light gray ribbons.

E EdU staining marks cells that were in S-phase during the 1 h incubation time prior to microscopic imaging. Scale bar = 50 µm, n = 12.

F DAPI staining was used to identify cells currently in mitosis (examples are highlighted by yellow asterisks). Scale bar = 50 µm, n = 10–11. The data are representative of two (A–D) or three (E, F) independent experiments.

Source data are available online for this figure.

necessity of *de novo* auxin biosynthesis, we used a combined treatment of seedlings with the two IAA biosynthesis inhibitors kynurenine (He *et al*, 2011) and yucasin (Nishimura *et al*, 2014), which had previously been shown to effectively block temperature-induced hypocotyl elongation (Ibañez *et al*, 2018). Fig 4A shows that increasing concentrations of the two inhibitors gradually decreased the growth-promoting temperature effect also in the root. However, as inhibition of IAA biosynthesis affected root growth already at 20°C control conditions, we regard this only as indirect evidence because it is not strictly conditional. Direct evidence for an association of auxin biosynthesis with root thermomorphogenesis is provided by measurements of free IAA levels in root tips of 5-day-old seedlings grown at 20 or 28°C. Here, higher temperature increased auxin levels in the root tip (Fig 4B). A severe temperature-sensitive and conditional root growth phenotype in *yucQ* mutants that are defective in five root-expressed *YUCCA* genes (*yuc3,5,7,8,9*; Chen *et al*, 2014; Gaillochet *et al*, 2020) and mild phenotypes in the same root growth assay but strict dependency of temperature-induced *DR5NLS::GFP* reporter gene expression on *TRYPTOPHAN AMINOTRANSFERASE OF ARABIDOPSIS 1* (*TAA1/WEI8*) and *TRYPTOPHAN AMINOTRANSFERASE RELATED 1* (*TAR1*) (Figs EV4A and 4C) suggest that high temperature induces *de novo* auxin biosynthesis via the indole-3-pyruvate pathway.

To address the spatial origin of temperature-induced auxin biosynthesis, we next applied tissue strips soaked with the polar auxin transport inhibitor N-1-naphthylphthalamic acid (NPA) specifically to the root–shoot junction of seedlings grown at 20 or 28°C, respectively, to block auxin flow from the shoot to the root. At concentrations we previously found to inhibit the cotyledon-to-hypocotyl auxin flow, thereby inhibiting temperature-induced hypocotyl elongation when applied to the petioles (Fig EV4B; Bellstaedt *et al*, 2019), we find that application of NPA directly to the root–shoot junction had no effect on temperature-induced root elongation (Fig 4D). This provides another piece of evidence supporting temperature-sensitive and shoot-independent local auxin biosynthesis in the root.

 © 2023 The Authors

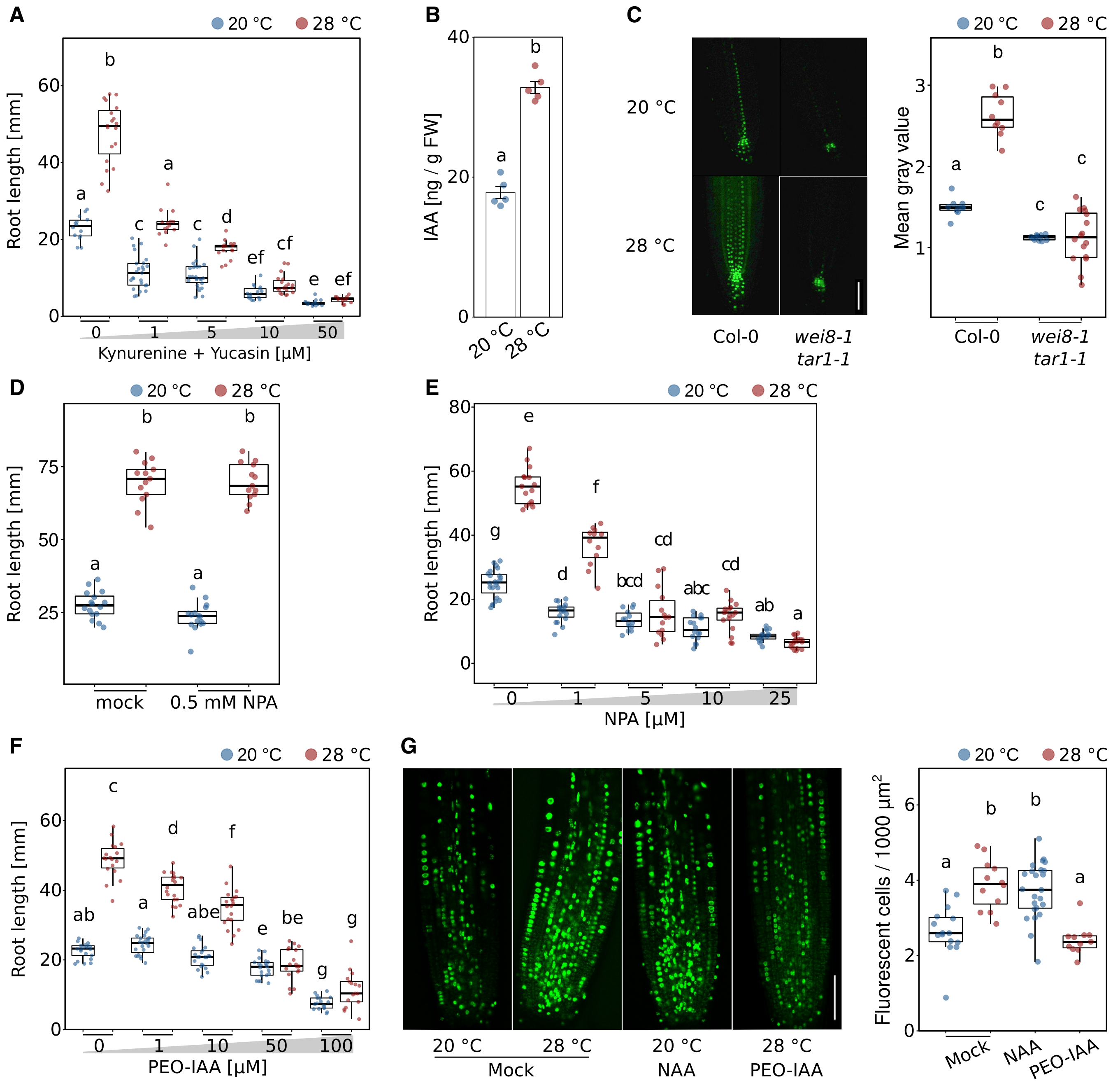

Figure 4. Root-derived auxin mediates temperature-induced root elongation.

A Root growth assay of seedlings grown for 7 days in the presence of increasing concentrations of auxin biosynthesis inhibitors kynurenine and yucasin (n = 14–26, both inhibitors always in equal concentrations) at the indicated temperatures.

B Free IAA levels of root tip samples of 5-day-old Col-0 (n = 5).

C Imaging and quantification of DR5NLS::GFP reporter activity in root tips of 5-day-old seedlings (scale bar = 50 µM, n = 10–16).

D Temperature-induced root growth is unaffected in 8-day-old seedlings if 0.5 mM NPA is applied to the root–shoot junction on day 5 (n = 13–16) at the indicated temperatures.

E Root growth assay of seedlings grown for 7 days in the presence of increasing concentrations of NPA (n = 12–23) at the indicated temperatures.

F Root growth assay of seedlings grown for 7 days in the presence of increasing concentrations of the auxin antagonist PEO-IAA (n = 17–23) at the indicated temperatures.

G (Co-)incubation of 5-day-old seedlings for 3 h with 10 µM EdU only or in combination with either 100 nM NAA or 50 µM PEO-IAA (n = 11–24), scale bar = 50 µm.

(A–G) Boxplots show medians, interquartile ranges, and min–max values (A, C–E). Barplot shows mean values and error bars show SEM (B). Individual data points are superimposed as colored dots. Different letters denote statistical differences at $P < 0.05$ as assessed by one-way (B, G) or two-way (A, C–F) ANOVA and Tukey's HSD *post hoc* test. The data are representative of two (A–C, E, F) or three (D, G) independent experiments.

Source data are available online for this figure.

© 2023 The Authors

To assess the general impact of polar auxin transport, we conducted the same dose–response assay as described above for auxin biosynthesis inhibitors, using NPA applied to the whole media. We observed a similar picture with gradually decreasing temperature responsiveness on increasing concentrations of NPA (Fig 4E). Also here, small concentrations of the inhibitor already affected root growth at both temperatures.

Lastly, we blocked auxin signaling by applying the auxin antagonist α-(phenylethyl-2-one)-IAA (PEO-IAA), which competes with native free IAA for auxin co-receptor binding (Nishimura *et al*, 2009). While root elongation was not affected by PEO-IAA concentrations up to 10 μM at 20°C, increasing concentrations of the inhibitor within the same range gradually and significantly decreased root growth at 28°C (Fig 4F), providing a strictly conditional phenotype. Above 10 μM PEO-IAA, seedlings grown in both temperatures were affected, albeit root lengths of seedlings grown at 28°C decreased more severely. Together, these data support an essential rather than a modulating role of auxin in temperature-induced root elongation as proposed previously.

Auxin controls G1/S-phase transition of cells entering the cell cycle (Perrot-Rechenmann, 2010; del Pozo & Manzano, 2014). To assess a direct link among temperature, auxin, and cell division rates, we next asked whether exogenous addition or inhibition of auxin would influence the number of meristematic cells entering the cell cycle. We, therefore, performed an EdU staining assay in the presence of the synthetic auxin naphthalene-1-acetic acid (NAA) or the auxin antagonist PEO-IAA. We found that the S-phase-promoting effect of temperature alone (28°C, see also Fig 3E) could be mimicked by adding NAA (100 nM) to the 20°C samples (Fig 4G). Vice versa, the temperature-mediated increase in the number of cells entering the S-phase could be counteracted by adding PEO-IAA (50 μM) to the 28°C samples (Fig 4G).

Collectively, these data indicate that auxin relays temperature information from a yet unknown thermosensor to the root apical meristem where it promotes cell proliferation at elevated ambient temperatures.

Temperature regulates PIN protein expression patterns in the root tip

To investigate how the temperature-induced increase of auxin levels is maintained in root apical meristems, we examined the role of polar auxin transport in more detail. As PIN-FORMED (PIN) auxin efflux transporters regulate large parts of the auxin flow through the root, we tested several *pin* mutants for their behavior in root elongation assays. We found that while *pin1-1* and *eir1-1* (a *pin2* allele) mutant roots were similar to wild type at 20°C control conditions, *pin1-1* did not respond at all to high temperatures, and *eir1-1* showed a significantly reduced temperature response (Fig 5A), the latter confirming a previous report (Hanzawa *et al*, 2013). A mutant allele of *PIN3* (*pin3-4*) behaved like wild type in both temperatures, suggesting that it is not required. In contrast, *pin4-2* mutants, which were likewise not significantly different from the wild type at 20°C, hyperelongated at 28°C (Fig 5A). As such, PIN1 and PIN2 seem to act as positive regulators of temperature-induced root growth, while PIN4 inhibits excessive root growth at elevated temperatures. Importantly, these phenotypes were conditional, suggesting that these are genuine temperature effects. To further inspect these phenotypes, we used propidium iodide staining and confocal microscopy to count the number of meristematic cells in those mutants with defects in temperature-induced root elongation growth, namely *pin1-1*, *eir1-1*, and *pin4-2*. Along a longitudinal cell file, *pin1-1* showed a tendency for fewer meristematic cells, but these differences were not statistically significant (Fig EV5A and B). Likewise, the *eir1-1* mutant did not differ from the wild type. In contrast, we observed an increased meristem cell number in *pin4-2* at 28°C compared to that of the wild type (Fig EV5A and B), which may explain its long root phenotype at 28°C. When we assessed the activity of fluorescent DR5 reporter constructs in *pin* loss-of-function backgrounds in response to temperature, we found no response in *pin1-1* and increased DR5 activity in *pin4-2* at both temperatures (*eir1-1* lines with DR5 reporters were not available; Fig 5B). These observations align with the root growth defects shown in Fig 5A. Furthermore, we found that both *PIN1* and *PIN2* are essential for temperature-induced increase of cell division rates as shown by EdU staining of root apical meristems in *pin1-1* and *eir1-1* seedlings (Fig 5C), further substantiating the proposed function of auxin in thermo-responsive regulation of the cell cycle.

Although NPA application to the root–shoot junction already suggested independence of shoot-derived auxin (Fig 4D), we selected one short root *pin* mutant (*pin1-1*) and one long root *pin* mutant (*pin4-2*) and asked whether their temperature-induced root growth phenotypes (Fig 5A) originate in the root or may possibly be caused by long-distance transport of shoot-derived auxin requiring PIN function. Micrografting showed that the observed phenotypes did only occur when *pin* mutants were used as rootstocks (Fig EV5C and D), further strengthening that the elevated auxin levels at high temperatures in the root tip are root derived.

To better understand how temperature potentially affects PIN proteins, we first assessed whether *PIN*s may be transcriptionally regulated by temperature. Quantitative RT–PCR of *PIN1-4* in whole roots or root tips of 5-day-old seedlings grown at 20 versus 28°C displayed little temperature responsiveness (Fig 5D). Although we did observe a slight decrease in *PIN1* expression levels in root tips at high temperature (Fig 5D), overall transcriptional regulation of *PIN*s seems to be hardly affected by temperature changes. We then looked at the behavior of GFP fusion proteins of PIN1, PIN2, and PIN4. PIN-GFP fusion proteins responded in parts strongly to temperature changes. When 5-day-old seedlings were shifted from 20 to 28°C for 4 h, GFP fusion protein signals of PIN1 and PIN2 in the root tip virtually disappeared, while PIN4 reporter levels remained unaffected (Fig EV5E–G). However, these PIN1 and PIN2 effects were transient, as illustrated by unchanged GFP signal intensities in seedlings constantly grown at the respective temperatures (Fig EV5E and F). It is, therefore, unclear whether this short-term disappearance affects root growth at all. PIN4-GFP levels in the columella showed a tendency to decrease at constant 28°C (Fig EV5G), but the differences were not statistically significant. As polar localization of PINs controls the direction of auxin flow, we also quantified ratios of basal (lower)/apical (upper)-to-lateral PIN-GFP ratios in response to temperature. For PIN2-GFP, we observed an increased basal-to-lateral ratio in cortex cells of seedling roots grown at constant 28°C compared to constant 20°C (Fig 5E). A shift of cortical PIN2 to the basal membrane would cause auxin to

 © 2023 The Authors

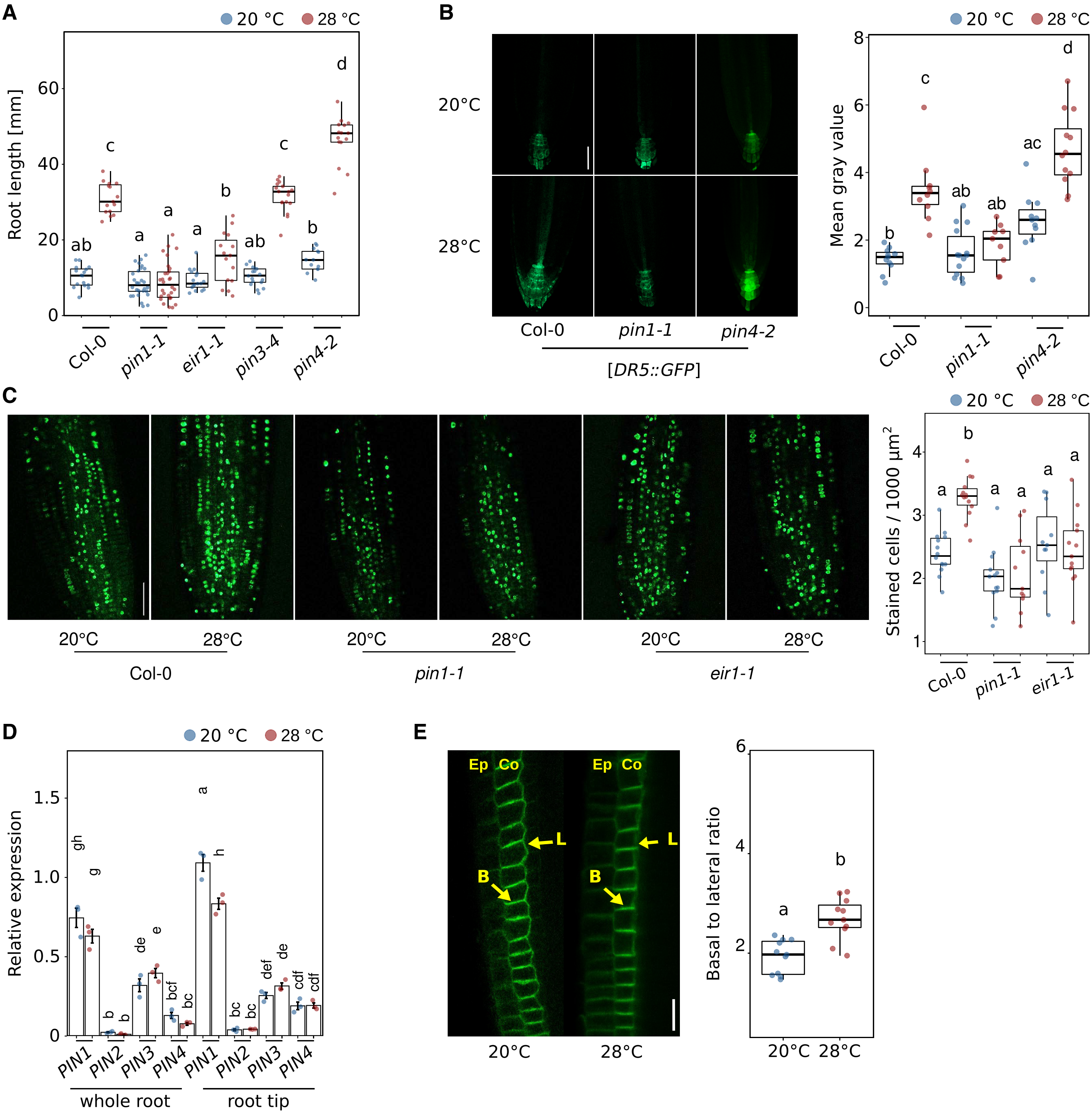

Figure 5. Polar auxin transport is required for temperature-induced elongation of the root.

A Root growth assay of 7-day-old seedlings grown at the indicated temperatures (n = 13–30).

B Imaging and quantification of *DR5::GFP* reporter activity in root tips of 5-day-old seedlings (scale bar = 50 µm, n = 9–14).

C EdU staining of 5-day-old seedlings at indicated temperatures (n = 11–14).

D qRT–PCR expression analyses of *PIN1-4* from whole root and root tip samples, respectively, Col-0 RNA was extracted with respective organs and first-strand cDNA was then synthesized (n = 3).

E PIN2-GFP relocalization patterns of root meristem of 5-day-old seedlings grown at the indicated temperatures. Ep = epidermis, Co = cortex, yellow arrows mark the localizations of lateral or basal membranes in a cell (n = 12–13), scale bar = 20 µm (n = 10–11). (A–E) Boxplots show medians, interquartile ranges, and min–max values with individual data points superimposed as colored dots. Different letters denote statistical differences at $P < 0.05$ as assessed by two-way ANOVA and Tukey's HSD *post hoc* test. The data are representative of two (B, D), three (E), or four (A, C) independent experiments.

Source data are available online for this figure.

© 2023 The Authors

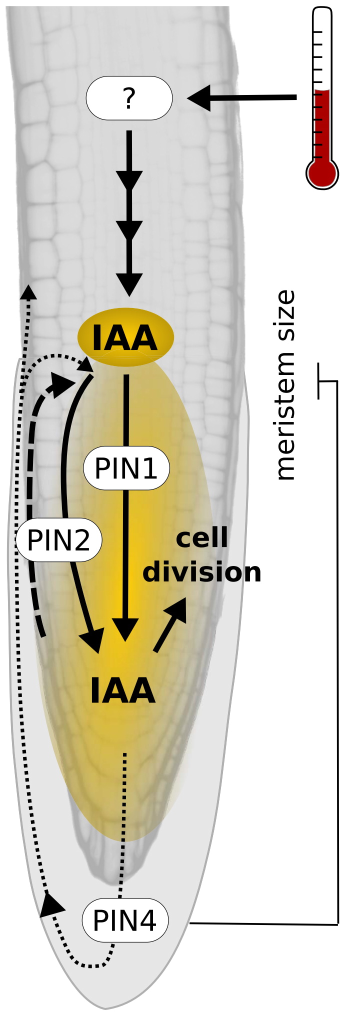

Figure 6. Schematic model of the major regulatory processes in the root apical meristem during root thermomorphogenesis.

Elevated temperature sensed by a yet unknown thermosensor induces auxin biosynthesis, resulting in elevated auxin levels in the root tip. Polar auxin transporters PIN1, PIN2, and PIN4 help to maintain an auxin maximum in the root apical meristem, which increases cell division rates, driving primary root elongation. In addition, functional PIN4 restricts meristem size at elevated temperature, possibly preventing excessive root growth.

be preferably transported downward into the root tip, consistent with the increase of IAA levels (Fig 4B). A parallel shift of epidermal PIN2 to the apical membrane, as shown by Hanzawa *et al* (2013), would promote the auxin flow back upwards in line with the reverse fountain model.

Collectively, the reported results indicate that elevated temperature is perceived by a yet unknown root thermosensor, which either directly or indirectly activates local *de novo* auxin biosynthesis via the indole-3-pyruvate pathway and a PIN-dependent increased flow of auxin through the root tip, resulting in an auxin maximum. This likely causes an acceleration of cell division rates in the root apical meristem potentially via auxin-regulated E2F transcription factors, which ultimately results in increased primary root elongation at elevated temperatures (Fig 6).

Discussion

Research efforts in the past 15 years have generated a reasonable understanding of how shoot organs sense and respond to elevated ambient temperatures (reviewed in Quint *et al*, 2016; Delker *et al*, 2017; Casal & Balasubramanian, 2019). And although we are still lacking data from natural habitats, there is also some ecophysiological insight into the advantages of such thermomorphogenic behavior, which seems to foster evaporative cooling (Crawford *et al*, 2012; Park *et al*, 2019) and thereby photosynthetic efficiency in *A. thaliana* and also in non-vascular plants like the liverwort *Marchantia polymorpha* (Ludwig *et al*, 2021). Understanding of thermomorphogenic root growth, however, is literally still in the dark. Just like above-ground organs, root systems are exposed to a range of different (soil) temperatures, affecting the uptake and transport of water and nutrients, root growth, and development (Koevoets *et al*, 2016; de Lima *et al*, 2021). Noteworthy, roots of some species are even able to actively adjust their growth in a directional manner towards or away from a temperature source in a positive or negative thermotropic manner, respectively (van Zanten *et al*, 2021). The aim of this study was therefore to pick up existing knowledge about different aspects of root responses to elevated ambient temperature and conduct experiments necessary to connect the existing fragmentary understanding to derive a comprehensive model of the physiological mechanism(s) regulating root thermomorphogenesis.

While it is established that shoot organs can sense and respond to temperature cues, it was unclear whether the root has the same capabilities. This is, however, an important question to answer because it affects the search for root thermosensors and signaling pathway(s). Several possibilities exist: (i) roots may be unable to sense temperature themselves and completely depend on shoot-derived long-distance signaling of temperature information. In contrast, (ii) they may constitute an autonomous system that is able to sense and respond to temperature cues independent of the shoot, or (iii) they may to some degree integrate above-ground temperature information in their below-ground growth “decision” process. In support of the significant influence of shoot-derived signals on root thermomorphogenesis, Gaillochet *et al* (2020) have proposed that a shoot module consisting of HY5, which is known to coordinate above-ground with below-ground growth (Chen *et al*, 2016; van Gelderen *et al*, 2018), together with phytochromes and PIFs exerts a central function in coupling growth responses between shoot and root in a temperature context. The authors suggested that a PHY-PIF-HY5 module may activate auxin to regulate root growth. However, the ability of detached wild-type roots to elongate more at 28°C than at 20°C (Bellstaedt *et al*, 2019; Fig 2A) in combination with the absence of a growth-inhibiting effect of *pifQ*, *pif4-2*, or *hy5-51* mutant shoots grafted onto wild-type rootstocks (Figs 2B–D and EV2B) favors a scenario in which roots are to be regarded as autonomous systems that can independently sense and respond to temperature cues. This does not rule out the presence of temperature-sensitive shoot-to-root communication, possibly involving PHYB (Fig EV2C), but renders it non-essential for temperature-induced root elongation. Furthermore, Belén Borniego *et al* (2022) recently demonstrated that neither PHYB nor the other two known shoot thermosensors ELF3 (Jung *et al*, 2020) and PIF7 (Chung *et al*, 2020) fulfill thermosensory function in the root. Although intact seedlings

 © 2023 The Authors

of additional selected shoot thermomorphogenesis mutants did show weak root growth defects, all tested lines were able to respond to high temperature (Fig EV2D; Yang *et al*, 2017; Gaillochet *et al*, 2020). We, therefore, conclude that, although being expressed in the root, with a possible exception of HY5 (Gaillochet *et al*, 2020; Lee *et al*, 2021), the major regulators of shoot thermomorphogenesis most likely play a rather secondary role in root thermomorphogenesis, and primary regulators yet wait to be identified. Together, this suggests that shoot and root thermomorphogenesis are regulated by different signaling pathways.

To get a handle on such a pathway, it helps to understand which cellular process is mediating temperature-induced growth of the primary root. In juvenile shoots, the primary cellular process promoting hypocotyl and petiole growth at elevated temperatures is cell elongation. We found that roots of seedlings grown at elevated temperatures generate substantially more cells than those grown at lower temperatures (Fig 3D). This temperature-sensitive increase in cell numbers seemed restricted to differentiating and mature cells (Fig EV3D), while meristematic and elongating cells were hardly affected. Several studies from other species likewise observed a temperature-invariant meristem size, for example, in wheat (Burstrom, 1953), onion (Carmona & Cuadrado, 1986), and maize (Silk, 1992). For *A. thaliana*, different effects of elevated temperature (< 30°C) on meristem size have been reported, ranging from shorter root apical meristems (Yang *et al*, 2017; Feraru *et al*, 2019) to even larger areas with cell division activity (Hanzawa *et al*, 2013) in response to elevated temperature. We found that meristem size slightly varies with temperature over time, but differences were minor (Figs 3A and EV3C). Inconsistencies between studies may be explained by meristem size being a rather sensitive trait, by the age of the seedlings at the time of measurement, or alternatively by the manner of defining the meristem, which ranged from monitoring actively dividing cells (Yang *et al*, 2017) via reporter gene expression domains (Hanzawa *et al*, 2013) to cell size features (Feraru *et al*, 2019; this study). The same inconsistencies apply to the description of the effect of elevated temperature on cell length between studies. To understand the timing of cellular temperature responses, it makes sense to focus on the temporal dynamics of cell number and length in the differentiation zone, which reflects the outcome of potential changes in meristematic and elongation zones, and practically covers (almost) the whole root. Here, we observed a drastic increase in cell numbers as early as 3 days after sowing (Fig EV3D). However, this is still within the first 4 days during which a temperature-sensitive growth response was absent at the level of whole root length. Only after the 4-day-long gating period (Figs 1B and EV1A), average cell length in the differentiation zone slightly increased in seedlings grown at high temperature. This shows that (i) cell division responses precede cell elongation responses, and (ii) cell division responses are more pronounced than cell elongation responses. Together, this suggests that elevated temperature promotes both cell division rates and cell elongation with the former having a greater impact on ultimate root length during the time period investigated in this study. Furthermore, as increased cell division rates precede the observable differences in whole root length, a gating period probably does not exist on the cellular level. Hence, the predominant factor responsible for exaggerated root elongation at elevated temperatures is most likely an increase in cell division rates in the root apical meristem. Obviously, this is a fundamental mechanistic difference in the manner in which high-temperature cues are translated to growth responses between shoot and root tissues, respectively.

Quantification of cells at different stages of the cell cycle supports this conclusion. While ambient temperatures well above 30°C seem to inevitably result in severe reduction of cell division in the root apical meristem (González-García *et al*, 2022), we observed that at 28°C more cells entered the cell cycle (Fig 3E), and likewise more cells were actively dividing (Fig 3F) in comparison to plants grown at 20°C. These findings are complementing a wealth of data from the last 60+ years that describe acceleration of the cell cycle and increased cell division rates caused by elevated temperatures across species (e.g., Erickson, 1959; Murin, 1966, 1981; Silk *et al*, 1989; Silk, 1992; Grif *et al*, 2002; Hanzawa *et al*, 2013; Yang *et al*, 2017). Interestingly, a similar mechanism has recently been shown to regulate thermo-responsive leaf growth (Saini *et al*, 2022). Compared to cell elongation, cell division involves further investment into cell material, it may thus be more costly in terms of biomass. But a higher number of cells also implies more cell walls that are supporting the rigidity of the structure. While this investment may not make sense for shoot tissues of annual plants that face comparably little resistance in their aerial environment, more cell walls increase physical strength (e.g., force to fracture) and therefore confer higher resistance to increased soil pressure. This is an asset when roots explore deeper soil layers.

To comprehend how elevated temperatures promote primary root elongation, we need to understand how temperature information connects to the cell cycle. One such connector promised to be auxin, which (i) also acts as a messenger between thermo-sensing and response in shoot thermomorphogenesis (Bellstaedt *et al*, 2019), and (ii) is known to promote the entrance of cells into the cell cycle especially by activating G1-S-phase genes (Perrot-Rechenmann, 2010; del Pozo & Manzano, 2014). While several groups had previously shown that auxin plays an important role in root thermomorphogenesis (Hanzawa *et al*, 2013; Wang *et al*, 2016; Feraru *et al*, 2019; Gaillochet *et al*, 2020), its mechanistic role remained rather vague. Our data now support a model where auxin connects a temperature signal with the cell cycle in the root apical meristem. At high temperatures, free IAA levels are significantly increased in the root tip (Fig 4B) which contains the root apical meristem. In contrast, Gaillochet *et al* (2020) found no difference in free IAA levels between temperatures in seedling roots and suggested that an increase in auxin levels is not required for root thermomorphogenesis. However, this does not necessarily contradict our data because Gaillochet *et al* (2020) sampled whole roots, which may have diluted a spatially restricted temperature-sensitive increase of auxin in root tips. Grafting (Figs 2B–D and EV2A and B), prevention of shoot–root auxin flow (Fig 4D), and dependency of *DR5NLS::GFP* reporter gene activity on rate-limiting enzymes of the indole-3-pyruvate pathway (Fig 4C) suggest that high temperature induces local *de novo* auxin biosynthesis via the YUC/TAA route. The newly synthesized IAA is unlikely to originate from the shoot as blocking of auxin flow from the shoot to the root by localized application of NPA does not affect temperature responses in the root (Fig 4D). Instead, the IAA may be synthesized in the root apical meristem itself or elsewhere in the root and then transported downwards. Importantly, the temperature-induced increase of cell division rates depends on auxin (Fig 4G), providing a direct link between temperature, the phytohormone, and the cell cycle.

© 2023 The Authors

Conditional root growth phenotypes of several *pin* loss-of-function mutants (Fig 5A) and the dependence of increased DR5 signal on PINs in elevated temperatures (Fig 5B) suggest the involvement of polar auxin transport in the generation and/or maintenance of an auxin maximum in the root tip, which is supported by the expression pattern of, for example, PIN2 protein. For PIN2-GFP, we observed a quantitative shift at 28°C from the lateral to the basal (lower) membrane of cortex cells. While this differs from Sauer *et al* (2006) who observed the opposite upon exogenous application of auxin to seedling roots in a non-temperature context, it complements findings from Hanzawa *et al* (2013) who described a lateral-to-apical (upper) shift of PIN2-GFP in epidermal root cells of high-temperature-treated seedlings. Together, this suggests an increased auxin flow through the root apical meristem at elevated ambient temperatures. Under the reverse fountain model (Benková *et al*, 2003; Grieneisen *et al*, 2007), redistribution of auxin in the columella requires PIN4, which also functions in generating a local sink and thereby an auxin gradient in the root tip (Friml *et al*, 2002). We observed a tendency of decreased PIN4-GFP levels in the columella at 28°C (not significant; Fig EV5G), possibly interrupting this auxin flow and instead trapping auxin in the root tip, contributing to the maximum we detected in root tips of plants grown at high temperature (Fig 4B). Arguably, this would counteract an increased auxin flow through the meristem. However, *pin4-2* mutants displayed hyperelongated roots at elevated temperature (Fig 5A), making PIN4 a negative regulator of this response, suggesting that in a temperature context, PIN4's role in generating an auxin maximum in the root tip is likely secondary to its role in restricting meristem size (Fig EV5A and B). Although its localization patterns seemed unresponsive to temperature and PIN1-GFP levels were only transiently affected by high temperature (Fig EV5E), PIN1 must be essential for this process, as its loss in *pin1-1* mutants completely abrogated temperature-induced root elongation (Fig 5A).

As a result of elevated IAA levels in the meristematic region, cell division rates are increased. This process is likely reversible as shown by the effects of exogenously added auxin or auxin antagonist on the number of S-phase cells in the root apical meristem (Fig 4G). Interestingly, Zhu *et al* (2015) demonstrated that low temperature reduces both meristem size and cell number, repressing the division potential of meristematic cells by reducing auxin accumulation, possibly through the repressed expression of PIN1/3/7 and auxin biosynthesis-related genes. This suggests that temperature may target the same cellular process, cell division, by the same players, auxin and PINs, in an either repressive (low temperature) or promoting (high temperature) manner.

Concluding remarks

In this study, we aimed to identify the missing links between published information on specific aspects of root thermomorphogenesis by conducting experiments that cover the existing gaps. Based on this, we propose a comprehensive model for a molecular mechanism regulating root thermomorphogenesis from temperature sensing via transduction of the temperature signal to a cellular process that can explain the promoting effect of elevated ambient temperatures on root growth to a large degree. We favor a model in which roots can autonomously sense and respond to elevated ambient temperatures (Fig 6). In elevated temperatures, one or several yet unknown root thermosensor(s) activate(s) *de novo* local auxin biosynthesis via the indole-3-pyruvate pathway, possibly within or close to the root apical meristem. The generated auxin maximum in the root tip is maintained in a PIN-dependent manner and promotes the entry of cells into the cell cycle, causing an increase in cell division rates. As such, auxin functions as a gas pedal. However, the role of PIN4 seems more complex than suggested in this simple model. We find that a likely reason for the hypersensitive root elongation response of *pin4-2* mutants in high temperature is an increase in meristem size. To use the same analogy as above, at elevated temperatures, functional PIN4 might not only redistribute auxin in the columella but also act as a brake pedal, restricting meristem size and thereby preventing hyperelongation of the root by limiting the extension of the meristematic zone.

Furthermore, we do not know anything about the nature of thermosensors in the root. In case thermo-sensing occurs in growing tissues of the roots, it would ensure short distances from the location of thermo-sensing to the major growth-promoting cellular process, cell division, in the root apical meristem. Such a scenario would allow the primary root to determine its thermomorphogenic behavior primarily on basis of the deepest soil layers it has already penetrated. While this seems rather one dimensional, it may be a highly versatile system. Provided that the same mechanism is active in lateral roots, it would allow primary root and lateral roots to respond to temperature independently of each other. Root plasticity in different soil temperatures may then be based on semi-independent root modules or phytomers. Alternatively, multiple thermosensors may be acting in different regions of the root, which would require to integrate more complex temperature information across various soil zones. If so, the root would be in need of a long-distance signal transporting temperature information from various regions in the root to the root apical meristem. Auxin would be a natural candidate for this type of mobile signal.

More detailed analyses are needed to identify the cell cycle components regulated by auxin, and to understand how other hormones cross-feed information into this network. In any case, to understand root thermomorphogenesis on a mechanistic level, we might have to refrain from trying to find parallels to shoot thermo-signaling pathways, which ultimately modulate cell elongation. Instead, it seems important to understand how temperature information influences the cell cycle in the root apical meristem.

Materials and Methods

Plant material and growth conditions

Seeds of *A. thaliana* and all other species were surface sterilized, rinsed with sterile water, and then imbibed and stratified for 3 days at 4°C in deionized water before sowing. *A. thaliana* genotypes used in this study have been described previously or were obtained from the Nottingham Arabidopsis Stock Centre (NASC; http://arabidopsis.info): *axr5-1* (Yang *et al*, 2004), *tir1-1 afb2-3* (Parry *et al*, 2009), *wei8-1 tar1-1* (Stepanova *et al*, 2008), *yucQ* (Chen *et al*, 2014), *cop1-6* (McNellis *et al*, 1994), *elf3-1* (N3787; Hicks *et al*, 1996), *hy5-51* (N596651; Alonso *et al*, 2003), *pif4-2* (N66043, Leivar

 © 2023 The Authors

et al, 2008), *pifQ* (N66049, Leivar *et al*, 2008), *35S::PIF4-HA* (Nozue *et al*, 2007), *phyABCDE* (Hu *et al*, 2013), *YHB* (P35S:AtPHYBY276H in phyA-201 phyB-5; Su & Lagarias, 2007), *phyB-1* (Reed *et al*, 1994), *e2fa-2* (Berckmans *et al*, 2011), *e2fb* (Berckmans *et al*, 2011), *e2fab* (Yao *et al*, 2018), *pin1-1* (Okada *et al*, 1991), *eir1-1* (Luschnig *et al*, 1998), *pin3-4* (Friml *et al*, 2003), *pin4-2* (Friml *et al*, 2002), *DR5revp:GFP* (Friml *et al*, 2003), *DR5revp:SV40:3×GFP* (*DR5NLS::GFP*; Weijers *et al*, 2006), *PIN1::PIN1-GFP* (Benková *et al*, 2003), *PIN2::PIN2-GFP* (Luschnig *et al*, 1998; Müller *et al*, 1998), *PIN3::PIN3-GFP* (Žádníková *et al*, 2010), and *PIN4::PIN4-GFP* (Blilou *et al*, 2005). Wild-type strains were Col-0 (N19992) and Ler-0 (NW20). Seeds from other species are designated as follows: *Solanum lycopersicum* (cv West Virginia 106) and *Brassica oleracea* (cv collard, NASC ID N29002). Unless stated otherwise, seedlings were grown on solid *Arabidopsis thaliana* solution (ATS, Lincoln *et al*, 1990) nutrient medium including 1% (w/v) sucrose on vertically oriented plates under long-day conditions (16 h of light/8 h of dark) with 90 µmol m^{-2} s^{-1} photosynthetically active radiation (PAR) from white fluorescent lamps (T5 4000K).

Root growth assays

Seedlings were placed on ATS medium, grown at 20 or 28°C, and primary root length was determined 7 days after sowing (unless stated otherwise). Where indicated, plates were supplemented with various concentrations of auxin inhibitors kynurenine (He *et al*, 2011) and yucasin (Nishimura *et al*, 2014), NPA (Scanlon, 2003), or PEO-IAA (Hayashi *et al*, 2012). For combined treatments with kynurenine and yucasin, both compounds were applied in equal concentrations. All measurements were based on digital photographs of plates using RootDetection (www.labutils.de) and depict the total length of the root.

For detached root assays, 4-day-old Arabidopsis Col-0 and *B. oleracea* seedlings, as well as 5-day-old *S. lycopersicum* seedlings grown at 20°C were dissected at the root–shoot junction to obtain roots only. The detached roots were then grown on vertically oriented ATS plates at either 20 or 28°C for another 4 days.

NPA treatment assays

Thin tissue strips were soaked in lukewarm ATS medium with or without the addition of 0.5 mM NPA (Duchefa) and carefully placed across petioles/cotyledons or the root–shoot junction. For petiole/cotyledon application, seedlings were pre-grown at 20°C for 5 days, and then NPA strips were applied for an additional 3 days at either 20 or 28°C, respectively. For application to the root–shoot junction, seedings were grown at either constant 20°C or constant 28°C for 8 days. NPA strips were applied on day 5.

Infrared imaging of root and hypocotyl growth

Vertically oriented ATS plates (with 2-day-old seedlings grown at 20 or 28°C) were put perpendicular to the camera (Panasonic G5 with hot mirror filter replaced by an IR filter, enabling only IR light to reach the sensor; www.irrecams.de). To monitor growth dynamics, pictures were automatically taken every hour (root) or every 2 h (hypocotyl). Derived root or hypocotyl lengths were used to calculate growth rates.

Hypocotyl micrografting

Grafting was performed on 9-day-old seedlings and carried out according to previously published protocols (Melnyk, 2017) and as described in Serivichyaswat *et al* (2022). In brief, seeds were sown on ATS medium at 4°C darkness, stratified for 2 days at 4°C, and then shifted to 20°C in a growth cabinet for another 7 days under long-day photoperiods (16 h of light/8 h of dark) with 90 µmol m^{-2} s^{-1} white light (T5 4000K). Next, seedlings were grafted and recovered for 7 days on a water-mounted filter paper/membrane. Successfully recovered grafted plants were selected, transferred to new ATS medium, and cultivated at 20 or 28°C, respectively, under the same conditions described above for another 7 days. Root growth differences were then determined by measuring the root growth between days 16 and 23. The micrografting procedure is detailed in Fig EV2A.

Measurement of cell length and cell number

Root cell measurements were conducted by staining seedlings with Calcofluor White (Merck, 18909-100ML-F) or propidium iodide (Merck, P4170). Briefly, seedlings were fixed in pure ethanol for 2 h to overnight, washed twice with 1× PBS, followed by a permeabilization step using 3% Triton X-100 + 10% DMSO in 1× PBS for 30 min to 1 h. Next, 0.1% Calcofluor White or 0.1 mg ml^{-1} propidium iodide in 1× PBS was freshly prepared and the seedlings were stained for 30 min. Subsequently, seedlings were washed twice in 1× PBS with gentle shaking. To image Calcofluor White, we used 405 nm excitation and detected signals at 425–475 nm. For imaging of propidium iodide, we used 488 nm excitation and detected signals at 600–637 nm. All measurements were performed on all individual cells of a consecutive cortex cell file using the ZEN 3.1 software (Zeiss) for 8–12 independent seedlings per experiment. The meristematic zone was defined as the zone between the quiescent center and the last cell that did not yet double its size in comparison with the previous cell. The elongation zone followed the meristematic zone and was defined as the zone from first cell with double the size of the previous cell to the last cell before root hair bulges became visible. The following differentiation and maturation zone was defined as the zone from first cell below the first trichoblast bulge to the root–shoot junction.

EdU staining

5-Ethynyl-2′-deoxyuridine (EdU) staining was performed with the EdU Click-488 Imaging Kit (Carl-Roth) according to the manufacturer's instructions. Briefly, 5-day-old Col-0 seedlings (at ZT1, 1 h after lights on) were immersed for 1 h in liquid ATS medium containing 10 µM EdU, and fixed in 4% (w/v) paraformaldehyde and 0.5% Triton X-100 for 20 min. After washing twice with 1× PBS, samples were incubated in the reaction cocktail for 30 min in the dark. The reaction cocktail was then removed, and samples were washed with 1× PBS, followed by confocal microscopy with a Zeiss LSM 780 AxioObserver (excitation wavelength: 488 nm; emission wavelength: 491–585 nm). The region of interest (root meristem) was determined with the same fixed area in all measurements, and positively stained cells were counted in this area to calculate cells per 1,000 µm^2.

© 2023 The Authors

When stated, seedlings were co-incubated with the auxin antagonist PEO-IAA or the synthetic auxin NAA (Duchefa). Here, seedlings were stained with EdU (10 μM) + PEO-IAA (50 μM) in liquid ATS for 3 h, or EdU (10 μM) + NAA (100 nM) in liquid ATS for 3 h (DSMO as mock) prior to fixation as described above.

DAPI staining

At ZT2-ZT3 (2–3 h after lights on), 5-day-old Col-0 seedlings were fixed with pure ethanol for 2 h, rinsed twice with 1× PBS, followed by a permeabilization step using 3% Triton X-100 + 10% DMSO in 1× PBS for 30 min. Seedlings were subsequently washed three times with 1× PBS, and then stained with 100 μg ml^{-1} 4′,6-diamidino-2-phenylindole (DAPI) in the dark for 15 min at room temperature, and processed under a Zeiss LSM 780 AxioObserver (excitation wavelength: 405 nm; emission wavelength: 425–508 nm). The region of interest (root meristem) was determined, and all stained nuclei in this area were counted by using the ImageJ software. The mitosis ratio was determined by counting cells in mitosis (condensed chromosomes visible) divided by all nuclei in this area.

Auxin reporter assay

Five-day-old Col-0, *pin1-1*, *pin4-2*, and/or *wei8-1 tar1-1* seedlings carrying DR5revp::SV40:3×GFP (DR5NLS::GFP) reporters were fixed directly with 4% (w/v) paraformaldehyde at room temperature, washed with 1× PBS, and kept in the dark until imaging (excitation wavelength: 561 nm; emission wavelength: 571–615 nm). Columella cells including the quiescent center were determined as the fixed area through all measurements. Mean gray values were measured by using ImageJ.

IAA analytics

Col-0 seedlings were grown for 5 days as described above at 20 or 28°C. On day 5, root tips were harvested and immediately homogenized in liquid nitrogen. Extraction of indole 3-acetic acid (IAA) was done using 50 mg homogenized material, and three rounds of extraction with 400, 200, or 100 μl of 80% methanol were acidified to pH 2.4 with hydrochloric acid. In order to enhance cell rupture and extraction, one steel bead of 3 mm, three steel beads of 1 mm diameter and glass beads of 0.75 to 1 mm diameter (Carl Roth GmbH) were added to each sample, and bead milling was performed for 3 × 1 min in a homogenizer (FastPrep24, MP Biomedicals). The combined extracts were centrifuged and stored on ice until measurement on the same day.

IAA was separated on a Nucleoshell RP Biphenyl column (100 mm × 2 mm, 2.1 μm, Macherey und Nagel, Düren, Germany) with the following gradient: 0–2 min: 95% A and 5% B, 13 min: 5% A and 95% B, 13–15 min: 5% A and 95% B, and 15–18 min: 95% A and 5% B. The column temperature was 40°C, solvent A was 0.3 mM ammonium formate, acidified with formic acid to pH 3.0, and solvent B was acetonitrile. The autosampler temperature was maintained at 4°C. Per sample 600 μl of plant extract was injected into a divinylbenzene stationary-phase micro-SPE cartridge (SparkHolland B.V., Emmen, The Netherlands) at a rate of 200 μl min^{-1} where IAA is trapped by simultaneous addition of excess water (3,800 μl min^{-1}). Transfer from the SPE cartridge to the UPLC column was accomplished by 120 μl 20% acetonitrile under continuous dilution with water, which gave a final share of 2.5% acetonitrile on-column. The entire procedure was conducted on a prototype device consisting of a CTC Combi-PAL autosampler equipped with a 1 ml injection loop, an ACE 96-well plate SPE unit, a high-pressure dispenser, a SPH1299 UPLC gradient pump, an EPH30 UPLC dilution pump, and a Mistral column oven (all Axel-Semrau GmbH, Sprockhövel, Germany).

Mass spectrometric detection of IAA on a QTrap 6500 (Sciex) was accomplished by electrospray ionization in positive mode and multiple reaction monitoring (MRM). IAA quantification was made based on transition 176/130 and was confirmed by transition 176/103 using these parameters: declustering potential: 81 V, collision energy: 27 and 46 V, and cell exit potential: 8 and 11 V, respectively. For this, the ion source was heated to 450°C. Curtain gas 35 psi, ion source GS1 was set to 60 psi, GS2: 70 psi, and the electrospray ion spray voltage was 5,500 V. IAA quantification was performed based on authentic IAA (Olchemin, Olomouc, Czech Republic).

Confocal microscopy of PIN-GFP reporters

Plants were stratified at 4°C for 2 days, grown on standard ½ MS media containing 1% sucrose in a 16/8 h light/dark cycle at 20 or 28°C for 5 days. For short-term 20–28°C shift experiments, plants grown at 20°C were incubated for an additional 4 h at 28°C. All plants were fixed and cleared using a previously established Clearsee-based protocol (Kurihara *et al*, 2015) modified to include Calcofluor White for staining of cell walls (Ursache *et al*, 2018). Briefly, plants were fixed in 4% paraformaldehyde in 1× PBS for 1 h followed by three brief washes in 1× PBS and incubated overnight in Clearsee solution containing Calcofluor White. Images were acquired on a Zeiss LSM-980 confocal system equipped with an Airyscan 2 detector using either a 40× (1.0 NA) water immersion objective or a 63× (1.4 NA) oil immersion for Airyscan images. Calcofluor White signal was detected using a 405 nm laser for excitation and an emission window from 420 to 430 nm. For GFP, excitation was achieved using a 488 nm laser and the emission window was 500–525 nm. For RFP, excitation was achieved at 561 nm, and emission was collected at 580–620 nm. All images were acquired as non-saturated 16-bit sequential scans for further quantification.

For the measurement of basal-to-lateral PIN2-GFP signal, seedlings have been grown for 5 days on ATS media in a 16/8 h light/dark cycle at 20 or 28°C. The basal-to-lateral GFP signal of single cells in the cortical cell file was determined where indicated. GFP signal intensity was measured by using ImageJ software, and at least 10 meristem cortical cells per seedling were taken into the measurement.

RT–qPCR expression analyses

Col-0 seedlings were cultivated at either 20 or 28°C for 5 days in long-day photoperiods (16/8 h) in 90 μmol m^{-2} s^{-1} PAR from white fluorescent lamps (T5 4000K). Seedlings were harvested at Zeitgeber time (ZT) 1 and dissected by cutting off whole roots or root tips to perform expression analysis. Total RNA was extracted from three biological replicates using the NucleoSpin RNA Plant Kit (Macherey-Nagel). First-strand cDNA was synthesized using the

© 2023 The Authors

PrimeScript RT Reagent Kit (Perfect Real Time) from Takara Bio. qPCR analyses were performed on an AriaMx Real-Time PCR System (Agilent) using Absolute Blue Low Rox Mix (Thermo Fisher Scientific). *AT1G13320* was used as a reference gene (Czechowski *et al*, 2005) to calculate relative expression values ($2^{\Delta Ct}$ values). Oligonucleotide primers used in the analysis are as follows: PIN1-RT-F GGAGACTTAAGTAGGAGCTCAGCA, PIN1-RT-R CCAAAAGAGG AAACACGAATG; PIN2-RT-F GGTTGAAGCTTGAAGGTAGTCGC, PI N2-RT-R TGAAATGTTTCTTTCTCCACGCA; PIN3-RT-F CGGCTCCG AATCCAGAGTT, PIN3-RT-R ATGGCTGTTTGACTTGCCGC; and PIN4-RT-F CAACCCAAAATCATTGCTTGTG, PIN4-RT-R CGGACC GGTTATAAATCTGACC, PP2A-RT-F ACACAAGGTTCACAATCC GTG, and PP2A-RT-R CATTCAGGACCAAACTCTTCAGC.

Data availability

This study includes no data deposited in external repositories.

Expanded View for this article is available online.

Acknowledgements

The authors are thankful for the support of this work by the Deutsche Forschungsgemeinschaft (Qu 141/3-2 and Qu 141/12-1 to MQ) as well as stipends from the China Scholarship Council to HA and the Rosa-Luxemburg-Stiftung to JB. TGA thanks the Sofia Kovalevskaia program of the Alexander von Humboldt Foundation and the Max Planck Society for funding. GUB, AT, and BH received core funding from the Leibniz Institute of Plant Biochemistry. We would furthermore like to thank Elisabeth Beate Truernit for supporting KSB, Elena Feraru, and Jürgen Kleine-Vehn for discussing unpublished data, and Jiri Friml and Dorata Jaworska for generously and repeatedly sharing seed stocks. Likewise, we would like to thank Helene Robert Boisivon, Christian Luschnig, Shui Wang, Lieven de Veylder, Zoltan Magyar, and Carolin Alfs for providing mutant seeds. Open Access funding enabled and organized by Projekt DEAL.

Author contributions

Marcel Quint: Conceptualization; supervision; funding acquisition; writing – original draft; project administration; writing – review and editing. **Haiyue Ai:** Conceptualization; data curation; formal analysis; investigation; visualization; writing – original draft; writing – review and editing. **Julia Bellstaedt:** Conceptualization; investigation. **Kai Steffen Bartusch:** Investigation. **Lennart Eschen-Lippold:** Supervision; writing – review and editing. **Steve Babben:** Investigation; writing – review and editing. **Gerd Ulrich Balcke:** Investigation; writing – review and editing. **Alain Tissier:** Resources; writing – review and editing. **Bettina Hause:** Resources; writing – review and editing. **Tonni Grube Andersen:** Investigation; writing – review and editing. **Carolin Delker:** Conceptualization; supervision; visualization; project administration; writing – review and editing.

Disclosure and competing interests statement

The authors declare that they have no conflict of interest.

References

Alonso JM, Stepanova AN, Leisse TJ, Kim CJ, Chen H, Shinn P, Stevenson DK, Zimmerman J, Barajas P, Cheuk R *et al* (2003) Genome-wide insertional mutagenesis of *Arabidopsis thaliana*. *Science* 301: 653 – 657

Belén Borniego M, Costigliolo-Rojas C, Casal JJ (2022) Shoot thermosensors do not fulfil the same function in the root. *New Phytol* 236: 9 – 14

Bellstaedt J, Trenner J, Lippmann R, Poeschl Y, Zhang X, Friml J, Quint M, Delker C (2019) A Mobile auxin signal connects temperature sensing in cotyledons with growth responses in hypocotyls. *Plant Physiol* 180: 757 – 766

Benková E, Michniewicz M, Sauer M, Teichmann T, Seifertová D, Jürgens G, Friml J (2003) Local, efflux-dependent auxin gradients as a common module for plant organ formation. *Cell* 115: 591 – 602

Berckmans B, Vassileva V, Schmid SP, Maes S, Parizot B, Naramoto S, Magyar Z, Alvim Kamei CL, Koncz C, Bögre L *et al* (2011) Auxin-dependent cell cycle reactivation through transcriptional regulation of Arabidopsis E2Fa by lateral organ boundary proteins. *Plant Cell* 23: 3671 – 3683

Blilou I, Xu J, Wildwater M, Willemsen V, Paponov I, Friml J, Heidstra R, Aida M, Palme K, Scheres B (2005) The PIN auxin efflux facilitator network controls growth and patterning in *Arabidopsis* roots. *Nature* 433: 39 – 44

Burstrom H (1953) Studies on growth and metabolism of roots IX. Cell elongation and water absorption. *Physiol Plant* 6: 262 – 276

Carmona M, Cuadrado A (1986) Analysis of growth components in allium roots. *Planta* 91: 87 – 95

Casal JJ, Balasubramanian S (2019) Thermomorphogenesis. *Annu Rev Plant Biol* 70: 321 – 346

Castroverde C, Dina D (2021) Temperature regulation of plant hormone signaling during stress and development. *J Exp Bot* 72: 7436 – 7458

Chen Q, Dai X, De-Paoli H, Cheng Y, Takebayashi Y, Kasahara H, Kamiya Y, Zhao Y (2014) Auxin overproduction in shoots cannot rescue auxin deficiencies in Arabidopsis roots. *Plant Cell Physiol* 55: 1072 – 1079

Chen X, Yao Q, Gao X, Jiang C, Harberd NP, Fu X (2016) Shoot-to-root mobile transcription factor HY5 coordinates plant carbon and nitrogen acquisition. *Curr Biol* 26: 640 – 646

Chung BYW, Balcerowicz M, Di Antonio M, Jaeger KE, Geng F, Franaszek K, Marriott P, Brierley I, Firth AE, Wigge PA (2020) An RNA thermoswitch regulates daytime growth in Arabidopsis. *Nat Plants* 6: 522 – 532

Crawford AJ, McLachlan DH, Hetherington AM, Franklin KA (2012) High temperature exposure increases plant cooling capacity. *Curr Biol* 22: R396 – R397

Czechowski T, Stitt M, Altmann T, Udvardi MK, Scheible W-R (2005) Genome-wide identification and testing of superior reference genes for transcript normalization in *Arabidopsis*. *Plant Physiol* 139: 5 – 17

de Lima CFF, Kleine-Vehn J, De Smet I, Feraru E (2021) Getting to the root of belowground high temperature responses in plants. *J Exp Bot* 72: 7404 – 7413

Del Pozo JC, Manzano C (2014) Auxin and the ubiquitin pathway. Two players-one target: the cell cycle in action. *J Exp Bot* 65: 2617 – 2632

Delker C, Sonntag L, James GV, Janitza P, Ibañez C, Ziermann H, Peterson T, Denk K, Mull S, Ziegler J *et al* (2014) The DET1-COP1-HY5 pathway constitutes a multipurpose signaling module regulating plant photomorphogenesis and thermomorphogenesis. *Cell Rep* 9: 1983 – 1989

Delker C, van Zanten M, Quint M (2017) Thermosensing enlightened. *Trends Plant Sci* 22: 185 – 187

Delker C, Quint M, Wigge PA (2022) Recent advances in understanding thermomorphogenesis signaling. *Curr Opin Plant Biol* 68: 102231

Erickson RO (1959) Integration of plant growth processes. *Am Nat* 93: 225 – 235

Feraru E, Feraru MI, Barbez E, Waidmann S, Sun L, Gaidora A, Kleine-Vehn J (2019) PILS6 is a temperature-sensitive regulator of nuclear auxin input and organ growth in *Arabidopsis thaliana*. *Proc Natl Acad Sci USA* 116: 3893 – 3898

Friml J, Benková E, Blilou I, Wisniewska J, Hamann T, Ljung K, Woody S, Sandberg G, Scheres B, Jürgens G *et al* (2002) AtPIN4 mediates sink-driven auxin gradients and root patterning in *Arabidopsis*. *Cell* 108: 661 – 673

© 2023 The Authors

Friml J, Vieten A, Sauer M, Weijers D, Schwarz H, Hamann T, Offringa R, Jürgens G (2003) Efflux-dependent auxin gradients establish the apical-basal axis of Arabidopsis. *Nature* 426: 147 – 153

Gaillochet C, Burko Y, Platre MP, Zhang L, Simura J, Willige BC, Kumar SV, Ljung K, Chory J, Busch W (2020) HY5 and phytochrome activity modulate shoot-to-root coordination during thermomorphogenesis in Arabidopsis. *Development* 147: dev192625

González-García MP, Conesa CM, Lozano-Enguita A, Baca-González V, Simancas B, Navarro-Neila S, Sánchez-Bermúdez M, Salas-González I, Caro E, Castrillo G *et al* (2022) Temperature changes in the root ecosystem affect plant functionality. *Plant Commun* https://doi.org/10.1016/j.xplc.2022.100514

Grieneisen VA, Xu J, Marée AF, Hogeweg P, Scheres B (2007) Auxin transport is sufficient to generate a maximum and gradient guiding root growth. *Nature* 449: 1008 – 1013

Grif VG, Valovich EM (1973) Influence of low positive temperatures on cell growth and division in germinating seed development. *Tsitologiia* 15: 1362 – 1369

Grif VG, Ivanov VB, Machs EM (2002) Cell cycle and its parameters in flowering plants. *Tsitologiia* 44: 936 – 980

Hanzawa T, Shibasaki K, Numata T, Kawamura Y, Gaude T, Rahman A (2013) Cellular auxin homeostasis under high temperature is regulated through a sorting NEXIN1-dependent endosomal trafficking pathway. *Plant Cell* 25: 3424 – 3433

Hayashi K, Neve J, Hirose M, Kuboki A, Shimada Y, Kepinski S, Nozaki H (2012) Rational design of an auxin antagonist of the SCFTIR1 auxin receptor complex. *ACS Chem Biol* 7: 590 – 598

He W, Brumos J, Li H, Ji Y, Ke M, Gong X, Zeng Q, Li W, Zhang X, An F *et al* (2011) A small-molecule screen identifies L-kynurenine as a competitive inhibitor of TAA1/TAR activity in ethylene-directed auxin biosynthesis and root growth in Arabidopsis. *Plant Cell* 23: 3944 – 3960

Hicks KA, Millar AJ, Carré IA, Somers DE, Straume M, Meeks-Wagner DR, Kay SA (1996) Conditional circadian dysfunction of the *Arabidopsis* early-flowering 3 mutant. *Science* 274: 790 – 792

Hu W, Franklin KA, Sharrock RA, Jones MA, Harmer SL, Lagarias JC (2013) Unanticipated regulatory roles for Arabidopsis phytochromes revealed by null mutant analysis. *Proc Natl Acad Sci USA* 110: 1542 – 1547

Ibañez C, Delker C, Martinez C, Bürstenbinder K, Janitza P, Lippmann R, Ludwig W, Sun H, James GV, Klecker M *et al* (2018) Brassinosteroids dominate hormonal regulation of plant thermomorphogenesis via BZR1. *Curr Biol* 28: 303 – 310

Ibañez C, Poeschl Y, Peterson T, Bellstädt J, Denk K, Gogol-Döring A, Quint M, Delker C (2017) Ambient temperature and genotype differentially affect developmental and phenotypic plasticity in *Arabidopsis thaliana*. *BMC Plant Biol* 17: 114

Jung JH, Domijan M, Klose C, Biswas S, Ezer D, Gao M, Khattak AK, Box MS, Charoensawan V, Cortijo S *et al* (2016) Phytochromes function as thermosensors in Arabidopsis. *Science* 354: 886 – 889

Jung JH, Barbosa AD, Hutin S, Kumita JR, Gao M, Derwort D, Silva CS, Lai X, Pierre E, Geng F *et al* (2020) A prion-like domain in ELF3 functions as a thermosensor in Arabidopsis. *Nature* 585: 256 – 260

Koevoets IT, Venema JH, Elzenga JT, Testerink C (2016) Roots withstanding their environment: exploiting root system architecture responses to abiotic stress to improve crop tolerance. *Front Plant Sci* 7: 1335

Koini MA, Alvey L, Allen T, Tilley CA, Harberd NP, Whitelam GC, Franklin KA (2009) High temperature-mediated adaptations in plant architecture require the bHLH transcription factor PIF4. *Curr Biol* 19: 408 – 413

Kurihara D, Mizuta Y, Sato Y, Higashiyama T (2015) ClearSee: a rapid optical clearing reagent for whole-plant fluorescence imaging. *Development* 142: 4168 – 4179

Lee S, Wang W, Huq E (2021) Spatial regulation of thermomorphogenesis by HY5 and PIF4 in Arabidopsis. *Nat Commun* 12: 3656

Legris M, Klose C, Burgie ES, Rojas CC, Neme M, Hiltbrunner A, Wigge PA, Schäfer E, Vierstra RD, Casal JJ (2016) Phytochrome B integrates light and temperature signals in Arabidopsis. *Science* 354: 897 – 900

Leivar P, Monte E, Oka Y, Liu T, Carle C, Castillon A, Huq E, Quail PH (2008) Multiple phytochrome-interacting bHLH transcription factors repress premature seedling photomorphogenesis in darkness. *Curr Biol* 18: 1815 – 1823

Lincoln C, Britton JH, Estelle M (1990) Growth and development of the axr1 mutants of *Arabidopsis*. *Plant Cell* 2: 1071 – 1080

Lippmann R, Babben S, Menger A, Delker C, Quint M (2019) Development of wild and cultivated plants under global warming conditions. *Curr Biol* 29: R1326 – R1338

Ludwig W, Hayes S, Trenner J, Delker C, Quint M (2021) On the evolution of plant thermomorphogenesis. *J Exp Bot* 72: 7345 – 7358

Luschnig C, Gaxiola RA, Grisafi P, Fink GR (1998) EIR1, a root-specific protein involved in auxin transport, is required for gravitropism in *Arabidopsis thaliana*. *Genes Dev* 12: 2175 – 2187

Martins S, Montiel-Jorda A, Cayrel A, Huguet S, Roux CP, Ljung K, Vert G (2017) Brassinosteroid signaling-dependent root responses to prolonged elevated ambient temperature. *Nat Commun* 8: 309

McNellis TW, von Arnim AG, Araki T, Komeda Y, Miséra S, Deng XW (1994) Genetic and molecular analysis of an allelic series of cop1 mutants suggests functional roles for the multiple protein domains. *Plant Cell* 6: 487 – 500

Melnyk CW (2017) Grafting with *Arabidopsis thaliana*. *Methods Mol Biol* 1497: 9 – 18

Müller A, Guan C, Gälweiler L, Tänzler P, Huijser P, Marchant A, Parry G, Bennett M, Wisman E, Palme K (1998) AtPIN2 defines a locus of *Arabidopsis* for root gravitropism control. *EMBO J* 17: 6903 – 6911

Murin A (1966) The effect of temperature on the mitotic cycle and its time parameters in root tips of *Vicia faba*. *Naturwissenschaften* 53: 312 – 313

Murin A (1981) Regulation of the mitotic cycle in seedlings of *Vicia faba* by environmental factors. In *Structure and Function of Plant Roots. Developments in Plant and Soil Sciences*, Brouwer R, Gašparíková O, Kolek J, Loughman BC (eds), Vol. 4. Dordrecht: Springer

Nishimura K, Fukagawa T, Takisawa H, Kakimoto T, Kanemaki M (2009) An auxin-based degron system for the rapid depletion of proteins in nonplant cells. *Nat Methods* 6: 917 – 922

Nishimura T, Hayashi K, Suzuki H, Gyohda A, Takaoka C, Sakaguchi Y, Matsumoto S, Kasahara H, Sakai T, Kato J *et al* (2014) Yucasin is a potent inhibitor of YUCCA, a key enzyme in auxin biosynthesis. *Plant J* 77: 352 – 366

Nozue K, Covington MF, Duek PD, Lorrain S, Fankhauser C, Harmer SL, Maloof JN (2007) Rhythmic growth explained by coincidence between internal and external cues. *Nature* 448: 358 – 361

Okada K, Ueda J, Komaki MK, Bell CJ, Shimura Y (1991) Requirement of the auxin polar transport system in early stages of *Arabidopsis* floral bud formation. *Plant Cell* 3: 677 – 684

Park YJ, Lee HJ, Gil KE, Kim JY, Lee JH, Lee H, Cho HT, Vu LD, De Smet I, Park CM (2019) Developmental programming of thermonastic leaf movement. *Plant Physiol* 180: 1185 – 1197

Parry G, Calderon-Villalobos LI, Prigge M, Peret B, Dharmasiri S, Itoh H, Lechner E, Gray WM, Bennett M, Estelle M (2009) Complex regulation of

 © 2023 The Authors

the TIR1/AFB family of auxin receptors. *Proc Natl Acad Sci USA* 106: 22540 – 22545

Perrot-Rechenmann C (2010) Cellular responses to auxin: division versus expansion. *Cold Spring Harb Perspect Biol* 2: a001446

Quint M, Ito H, Zhang W, Gray WM (2005) Characterization of a novel temperature-sensitive allele of the CUL1/AXR6 subunit of SCF ubiquitin-ligases. *Plant J* 43: 371 – 383

Quint M, Barkawi LS, Fan KT, Cohen JD, Gray WM (2009) Arabidopsis IAR4 modulates auxin response by regulating auxin homeostasis. *Plant Physiol* 150: 748 – 758

Quint M, Delker C, Franklin KA, Wigge PA, Halliday KJ, Zanten MV (2016) Molecular and genetic control of plant thermomorphogenesis. *Nat Plants* 2: 15190

Reed JW, Nagatani A, Elich TD, Fagan M, Chory J (1994) Phytochrome A and phytochrome B have overlapping but distinct functions in *Arabidopsis* development. *Plant Physiol* 104: 1139 – 1149

Saini K, Dwivedi A, Ranjan A (2022) High temperature restricts cell division and leaf size by coordination of PIF4 and TCP4 transcription factors. *Plant Physiol* 190: 2380 – 2397

Sauer M, Balla J, Luschnig C, Wiśniewska J, Reinöhl V, Friml J, Benková E (2006) Canalization of auxin flow by Aux/IAA-ARF-dependent feedback regulation of PIN polarity. *Genes Dev* 20: 2902 – 2911

Scanlon MJ (2003) The polar auxin transport inhibitor N-1-naphthylphthalamic acid disrupts leaf initiation, KNOX protein regulation, and formation of leaf margins in maize. *Plant Physiol* 133: 597 – 605

Serivichyaswat PT, Bartusch K, Leso M, Musseau C, Iwase A, Chen Y, Sugimoto K, Quint M, Melnyk CW (2022) High temperature perception in leaves promotes vascular regeneration and graft formation in distant tissues. *Development* 149: dev200079

Silk WK (1992) Steady form from changing cells. *Int J Plant Sci* 153: S49 – S58

Silk WK, Lord EM, Eckard KJ (1989) Growth patterns inferred from anatomical records: empirical tests using longisections of roots of *Zea mays* L. *Plant Physiol* 90: 708 – 713

Stepanova AN, Robertson-Hoyt J, Yun J, Benavente LM, Xie DY, Dolezal K, Schlereth A, Jürgens G, Alonso JM (2008) TAA1-mediated auxin biosynthesis is essential for hormone crosstalk and plant development. *Cell* 133: 177 – 191

Su YS, Lagarias JC (2007) Light independent phytochrome sig-naling mediated by dominant GAF-domain tyrosine mutants of Arabidopsis phytochromes in transgenic plants. *Plant Cell* 19: 2124 – 2139

Ursache R, Andersen TG, Marhavý P, Geldner N (2018) A protocol for combining fluorescent proteins with histological stains for diverse cell wall components. *Plant J* 93: 399 – 412

van Gelderen K, Kang C, Paalman R, Keuskamp D, Hayes S, Pierik R (2018) Far-red light detection in the shoot regulates lateral root development through the HY5 transcription factor. *Plant Cell* 30: 101 – 116

Wang R, Zhang Y, Kieffer M, Yu H, Kepinski S, Estelle M (2016) HSP90 regulates temperature-dependent seedling growth in Arabidopsis by stabilizing the auxin co-receptor F-box protein TIR1. *Nat Commun* 7: 10269

Weijers D, Schlereth A, Ehrismann JS, Schwank G, Kientz M, Jürgens G (2006) Auxin triggers transient local signaling for cell specification in Arabidopsis embryogenesis. *Dev Cell* 10: 265 – 270

Yang X, Lee S, So JH, Dharmasiri S, Dharmasiri N, Ge L, Jensen C, Hangarter R, Hobbie L, Estelle M (2004) The IAA1 protein is encoded by AXR5 and is a substrate of SCF(TIR1). *Plant J* 40: 772 – 782

Yang X, Dong G, Palaniappan K, Mi G, Baskin TI (2017) Temperature-compensated cell production rate and elongation zone length in the root of *Arabidopsis thaliana*. *Plant Cell Environ* 40: 264 – 276

Yao X, Yang H, Zhu Y, Xue J, Wang T, Song T, Yang Z, Wang S (2018) The canonical E2Fs are required for germline development in Arabidopsis. *Front Plant Sci* 9: 638

Žádníková P, Petrášek J, Marhavý P, Raz V, Vandenbussche F, Ding Z, Schwarzerová K, Morita MT, Tasaka M, Hejátko J *et al* (2010) Role of PIN-mediated auxin efflux in apical hook development of *Arabidopsis thaliana*. *Development* 137: 607 – 617

Zanten MV, Ai H, Quint M (2021) Plant thermotropism: an underexplored thermal engagement and avoidance strategy. *J Exp Bot* 72: 7414 – 7420

Zhu J, Zhang KX, Wang WS, Gong W, Liu WC, Chen HG, Xu HH, Lu YT (2015) Low temperature inhibits root growth by reducing auxin accumulation via ARR1/12. *Plant Cell Physiol* 56: 727 – 736

License: This is an open access article under the terms of the Creative Commons Attribution License, which permits use, distribution and reproduction in any medium, provided the original work is properly cited.

Expanded View Figures

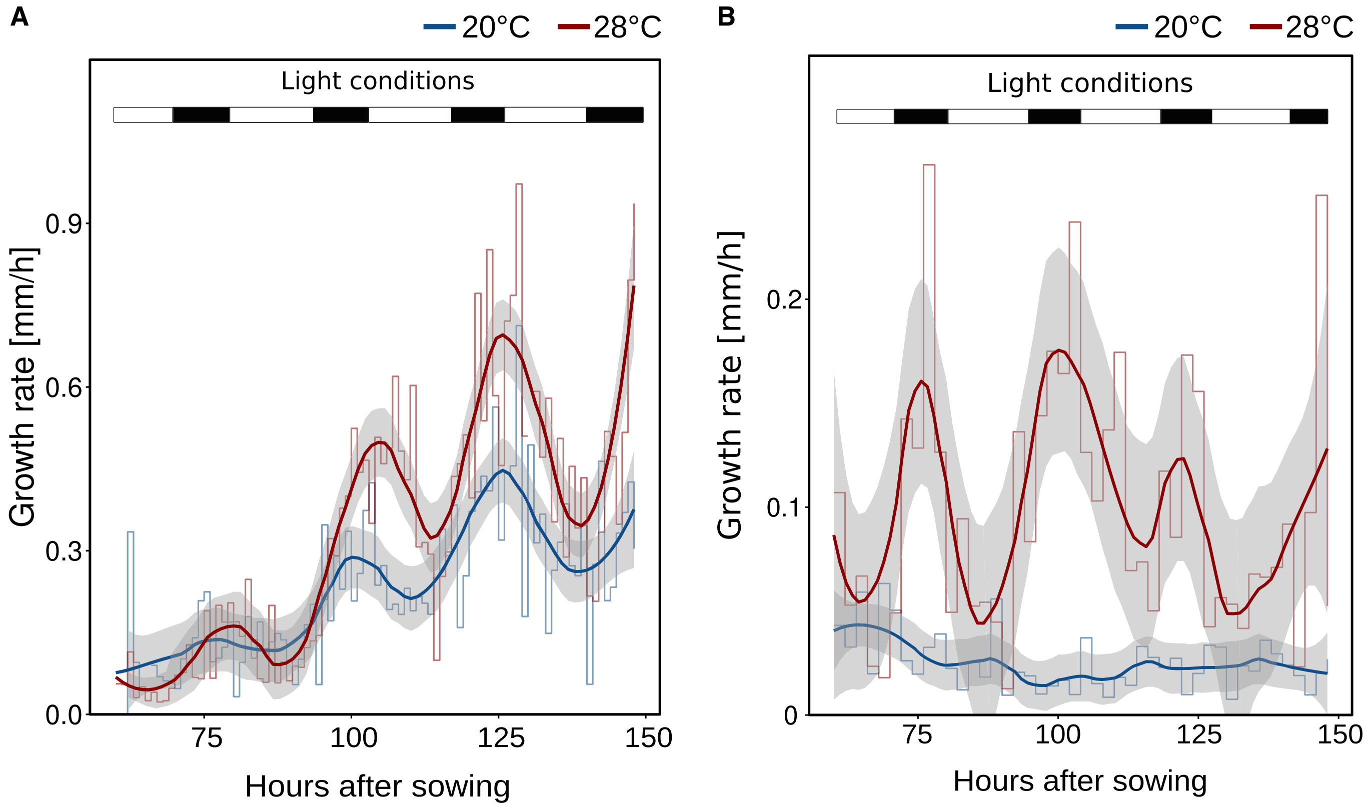

Figure EV1. Growth dynamics in response to different temperatures.

A, B Growth rates of seedling (A) roots and (B) hypocotyls between days 2 and 7 were assessed by hourly and 2-hourly infra-red real-time imaging, respectively. Mean growth rates are shown as step-wise lines (n = 7–8) that were fitted with a "loess" smoothing function shown as solid lines and the respective 95% confidence intervals are shown as gray ribbons. Long-day lighting is schematically depicted by white (light) and black (dark) bars on the top.

Source data are available online for this figure.

© 2023 The Authors

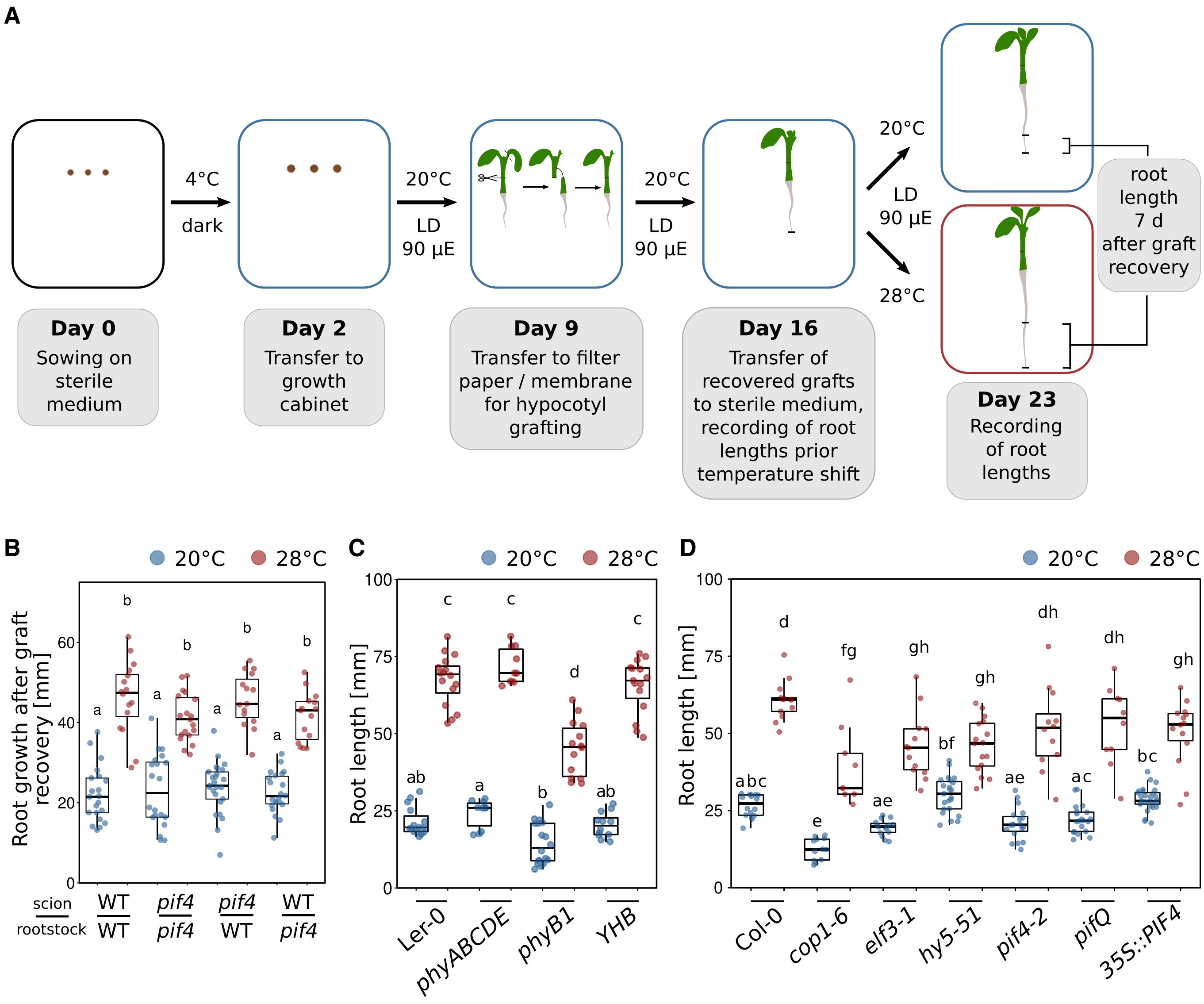

Figure EV2. Micrografting assays and root temperature responses in selected mutants.

A, B (A) Schematic representation of the grafting experiments shown in (B) and Fig 2B–D. Temperature-induced root elongation of (B) *pif4-2* mutants grafted with their corresponding wild-type Col-0 (n = 15–25).

C, D Root growth assay of seedlings grown for 7 days (C: n = 10–18, D: n = 9–29) at the indicated temperatures. The *phyB* Y276H mutant (YHB; Su & Lagarias, 2007) in (C) adopts a light-insensitive, physiologically active conformation and can be described as constitutively active. (B–D) Boxplots show medians, interquartile ranges, and min–max values with individual data points superimposed as colored dots. Different letters denote statistical differences at $P < 0.05$ as assessed by two-way ANOVA and Tukey's HSD *post hoc* test.

Source data are available online for this figure.

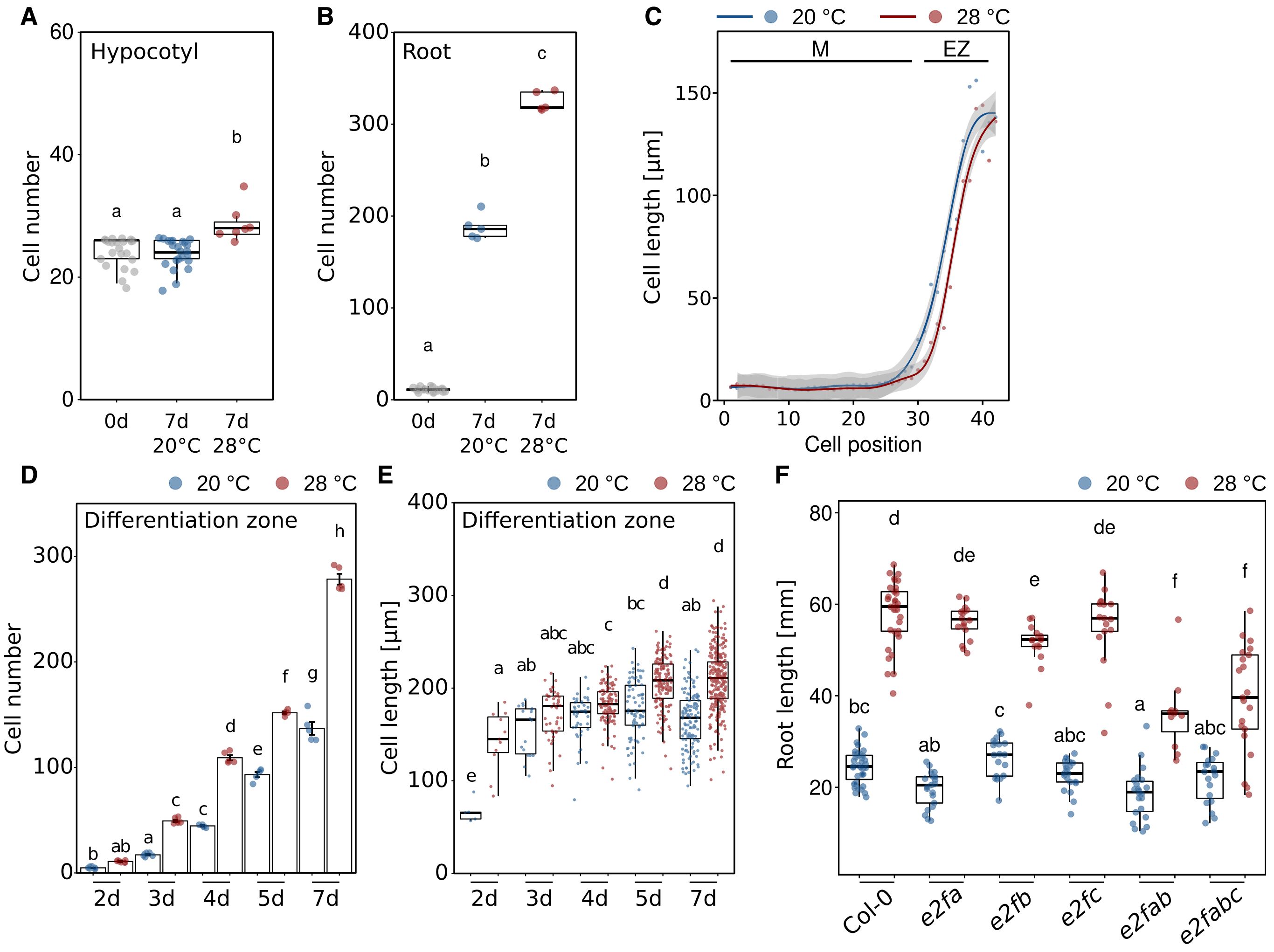

Figure EV3. Changes in root and shoot cell numbers from mature embryos to 7-day-old seedlings.

A, B Number of cells in a consecutive cell file of (A) hypocotyls and (B) roots of mature embryos prior to germination (0 days) and in 7-day-old seedlings grown at 20 or 28°C.

C Close-up view of the first 43 cells comprising meristem (M) and elongation zone (EZ) of data shown in Fig 3D.

D Total number of cells in a longitudinal cortex cell file of the differentiation zone between days 2 and 7 after sowing. Barplots show mean values, error bars indicate SEM. Individual data points are plotted as colored dots (n = 5–7).

E Mean lengths of all cells in a consecutive cell file of the differentiation zone measured in 5–7 individual roots. Number of cells ranges from n = 5 (2 days, 20°C) to n = 272 (7 days, 28°C).

F Temperature-induced root elongation in 7-day-old wild-type, single-, and higher-order *e2f* mutants (n = 11–39). Boxplots show medians, interquartile ranges, and min–max values with individual data points superimposed as colored dots. Different letters denote statistical differences at $P < 0.05$ as assessed by one-way ANOVA (A, B) or two-way ANOVA (D–F) and Tukey's HSD *post hoc* test.

Source data are available online for this figure.

 © 2023 The Authors

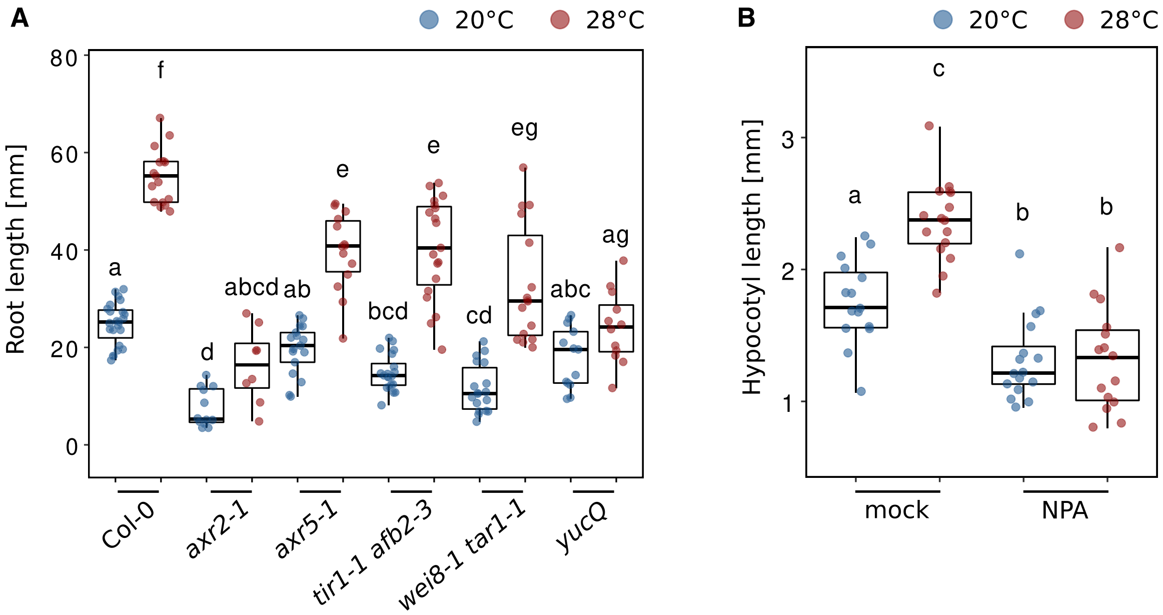

Figure EV4. Temperature-induced root elongation in auxin biosynthesis and signaling mutants.

A Root length of 7-day-old Arabidopsis seedlings grown in LD at 20 or 28°C (n = 8–23).

B Temperature-induced hypocotyl elongation in 8-day-old seedlings with mock or 0.5 mM NPA applied to the cotyledons (n = 15–16). (A, B) Boxplot shows medians, interquartile ranges, and min–max values with individual data points superimposed as colored dots. Different letters denote statistical differences at $P < 0.05$ as assessed by two-way ANOVA and Tukey's HSD *post hoc* test.

Source data are available online for this figure.

© 2023 The Authors

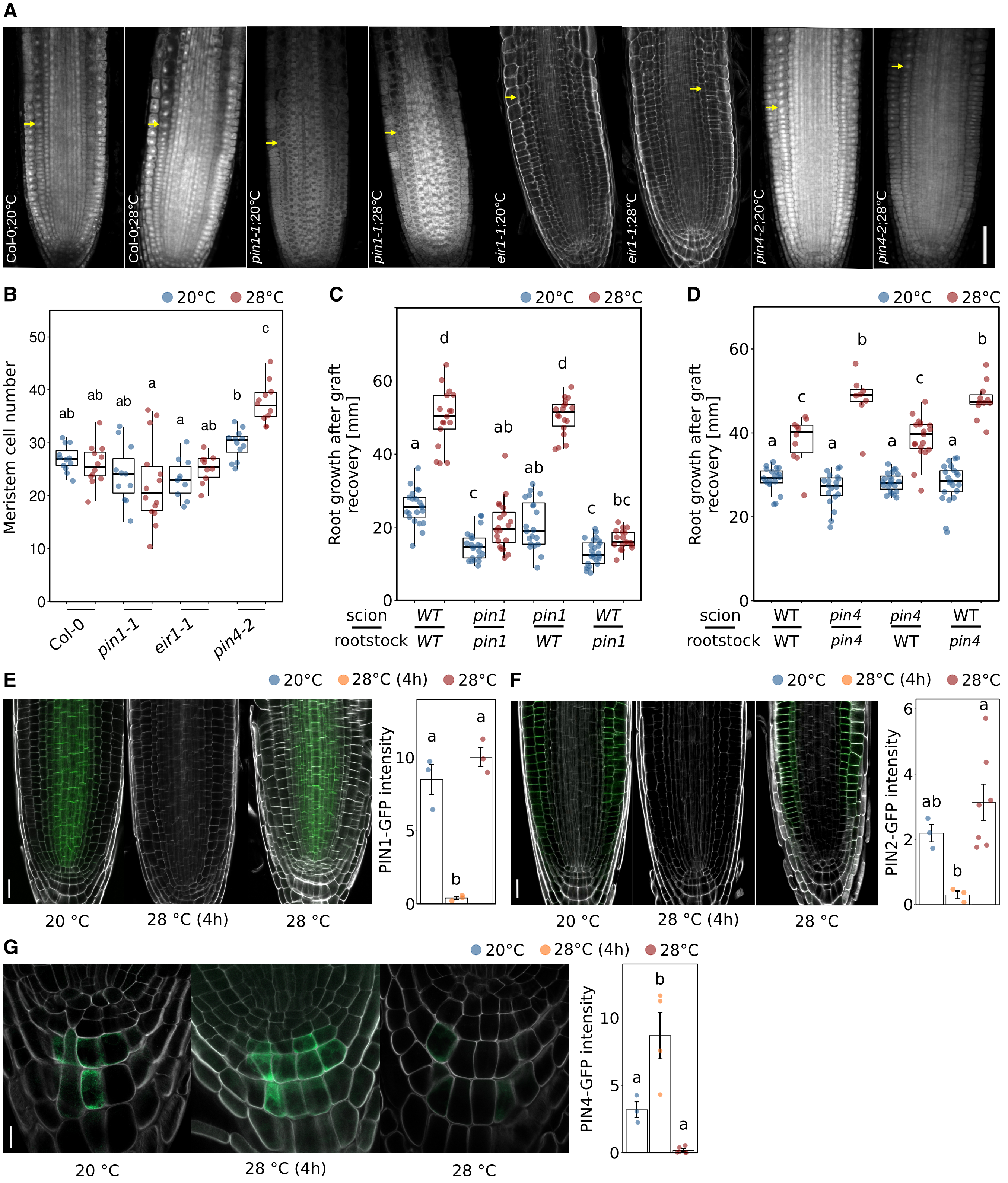

Figure EV5.

© 2023 The Authors

Figure EV5. PIN1 and PIN2 positively regulate root thermomorphogenesis.

A Microscopic photographs of root tips of 5-day-old seedlings grown in LD at 20 or 28°C. Yellow arrows mark the end of the meristem. Scale bar = 50 μm.

B Quantification of meristem cell numbers in consecutive cortex cell files. Root growth assay of 7-day-old seedlings (n = 13–30) grown at the indicated temperatures.

C, D Nine-day-old seedlings grown at 20°C were hypocotyl grafted, recovered for 7 days, and then cultivated at 20 or 28°C for another 7 days (C: n = 17–25, D: n = 8–23).

E–G Temperature effects on PIN1-GFP (E, scale bar = 20 μm), PIN2-GFP (F, scale bar = 20 μm), and PIN4-GFP (G, scale bar = 10 μm) levels. Seedlings were grown for 5 days at constant temperatures (20 or 28°C) or grown at 20°C for 5 days and shifted to 28°C for 4 h (n = 3–7). (B–G) Boxplots show medians, interquartile ranges, and min–max values (B–D). Barplots show mean values and error bars show SEM (E–G). Individual data points superimposed as colored dots. Different letters denote statistical differences at $P < 0.05$ as assessed by one-way (E–G) or two-way (B–D) ANOVA and Tukey's HSD *post hoc* test.

Source data are available online for this figure.

© 2023 The Authors
