# Supplementary material for: Auxin‐dependent regulation of cell division rates governs root thermomorphogenesis
Source: EMBO J. 2023 Apr 18;42(11):e111926. doi: 10.15252/embj.2022111926 (PMC10233379; doi:10.15252/embj.2022111926)
Supplement: Supplementary file 4 — Source Data for Figure 1 [file EMBJ-42-e111926-s002.zip › Figure1/Figure1_README.rtf]

Seeds were surface sterilized, rinsed with sterile water, and then imbibed and stratified for 3 days at 4°C in deionized water before sowing. Seeds were placed on ATS medium and grown at 20°C for 4 days (A. thaliana and B. oleracea) or 5 days (S. lycopersicum) prior to shoot removal. Detached roots were incubated for additional 4 days at either 20°C or 28°C after marking the root length at time of shoot removal on the plate. All measurements were based on digital photographs of plates using RootDetection (www.labutils.de) and depict the length of the primary roots grown in the 4 days after shoot removal in mm. 
