# Supplementary material for: Auxin‐dependent regulation of cell division rates governs root thermomorphogenesis
Source: EMBO J. 2023 Apr 18;42(11):e111926. doi: 10.15252/embj.2022111926 (PMC10233379; doi:10.15252/embj.2022111926)
Supplement: Supplementary file 5 — Source Data for Figure 2 [file EMBJ-42-e111926-s007.zip › Figure2/Figure2_README.rtf]

Figure 2A: Temperature response in detached rootsSeeds were surface sterilized, rinsed with sterile water, and then imbibed and stratified for 3 days at 4°C in deionized water before sowing. Seeds were placed on ATS medium and grown at 20°C for 4 days (A. thaliana and B. oleracea) or 5 days (S. lycopersicum) prior to shoot removal. Detached roots were incubated for additional 4 days at either 20°C or 28°C after marking the root length at time of shoot removal on the plate. All measurements were based on digital photographs of plates using RootDetection (www.labutils.de) and depict the length of the primary roots grown in the 4 days after shoot removal in mm. Figure2B-C: Root length of grafted seedlingSeeds were sown on ATS medium at 4°C darkness, stratified for 2 days at 4°C and then shifted to 20°C in a growth cabinet for another 7 days under long-day photoperiods (16 h of light/8 h of dark) with 90 µmol m− s− white light (T5 4000K). Next, seedlings were grafted and recovered for 7 days on a water mounted filter paper/membrane. Successfully recovered grafted plants were selected, transferred to new ATS medium and cultivated at 20°C or 28°C, respectively, under the same conditions described above for another 7 days. Root growth after graft recovery was then determined by measuring the root growth difference between day 16 and day 23 and given in mm. 
