# Supplementary material for: Auxin‐dependent regulation of cell division rates governs root thermomorphogenesis
Source: EMBO J. 2023 Apr 18;42(11):e111926. doi: 10.15252/embj.2022111926 (PMC10233379; doi:10.15252/embj.2022111926)
Supplement: Supplementary file 6 — Source Data for Figure 3 [file EMBJ-42-e111926-s009.zip › Figure3/Figure3_Readme.rtf]

Figure 3A-C:The meristematic zone was defined as the zone between the quiescent centre and the last cell that did not yet double its size in comparison with the previous cell. The elongation zone followed the meristematic zone and was defined as the zone from first cell with double the size of the previous cell to the last cell before root hair bulges became visible. The following differentiation and maturation zone was defined as the zone from first cell below the first trichoblast bulge to the root-shoot junction.Figure3D:Root cell measurements were conducted by staining seedlings with Calcofluor White (Merck, 18909-100ML-F). Briefly, seedlings were fixed in pure ethanol for 2 h to overnight, washed twice with 1x PBS, followed by a permeabilization step using 3 % Triton X-100 + 10 % DMSO in 1x PBS for 30 min to 1 h. Next, 0.1 % Calcofluor White in 1x PBS was freshly prepared and the seedlings were stained for 30 min. Subsequently, seedlings were washed twice in 1x PBS with gentle shaking. To image Calcofluor White, we used 405 nm excitation and detected signals at 425 - 475 nm. All measurements were performed on all individual cells of a consecutive cortex cell file starting at the quiescent centre (cell position =1) using the ZEN 3.1 software (Zeiss) for 8 - 12 independent seedlings per experiment. Cell length is given in µm.Figure3E:5-Ethynyl-2'-deoxyuridine (EdU) staining was performed with the EdU Click-488 Imaging Kit (Carl-Roth) according to the manufacturer’s instruction. Briefly, 5 days-old Col-0 seedlings (at ZT1, 1 h after lights on) were immersed for 1 h in liquid ATS medium containing 10 μ EdU, and fixed in 4 % (w/v) paraformaldehyde and 0.5 % Triton X-100 for 20 min. After washing twice with 1x PBS, samples were incubated in the reaction cocktail for 30 min in the dark. The reaction cocktail was then removed, and samples were washed with 1x PBS, followed by confocal microscopy with a Zeiss LSM 780 AxioObserver (excitation wavelength: 488 nm; emission wavelength: 491-585 nm). The region of interest (root meristem) was determined with the same fixed area in all measurements, and positively stained cells were counted in this area to calculate cells per 1000 μ2. Representative pictures for both temperatures were cropped and compiled into a single file using Gimp software to then adjust contrast and color intensity simultaneously on all pictures for publication and print.Figure3F:At ZT2 - ZT3 (2-3 hrs after lights on), 5 days-old Col-0 seedlings were fixed with pure ethanol for 2 h, rinsed twice with 1x PBS, followed by a permeabilization step using 3 % Triton X-100 + 10 % DMSO in 1x PBS for 30 min. Seedlings were subsequently washed three times with 1x PBS, and then stained with 100 μ mL− 4',6-diamidino-2-phenylindole (DAPI) in the dark for 15 min at room temperature, and processed under a Zeiss LSM 780 AxioObserver (excitation wavelength: 405 nm; emission wavelength: 425-508 nm). The region of interest (root meristem) was determined, and all stained nuclei in this area were counted by using the ImageJ software. The mitosis ratio was determined by counting cells in mitosis (condensed chromosomes visible) divided by all nuclei in this area. Data is given as % of cells undergoing mitosis (= mitosis ratio*100).  Representative pictures for both temperatures were cropped and compiled into a single file using Gimp software to then adjust contrast and color intensity simultaneously on all pictures for publication and print.
