# Supplementary material for: Auxin‐dependent regulation of cell division rates governs root thermomorphogenesis
Source: EMBO J. 2023 Apr 18;42(11):e111926. doi: 10.15252/embj.2022111926 (PMC10233379; doi:10.15252/embj.2022111926)
Supplement: Supplementary file 7 — Source Data for Figure 4 [file EMBJ-42-e111926-s006.zip › Figure4/Figure4_README.rtf]

Figure 4A: Influence of auxin inhibitors on root thermomorphogenesisSeedlings were placed on ATS medium, grown at 20°C or 28°C and root length was determined 7 days after sowing (unless stated otherwise). Plates were supplemented with equal amounts of auxin inhibitors kynurenine (He et al., 2011) and yucasin (Nishimura et al., 2014): 1, 5, 10 or 50 µM or mock treated. All measurements were based on digital photographs of plates using RootDetection (www.labutils.de) and depict the total length of the root in mm. Figure 4B: IAA quantificationSeeds of A. thaliana (Col-0) were surface sterilized, rinsed with sterile water, and then imbibed and stratified for 3 days at 4°C in deionized water before sowing on solid Arabidopsis thaliana solution (ATS, Lincoln et al., 1990) nutrient medium including 1 % (w/v) sucrose. Seedlings werde cultivated on vertically oriented plates under long-day conditions (16 h of light/8 h of dark) with 90 µmol m− s− photosynthetically active radiation (PAR) from white fluorescent lamps (T5 4000K) for 5 days at 20°C or 28°C. IAA was extracted as from root tips as stated in Materials and Methods section of the manuscript. Amounts are given as ng IAA per g fresh weight.Figure 4C: DR5 promotor activitySeeds of A. thaliana Col-0 and wei8-1 tar1-1 seedlings carrying DR5revp:SV40:3×GFP (DR5NLS::GFP) reporters were surface sterilized, rinsed with sterile water, and then imbibed and stratified for 3 days at 4°C in deionized water before sowing on solid Arabidopsis thaliana solution (ATS, Lincoln et al., 1990) nutrient medium including 1 % (w/v) sucrose. Seedlings werde cultivated on vertically oriented plates under long-day conditions (16 h of light/8 h of dark) with 90 µmol m− s− photosynthetically active radiation (PAR) from white fluorescent lamps (T5 4000K) for 5 days at 20°C or 28°C. Seedlings were fixed directly with 4 % (w/v) paraformaldehyde at room temperature, washed with 1x PBS, and kept in the dark until imaging (excitation wavelength: 561 nm; emission wavelength: 571-615 nm). Columella cells including the quiescent center were determined as the fixed area through all measurements. Mean grey values as a proxy for DR5 promotor activity were measured by using ImageJ. Representative pictures for each genotype and temperature were cropped and compiled into a single file using Gimp software to then adjust contrast and color intensity simultaneously on all pictures for publication and print.Figure 4D: Effect of localized NPA application to root-shoot-junctionSeeds of A. thaliana (Col-0) were surface sterilized, rinsed with sterile water, and then imbibed and stratified for 3 days at 4°C in deionized water before sowing on solid Arabidopsis thaliana solution (ATS, Lincoln et al., 1990) nutrient medium including 1 % (w/v) sucrose. Seedlings werde cultivated on vertically oriented plates under long-day conditions (16 h of light/8 h of dark) with 90 µmol m− s− photosynthetically active radiation (PAR) from white fluorescent lamps (T5 4000K) for 8 days at 20°C or 28°C. On day 5 thin tissue strips were soaked in lukewarm ATS medium with or without the addition of 0.5 mM NPA (Duchefa) and carefully placed across the root-shoot junction.Figure4E-F: Temperature-induced root elongation in presence of NPA and PEO-IAA Seeds of A. thaliana (Col-0) were surface sterilized, rinsed with sterile water, and then imbibed and stratified for 3 days at 4°C in deionized water. Seedlings were cultivated on ATS medium at 20°C or 28°C and root length was determined 7 days after sowing. Plates were supplemented with various concentrations of auxin inhibitors NPA (Scanlon, 2003), or PEO-IAA (Hayashi et al., 2012). All measurements were based on digital photographs of plates using RootDetection (www.labutils.de) and depict the total length of the root in mm. Figure 4G: Auxin effect on cell-cycle activity (EdU staining)Seeds of A. thaliana (Col-0) were surface sterilized, rinsed with sterile water, and then imbibed and stratified for 3 days at 4°C in deionized water before sowing on solid Arabidopsis thaliana solution (ATS, Lincoln et al., 1990) nutrient medium including 1 % (w/v) sucrose. Seedlings werde cultivated on vertically oriented plates under long-day conditions (16 h of light/8 h of dark) with 90 µmol m− s− photosynthetically active radiation (PAR) from white fluorescent lamps (T5 4000K) for 20°C or 28°C. 5 days-old seedlings (at ZT1, 1 h after lights on) were treated with either EdU (10 μ) + PEO-IAA (50 μ), EdU (10 μ) + NAA (100 nM), or 10 μ EdU + DMSO  (mock) in liquid ATS for 3h prior to fixation of samples in 4 % (w/v) paraformaldehyde and 0.5 % Triton X-100 for 20 min. After washing twice with 1x PBS, samples were incubated in the reaction cocktail for 30 min in the dark. The reaction cocktail was then removed, and samples were washed with 1x PBS, followed by confocal microscopy with a Zeiss LSM 780 AxioObserver (excitation wavelength: 488 nm; emission wavelength: 491-585 nm). The region of interest (root meristem) was determined with the same fixed area in all measurements, and positively stained cells were counted in this area to calculate cells per 1000 μ2. Representative pictures for each treatment were cropped and compiled into a single file using Gimp software to then adjust contrast and color intensity simultaneously on all pictures for publication and print.
