# Supplementary material for: Auxin‐dependent regulation of cell division rates governs root thermomorphogenesis
Source: EMBO J. 2023 Apr 18;42(11):e111926. doi: 10.15252/embj.2022111926 (PMC10233379; doi:10.15252/embj.2022111926)
Supplement: Supplementary file 8 — Source Data for Figure 5 [file EMBJ-42-e111926-s005.zip › Figure5/Figure5_README.rtf]

Figure 5A: Temperature-induced root elongation in pin mutantsSurface sterilized seeds were rinsed with sterile water and then imbibed and stratified for 3 days at 4°C in deionized water before sowing on solid Arabidopsis thaliana solution (ATS, Lincoln et al., 1990) nutrient medium including 1 % (w/v) sucrose on vertically oriented plates under long-day conditions (16 h of light/8 h of dark) with 90 µmol m− s− photosynthetically active radiation (PAR) from white fluorescent lamps (T5 4000K) at 20 or 28°C. Measurements were based on digital photographs of plates after 7 days of cultivation using RootDetection (www.labutils.de) and depict the total length of the root in mm. Figure 5B: DR5 promotor activity in pin mutant backgroundsSurface sterilized seeds were rinsed with sterile water and then imbibed and stratified for 3 days at 4°C in deionized water before sowing on solid Arabidopsis thaliana solution (ATS, Lincoln et al., 1990) nutrient medium including 1 % (w/v) sucrose on vertically oriented plates under long-day conditions (16 h of light/8 h of dark) with 90 µmol m− s− photosynthetically active radiation (PAR) from white fluorescent lamps (T5 4000K) at 20 or 28°C. 5 days-old Col-0, pin1-1, and  pin4-2 seedlings carrying the DR5revp:SV40:3×GFP reporter were fixed directly with 4 % (w/v) paraformaldehyde at room temperature, washed with 1x PBS, and kept in the dark until imaging (excitation wavelength: 561 nm; emission wavelength: 571-615 nm). Columella cells including the quiescent center were determined as the fixed area through all measurements. Mean grey values were measured by using ImageJ. Representative pictures for each genotype and temperature were cropped and compiled into a single file using Gimp software to then adjust contrast and color intensity simultaneously on all pictures for publication and print.Figure 5C: Cell cycle activity in pin mutants (EdU staining)Seeds of Col-0, pin1-1, and eir1-1 were surface sterilized, rinsed with sterile water, and then imbibed and stratified for 3 days at 4°C in deionized water before sowing on solid Arabidopsis thaliana solution (ATS, Lincoln et al., 1990) nutrient medium including 1 % (w/v) sucrose. Seedlings werde cultivated on vertically oriented plates under long-day conditions (16 h of light/8 h of dark) with 90 µmol m− s− photosynthetically active radiation (PAR) from white fluorescent lamps (T5 4000K) at 20°C or 28°C. 5-Ethynyl-2'-deoxyuridine (EdU) staining was performed with the EdU Click-488 Imaging Kit (Carl-Roth) according to the manufacturer’s instruction. Briefly, 5 days-old Col-0 seedlings (at ZT1, 1 h after lights on) were immersed for 1 h in liquid ATS medium containing 10 μ EdU, and fixed in 4 % (w/v) paraformaldehyde and 0.5 % Triton X-100 for 20 min. After washing twice with 1x PBS, samples were incubated in the reaction cocktail for 30 min in the dark. The reaction cocktail was then removed, and samples were washed with 1x PBS, followed by confocal microscopy with a Zeiss LSM 780 AxioObserver (excitation wavelength: 488 nm; emission wavelength: 491-585 nm). The region of interest (root meristem) was determined with the same fixed area in all measurements, and positively stained cells were counted in this area to calculate cells per 1000 μ2.Representative pictures for each genotype and temperature were cropped and compiled into a single file using Gimp software to then adjust contrast and color intensity simultaneously on all pictures for publication and print.Figure 5D: RT-qPCR analysis of PIN expression in whole roots and root tipsCol-0 seedlings were cultivated at either 20°C or 28°C for 5 days in long-day photoperiods (16/8 h). Seedlings were harvested at Zeitgeber time (ZT) 1 and dissected by cutting off whole roots or root tips to perform expression analysis. Total RNA was extracted from 3 biological replicates using the NucleoSpin RNA Plant Kit (Macherey-Nagel). First-strand cDNA was synthesized using the PrimeScript RT Reagent Kit (Perfect Real Time) from Takara Bio. qPCR analyses were performed on an AriaMx Real-Time PCR System (Agilent) using Absolute Blue Low Rox Mix (Thermo Fisher Scientific). AT1G13320 was used as a reference gene (Czechowski et al., 2005) to calculate relative expression values (2Δt values). For primer sequences see methods.Figure 5E:Seeds of PIN2::PIN2-GFP (Luschnig et al., 1998; Muller et al., 1998) were surface sterilized, rinsed with sterile water, and then imbibed and stratified for 3 days at 4°C in deionized water before sowing on solid Arabidopsis thaliana solution (ATS, Lincoln et al., 1990) nutrient medium including 1 % (w/v) sucrose. Seedlings werde cultivated on vertically oriented plates under long-day conditions (16 h of light/8 h of dark) with 90 µmol m− s− photosynthetically active radiation (PAR) from white fluorescent lamps (T5 4000K) at 20°C or 28°C. were fixed directly with 4 % (w/v) paraformaldehyde at room temperature, washed with 1x PBS, and kept in the dark until imaging (excitation wavelength: 561 nm; emission wavelength: 571-615 nm). GFP signal intensity was measured by using areas of fixed size on basal and lateral areas in the ImageJ software, and at least ten meristem cortical cells per seedling were taken into the measurement. Representative pictures for each temperature were cropped and compiled into a single file using Gimp software to then adjust contrast and color intensity simultaneously on all pictures for publication and print.
