# Supplementary material for: Auxin‐dependent regulation of cell division rates governs root thermomorphogenesis
Source: EMBO J. 2023 Apr 18;42(11):e111926. doi: 10.15252/embj.2022111926 (PMC10233379; doi:10.15252/embj.2022111926)
Supplement: Supplementary file 9 [file EMBJ-42-e111926-s008.pdf]

THE EMBO JOURNAL

Auxin-dependent regulation of cell division rates governs root thermomorphogenesis

Haiyue Ai, Julia Bellstaedt, Kai Bartusch, Lennart Eschen-Lippold, Steve Babben, Gerd Balcke, Alain Tissier, Bettina Hause, Tonni Andersen, Carolin Delker and Marcel Quint
DOI: 10.15252/embj.2022111926

Corresponding author(s): Marcel Quint (marcel.quint@landw.uni-halle.de)

Review Timeline:

Submission Date:	21st Jun 22
Editorial Decision:	2nd Sep 22
Revision Received:	8th Sep 22
Editorial Decision:	8th Sep 22
Revision Received:	23rd Jan 23
Editorial Decision:	10th Mar 23
Revision Received:	16th Mar 23
Accepted:	21st Mar 23

Editor: William Teale

Transaction Report:

(Note: With the exception of the correction of typographical or spelling errors that could be a source of ambiguity, letters and reports are not edited. Depending on transfer agreements, referee reports obtained elsewhere may or may not be included in this compilation. Referee reports are anonymous unless the Referee chooses to sign their reports.)

1st Editorial Decision **2nd Sep 2022**

Dear Marcel,

Thank you for submitting your study, "Auxin-dependent acceleration of cell division rates regulates root growth at elevated temperature", to EMBO Journal. It was reviewed by three referees, whose reports I have attached to the bottom of this email. I have read the reports and your manuscript very carefully and have discussed them with my editorial colleagues. As you will see, the reports are not unanimously positive. My take is this: without a clear mechanistic idea of temperature sensing in the root, your study does not make a clear enough advance for our readership. Therefore at this stage, I am not able to pursue this manuscript towards publication.

I appreciate that, at its core, your work tests the hypothesis that roots use an autonomous temperature response. As reviewer 3 points out, your findings here are clearly novel, interesting and well supported by the data you present. However, I share referee 1's concerns that, without more progress into the nature of the signalling events involved, this message has become diluted by the findings of previous, more descriptive reports. If you are able to add further mechanistic insight into the molecular events of root-specific temperature sensing, I will enthusiastically consider the manuscript again. For such resubmissions, we take novelty over the original manuscript into consideration and might involve additional referee(s). If you are considering a resubmission of the paper once you have gained further mechanistic insight, please contact me in advance.

Best wishes,

William

William Teale, PhD
Editor
The EMBO Journal
w.teale@embojournal.org

****

Referee #1:

The study of Ai et al., investigates mechanisms underlying root growth adaptive response to elevated temperature. Authors perform thorough root phenotype analyses including real-time imaging to demonstrate that there is a time window, during which roots of seedlings germinating at elevated temperature respond by enhanced growth. Monitoring of detached root growth and grafting experiments support the conclusion that roots sense and respond to elevated temperature in the organ's autonomous manner. Using a set of cell cycle reporters and cell size measurements it is proposed that enhanced root growth at elevated temperatures is a result of an increased cell division rate. Analyses of the auxin-sensitive reporter and interference with auxin biosynthesis and signalling using specific inhibitors support auxin as a hormone involved in the regulation of root growth response to temperature by promoting cell division rates at the root meristem. Finally, the role of PIN auxin efflux transporters is addressed and PIN1, PIN2 and PIN4 are identified as key players adjusting auxin distribution at root meristem at elevated temperature.

The study is experimentally well executed and presented in a clear and comprehensive way. While it reveals some interesting aspects of root growth adaptation to elevated temperature, in particular providing support for a root autonomous sensing and response to temperature, most of the results rather extend/corroborate findings and current views on temperature-induced cell elongation. Thus, in my opinion, the work at this stage does not represent a major advancement in the understanding of root response to hAT. See my comments below.

1. Finding that roots respond to elevated temperature by enhanced growth has been reported in several articles, among others, the study of Yang et al 2017 provides a very detailed analysis of root growth response to a range of temperatures using Arabidopsis as a model, with a similar conclusion. Analyses performed in this study extend the previous works by using cell cycle reporters to demonstrate that more cells at the root meristem undergo division at elevated temperatures.
Specific comments:
- Author should pay attention to the terminology used when describing the root meristem phenotype - promoted cell division rate, and accelerated cell cycle (figure legend 4) seem to be used as equal terms. I believe that based on phenotype (at elevated temperature root meristem size does not significantly alter it can be speculated about the acceleration of cell cycle, but I do not see there clear experimental evidence showing that cell cycle at elevated temperature is accelerated).
- Figure 4B - the identity of markers (red and green fluorescent signals) should be explained in the figure legend

2. Authors address the question of whether roots sense and respond to elevated temperature in the organ autonomous manner

(challenging also previous findings reporting the contribution of non-organ autonomous mechanisms involving shoot thermo-sensing components such as PHY PIFs and HY5 Gaillochet et al., 2020). They elaborate on experiments performed in their lab and published previously (Bellstaedt et al., 2019) and employ also grafting experiments to corroborate previous findings about root autonomous mechanisms and excluding shoot contribution of components of shoot thermo-sensing pathway (Phy, PIFs and Hy5). This I find the most novel and important part of the whole study, which opens a key question about a sensing mechanism acting in the root.

Specific comments:
-Please note a discrepancy between the description of the experimental set-up (Figure 2B - 9 days old seedlings and material and methods (hypocotyl grafting) - stating that 7 days old seedlings were grafted
-Fig 2B according to material and methods, graph 2B shows an increase in root length after the transfer of seedlings to 20{degree sign}C or 28{degree sign}C, (which is different from graphs where the lengths of whole roots are measured)
-YHB line (Figure 2C) - should be properly described in the text and also in the figure legend (as it is not self-explanatory)

3. The role of auxin in the root response to elevated temperature has been addressed previously, (auxin reporter DII-VENUs was used to show that elevated temperature promotes auxin response at root meristem by Hanzawa et al., 2013; mutants in auxin perception pathway were tested as well (Gaillochet et al., 2020). The study provides additional support for auxin implementing different tools such as inhibitors of auxin biosynthesis and signalling to visualize and correlate auxin with promoted cell division. However, overall the results are rather confirmatory.
Specific comments:
-YUCCA8 and TAA1 auxin biosynthetic genes were identified as thermo-responsive in a shoot (both a direct target of PIF4; (Franklin et al., 2011; Sun et al.,2012). Taking into consideration that TAA1 is also expressed in the root (Stepanova et al., 2008), analyses of their expression and mutant phenotypes would inform about their role in root growth response to elevated temperature. This would not only complement experiments using kynurenine and yuccasin, but also provide additional information about the thermo-responsive expression of these genes in the root.
-Figure 5A and material and methods - please make clear whether concentrations are for Kynurenine and Yuccasin, each individual.
-Manuscript would benefit from a better explanation of why both inhibitors were used. Did the authors test them also separately (?)
-Figure 5E - Figure legend does not correspond to material and methods (1 hour versus 2-3hours treatment with EdU and auxin, PEO-IAA)

4. Analysis of mutants in PIN auxin efflux carriers is performed to explore the role of auxin transport in the root adaptation to elevated temperature, revealing PIN1, and PIN2 as positive, while PIN4 as a negative regulator. This is an interesting finding, particularly specific contributions of different PINs to the root adaptation to temperature. However, I find this set of results still rather preliminary and worth more attention in several aspects.
Specific comments:
-Root lengths are measured to characterise pin mutant phenotype (Figure 6). Taking into consideration the key role of auxin in promoting cell divisions at root meristem (as shown in Figure 5E), I would expect the same assay would be used to characterize also pin mutants. Data presented in Figure EV5 are much less informative.
-Analyses of auxin levels and auxin response (DR5, DII-Venus) in pin mutants would be another important experiment to dissect the role of individual PINs in this process.
-Expression analyses of PINs are still rather preliminary and other approaches (RT-qPCR, PIN: GUS reporters, immunolocalisation should be performed.
- It seems to be neglected that previous findings by Hanzawa et al., 2013, demonstrated the role of PIN2 (along with AUX1 auxin influx carrier) in root thermo-response. In this article PIN2 expression and polarity have been analyzed and linked with SNX1-dependent trafficking. As these results suggested that at elevated temperatures trafficking rather than the expression of PINs are targeted, it would be of interest to test whether trafficking of other PINs (PIN1 and PIN4) is affected (assay such as sensitivity to Brefeldin A used for addressing these questions could be implemented).
-Lateralization of PIN2 basal::lateral (Figure 6G) is interesting and worth attention. It has been shown, that auxin promotes lateralization of PIN2 and PIN1 so that PIN2 accumulates in the cortex lateral membranes facing towards the epidermis and PIN1 in endodermis towards central vasculature (Sauer et al., 2006).
- From the manuscript pattern of PIN2 lateralization is not obvious and should be carefully analyzed (is PIN2 enriched at lateral membranes towards epidermis or endodermis, or both?) Another interesting aspect is that while auxin promotes PIN2 lateralization, here lateralization is detected under conditions of lower auxin activity at the root meristem.

Referee #2:

I really liked some aspects of the paper - all experiments were carefully designed and executed, the data look beautiful. The manuscript was well written.

However, I have two major concerns. First, how novel it is about the independent role of HY5 in the roots given the recent report by the Huq lab: https://www.nature.com/articles/s41467-021-24018-7 - this paper highlights the distinct roles of PIF4 in the shoot and HY5 in the root. In additional, this paper shows the importance of HY5 phosphorylation in its warm-temperature-dependent accumulation in darkness. Second, the authors concluded that root thermomorphogenesis was not reugulated by phytochromes. I was puzzled by the fact that they did not see a temperature phenotype in the phytochrome mutants, which is contradictory to what was reported by Gaillochet et al. https://pubmed.ncbi.nlm.nih.gov/33144393/. One noticeable difference is that the authors used mutants in the Ler background for those experiments - i.e., all the lines in Figure 2C including phyABCBE, phyB1, and YHB. I was wondering whether the discrepancies was due to a ecotype-specific difference in temperature responses.

Referee #3:

I would first like to congratulate the authors on their manuscript. It's clear that they have put a lot of work into obtaining these results. Several of these experiments are extremely laborious and some are very technically challenging. I'm particularly impressed by the phenotypic work performed on grafted seedlings! The work presented will have a large impact on the field, and the manuscript is likely to be highly cited. The authors establish that root thermomorphogenesis is largely based on increased cell division and they go on to show that this is likely due to increased auxin in the root tip. They show important mechanistic insight into the role of PINs in controlling this process, tying together strands of evidence from the literature into a coherent model.

The authors conclusions are generally upheld by the data, but there is one major point that I feel needs attention. The authors claim that "the elevated auxin levels at high temperatures in the root tip are root-derived", and cite their data from grafting experiments. These experiments clearly shows that PIN function in the root is required for root thermomorphogenesis, but I don't think it says anything about the location of auxin synthesis. If the authors want to make this claim, I would suggest that they measure auxin levels in detached roots. At the very least, they should image the detached roots of their DR5v2 reporter. If the authors cannot perform extra experiments at this time, I would be happy to see the conclusions modified.

A more minor concern is the conclusions made about the role of shoot derived signals in root elongation. The authors state "The only line with a potential shoot-to-root effect in these experiments was the shoot thermosensory mutant phyB-9." I would argue that the fact that hy5-51 root stocks can be rescued with WT scions (Figure EV2C) implies that (in addition to local root effects), shoot-derived HY5 controls temperature-dependent root elongation.

Another minor concern is the tone in which some of the conclusions are made. Conclusions could be modified to reflect a level of uncertainty. For example, instead of 'the only reasonable explanation is...' they could say something to the effect of 'a likely explanation is that....'. The same goes for other terms such as 'obviously' and 'certainly'.

The manuscript is generally well written. The methods section is especially complete and I thank the authors for the effort they have gone to make this work reproducible. I do have some extra suggestions that could enhance the quality of the writing:

Line 70, the authors imply, but don't explicitly state why it is unlikely that root thermomorphogenesis is not dependent on light signalling.

Line 85 & 117, Gaillochet et al. (2020) argue that root temp responses are influenced by the shoot, not that they require the shoot.

Line 186, 'likely' instead of 'therefore' as this is not measured.

Fig. S4D = Fig. EV4D

Fig4. It would be helpful to explain a bit more about what the images represent (e.g. what are the green and red colours in the Cytrap lines?). Also, other colour combination are recommended for colourblind readers.

Line 360. "lack of phenotype", this could be more specific.

Line 382. I'm not sure if it is known whether adult shoots increase cell division at warm temperature. Possibly change to 'juvenile shoots'.

Line 455. "which would enable (but also require) to integrate" or "which would enable (or be required) to integrate".

The previous finding of Yang et al, that warm temps promote cell division could be given more prominence in the MS.

Appeal - Editorial Decision 8th Sep 2022

Dear Marcel,

Firstly, I'd like to thank you for following up on our editorial decision on manuscript EMBOJ-2021-111926 'Auxin-dependent acceleration of cell division rates regulates root growth at elevated temperature'. I have now had a chance to take a fresh look at the manuscript and have discussed with my editorial colleagues it again in light of your comments. Whilst I understand the reviewers' concerns, I fully see that your conclusions about temperature acting on growth via the cell cylce in a shoot-independent manner are both timely and important to the scientific community.

I have therefore decided to ask you to address the reviewers concerns in a revised version of the manuscript. In doing so, I encourage you to give your data a deeper mechanistic grounding wherever possible in order to allay the reviewers' concerns. I should add that it is The EMBO Journal policy to allow only a single major round of revision and that it is therefore important to resolve all concerns at this stage. Our usual revision time of three months is only used as a guideline and not a deadline; manuscripts frequently take longer to revise.

I would also like to point out that as a matter of policy, competing manuscripts published during this period will not be taken into consideration in our assessment of the novelty presented by your study ("scooping" protection). We have extended this 'scooping protection policy' beyond the usual three month revision timeline to cover the period required for a full revision to address the essential experimental issues. Please contact me if you see a paper with related content published elsewhere to discuss the appropriate course of action.

When preparing your letter of response to the referees' comments, please bear in mind that this will form part of the Review Process File, and will therefore be available online to the community. For more details on our Transparent Editorial Process, please visit our website: https://www.embopress.org/page/journal/14602075/authorguide#transparentprocess

Please contact me at any time during revision if you need any help or have further questions.

Thank you very much again for the opportunity to consider your work for publication. I look forward to your revision.

Best regards,

William

William Teale, Ph.D.
Editor
The EMBO Journal

When submitting your revised manuscript, please carefully review the instructions below and include the following items:

1) a .docx formatted version of the manuscript text (including legends for main figures, EV figures and tables). Please make sure that the changes are highlighted to be clearly visible.

2) individual production quality figure files as .eps, .tif, .jpg (one file per figure).

3) a .docx formatted letter INCLUDING the reviewers' reports and your detailed point-by-point response to their comments. As part of the EMBO Press transparent editorial process, the point-by-point response is part of the Review Process File (RPF), which will be published alongside your paper.

4) a complete author checklist, which you can download from our author guidelines (https://wol-prod-cdn.literatumonline.com/pb-assets/embo-site/Author Checklist%20-%20EMBO%20J-1561436015657.xlsx). Please insert information in the checklist that is also reflected in the manuscript. The completed author checklist will also be part of the RPF.

5) Please note that all corresponding authors are required to supply an ORCID ID for their name upon submission of a revised manuscript.

6) We require a 'Data Availability' section after the Materials and Methods. Before submitting your revision, primary datasets produced in this study need to be deposited in an appropriate public database, and the accession numbers and database listed under 'Data Availability'. Please remember to provide a reviewer password if the datasets are not yet public (see https://www.embopress.org/page/journal/14602075/authorguide#datadeposition). If no data deposition in external databases is needed for this paper, please then state in this section: This study includes no data deposited in external repositories. Note that

the Data Availability Section is restricted to new primary data that are part of this study.

Note - All links should resolve to a page where the data can be accessed.

7) When assembling figures, please refer to our figure preparation guideline in order to ensure proper formatting and readability in print as well as on screen:
http://bit.ly/EMBOPressFigurePreparationGuideline

Please remember: Digital image enhancement is acceptable practice, as long as it accurately represents the original data and conforms to community standards. If a figure has been subjected to significant electronic manipulation, this must be noted in the figure legend or in the 'Materials and Methods' section. The editors reserve the right to request original versions of figures and the original images that were used to assemble the figure.

8) For data quantification: please specify the name of the statistical test used to generate error bars and P values, the number (n) of independent experiments (specify technical or biological replicates) underlying each data point and the test used to calculate p-values in each figure legend. The figure legends should contain a basic description of n, P and the test applied. Graphs must include a description of the bars and the error bars (s.d., s.e.m.).

9) We would also encourage you to include the source data for figure panels that show essential data. Numerical data can be provided as individual .xls or .csv files (including a tab describing the data). For 'blots' or microscopy, uncropped images should be submitted (using a zip archive or a single pdf per main figure if multiple images need to be supplied for one panel). Additional information on source data and instruction on how to label the files are available at .

10) We replaced Supplementary Information with Expanded View (EV) Figures and Tables that are collapsible/expandable online (see examples in https://www.embopress.org/doi/10.15252/embj.201695874). A maximum of 5 EV Figures can be typeset. EV Figures should be cited as 'Figure EV1, Figure EV2" etc. in the text and their respective legends should be included in the main text after the legends of regular figures.

- For the figures that you do NOT wish to display as Expanded View figures, they should be bundled together with their legends in a single PDF file called *Appendix*, which should start with a short Table of Content. Appendix figures should be referred to in the main text as: "Appendix Figure S1, Appendix Figure S2" etc. See detailed instructions regarding expanded view here: .

- Additional Tables/Datasets should be labelled and referred to as Table EV1, Dataset EV1, etc. Legends have to be provided in a separate tab in case of .xls files. Alternatively, the legend can be supplied as a separate text file (README) and zipped together with the Table/Dataset file.

11) Our journal encourages inclusion of *data citations in the reference list* to directly cite datasets that were re-used and obtained from public databases. Data citations in the article text are distinct from normal bibliographical citations and should directly link to the database records from which the data can be accessed. In the main text, data citations are formatted as follows: "Data ref: Smith et al, 2001" or "Data ref: NCBI Sequence Read Archive PRJNA342805, 2017". In the Reference list, data citations must be labeled with "[DATASET]". A data reference must provide the database name, accession number/identifiers and a resolvable link to the landing page from which the data can be accessed at the end of the reference. Further instructions are available at .

The EMBO Journal
contact@embojournal.org

Content alerts: embopress.org/alertsfeeds
Follow us on Twitter: twitter.com/embojournal

Instructions for preparing your revised manuscript:

Please make sure you upload a letter of response to the referees' comments together with the revised manuscript.

Please also check that the title and abstract of the manuscript are brief, yet explicit, even to non-specialists.

When assembling figures, please refer to our figure preparation guideline in order to ensure proper formatting and readability in print as well as on screen:
https://bit.ly/EMBOPressFigurePreparationGuideline
See also guidelines for figure legends: https://www.embopress.org/page/journal/14602075/authorguide#figureformat

IMPORTANT: When you send the revision we will require
- a point-by-point response to the referees' comments, with a detailed description of the changes made (as a word file).
- a word file of the manuscript text.
- individual production quality figure files (one file per figure)
- a complete author checklist, which you can download from our author guidelines (https://www.embopress.org/page/journal/14602075/authorguide).
- Expanded View files (replacing Supplementary Information)
Please see out instructions to authors
https://www.embopress.org/page/journal/14602075/authorguide#expandedview

Please remember: Digital image enhancement is acceptable practice, as long as it accurately represents the original data and conforms to community standards. If a figure has been subjected to significant electronic manipulation, this must be noted in the figure legend or in the 'Materials and Methods' section. The editors reserve the right to request original versions of figures and the original images that were used to assemble the figure.

Further information is available in our Guide For Authors: https://www.embopress.org/page/journal/14602075/authorguide

We realize that it is difficult to revise to a specific deadline. In the interest of protecting the conceptual advance provided by the work, we recommend a revision within 3 months (7th Dec 2022). Please discuss the revision progress ahead of this time with the editor if you require more time to complete the revisions. Use the link below to submit your revision:

https://emboj.msubmit.net/cgi-bin/main.plex

Halle (Saale), Jan 23, 2023

Regarding revision of EMBOJ-2022-111926R-Q

Dear Reviewers,
Thank you very much for sharing so many constructive comments on our manuscript on root thermomorphogenesis in *A. thaliana*. We are happy to have been invited to submit a revised version of the manuscript by the editor. We furthermore greatly appreciate that you stressed the thoroughness of our experimental approaches by stating that '*the study is experimentally well executed and presented in a clear and comprehensive way*', and that '*all experiments were carefully designed and executed, the data look beautiful [… and] the manuscript was well written.*' However, your opinions were obviously not unanimously supportive of publication of our manuscript in *EMBO J* at this stage. For example, while R3 anticipated that '*the work presented will have a large impact on the field, and the manuscript is likely to be highly cited*', R1's opinion was that '*the work at this stage does not represent a major advancement in the understanding of root response to hAT*'.

The judgement of the potential impact of our study on plant biology likely depends on the perspective the reader takes. When you expect depth and detail in specific aspects of root temperature signaling, you might be inclined to perceive the impact of our study as lower than what is to be expected from a paper published in *EMBO J*. In contrast, from a more global perspective, we are convinced that our work represents a major advance in understanding the larger context of the molecular mechanisms roots employ to translate elevated temperature stimuli to primary root growth in *A. thaliana*. Hence, we agree that several bits of the information we present have been described previously, and sometimes in more detail. For example, there are several studies showing that temperature affects the regulation of several aspects of auxin biology (e.g., Hanzawa *et al*., 2013; Wang *et al*., 2016; Feraru *et al*., 2019). And while there are conflicting reports about the effect of temperature on cell elongation and cell division (see new Introduction and Discussion sections and responses below), it has also been described a while ago that high temperature affects the cell cycle across plant species (e.g., Grif and Ivanov, 2002). However, each of these (and several other) studies addresses only an isolated aspect of temperature effects on various processes in plant roots, in case of genetic approaches sometimes backed up by rather weak root growth phenotypes. While we also value the trend of providing ever more detail on a specific aspect with highly advanced technological approaches, we certainly did not aim to illuminate one of these details. In fact, we are not aware of any paper that connects the dots and presents an experimentally well supported and comprehensive model for the major mechanism that regulates temperature-induced root growth.

As such, our approach was to clearly focus on players with severe, but conditional (temperature!), root growth phenotypes, to connect the above mentioned dots, and to provide a synthesis of various temperature effects on root growth that allows us to generate a comprehensive mechanistic model ('*tying together strands of evidence from the literature into a coherent model*' - R3). Along these lines, we show that the general mechanism of temperature-induced root growth is to be sought at the level of cell division rather than cell elongation. We provide a molecular mechanism across all levels of primary signaling starting with spatial aspects of root thermosensing, followed by warmth-induced auxin biosynthesis in the root tip, increased auxin flow through the root apical meristem involving temperature-sensitive reorientation of PIN2 auxin efflux carriers, which then triggers the acceleration of cell division rates, ultimately resulting in extended primary root growth at elevated temperatures. Taken together, we are in agreement with R3 and firmly believe that our study has the potential to indeed serve as a new baseline for future studies of root thermomorphogenesis.

We have picked up the numerous excellent comments from you and added novel experimental data to substantiate our model and address several of the mentioned criticisms. This includes the following:

- Substantiating temperature-induced local auxin biosynthesis in the root:
  - Temperature response root growth assays with NPA blocking shoot-root auxin flow

- Temperature-induced activation of DR5 reporter activity in the root apical meristems of wt *vs. wei8-1,tar1-1* auxin biosynthesis mutants

- Additional general auxin data:
  - Dose-response curve of seedling root growth at different temperatures on increasing concentrations of the auxin transport inhibitor NPA
  - Temperature response root growth assays of auxin biosynthesis and signaling mutants
  - Temperature-responsive transcriptional analysis of *PIN*s in whole roots and root tips

- Substantiating the role of PINs in temperature-induced cell division:
  - EdU staining of *pin1 and pin2* alleles in response to temperature
  - Temperature-induced activation of DR5 reporter activity in wt vs. *pin* mutant backgrounds

- Substantiating the integral role of cell division in temperature-induced root growth:
  - Temperature response root growth assays of *e2f* mutants as central regulators of cell cycle entry

Furthermore, we have rewritten parts of the *Introduction* section to better point out the wealth of classic and recent studies that provide the above mentioned isolated pieces of information which we aimed to connect in our study. We sincerely hope that the revised version of the manuscript is now convincing enough for all reviewers and the editorial board to support publication in *EMBO J*. Please find a point-by-point response to your comments below.

Sincerely,

Marcel Quint and co-authors

Point-by-point response

Referee #1:

The study of Ai et al., investigates mechanisms underlying root growth adaptive response to elevated temperature. Authors perform thorough root phenotype analyses including real-time imaging to demonstrate that there is a time window, during which roots of seedlings germinating at elevated temperature respond by enhanced growth. Monitoring of detached root growth and grafting experiments support the conclusion that roots sense and respond to elevated temperature in the organ's autonomous manner. Using a set of cell cycle reporters and cell size measurements it is proposed that enhanced root growth at elevated temperatures is a result of an increased cell division rate. Analyses of the auxin-sensitive reporter and interference with auxin biosynthesis and signalling using specific inhibitors support auxin as a hormone involved in the regulation of root growth response to temperature by promoting cell division rates at the root meristem. Finally, the role of PIN auxin efflux transporters is addressed and PIN1, PIN2 and PIN4 are identified as key players adjusting auxin distribution at root meristem at elevated temperature.

The study is experimentally well executed and presented in a clear and comprehensive way. While it reveals some interesting aspects of root growth adaptation to elevated temperature, in particular providing support for a root autonomous sensing and response to temperature, most of the results rather extend/corroborate findings and current views on temperature-induced cell elongation. Thus, in my opinion, the work at this stage does not represent a major advancement in the understanding of root response to hAT. See my comments below.

Response: We do appreciate that the thorough execution of the experiments is acknowledged. Regarding the reviewer's perception of the lack of advancement in the understanding of root responses to elevated temperature stimuli we are, not surprisingly, in disagreement. As pointed out in the general address to the reviewers above, we are not aware of a single paper that provides a comprehensive mechanistic model explaining temperature-induced root elongation from sensing via signaling towards the cellular process(es) that ultimately execute(s) root growth. To provide

such a global mechanistic model was therefore the aim of our study. Naturally, we based our approach on a number of previously published studies that illuminated distinct specific aspects of root responses to elevated temperatures. The challenge of our study was to connect the dots of existing information to derive a larger picture. Along the way, we generated and reported a number of novel findings including independency of thermosensing from the shoot, auxin-dependent acceleration of cell division rates in high temperature, and the identification of the latter (cell division) as the major driver of temperature-induced root elongation. We have backed up these novel findings with solid phenotypes of loss-of-function mutants which are largely conditional, strongly favoring temperature specificity of these processes over pleiotropic responses. Unfortunately, especially clear but conditional mutant phenotypes have been largely lacking in the root thermomorphogenesis literature so far. We understand that this reviewer would prefer that we provide more depth and detail on our novel findings (e.g., more detail on sensing or more detail on auxin-regulation of the cell cycle, etc.). As obvious from the resubmitted version of the manuscript and the extent of the additional data, we have tried our best to realize this, while still keeping the focus of the manuscript on the comprehensive mechanism rather than turning our study into a more specific one. The main advance we see in our work is that we are able to add novel data to the still very young root thermomorphogenesis field that serve as missing links to connect the previously published isolated aspects of root thermomorphogenesis. We therefore decided against a detailed analysis of one of these novel aspects to instead focus on a larger context. As a result, we provide the - in our opinion - first comprehensive model of root temperature signaling, which we believe is a significant finding many studies will be able to build on in the future. In any case, this reviewer provided numerous constructive suggestions on ways to substantiate our data. We followed the vast majority of suggestions, performed a number of additional experiments and added extensive novel data to the revised version of the manuscript, which further strengthen the model we proposed.

1. Finding that roots respond to elevated temperature by enhanced growth has been reported in several articles, among others, the study of Yang et al 2017 provides a very detailed analysis of root growth response to a range of temperatures using Arabidopsis as a model, with a similar conclusion. Analyses performed in this study extend the previous works by using cell cycle reporters to demonstrate that more cells at the root meristem undergo division at elevated temperatures.

Response: Agreed. We have now elaborated in more detail on the findings from the Yang study from Tobias Baskin's lab (Plant Cell Environ 40, 264-276) in the *Discussion* section of the initially submitted manuscript. However, we did obviously not do this in enough detail, because Yang et al. did not come to exactly the same conclusions as we and also others in the literature did. The Yang study aimed to disentangle the different parameters regulating root growth in response to changing ambient temperatures in Arabidopsis on a kinematic level. While they could confirm several of the previously reported observations like a temperature responsive gradual increase of total root length, elemental elongation rate, velocity, cell division rates, transit of cells through the different root zones, and an invariant elongation zone length in response to temperature, they also reported results that are in partial contrast to the literature and our own data. Interestingly, Yang et al. (2017), as well as Feraru et al. (2019, PNAS 116, 3893-3898), found a negative effect of increasing temperatures on meristem length, which was compensated by a shorter cell cycle duration and cell division rates (Yang et al., 2017). Integration of all these processes resulted in an invariant final cell flux (i.e. total rate of cell production) across a temperature range between 15°C and 25°C. This means that according to Yang et al. (2017) increased root growth at high temperatures also depends on promotion of cell elongation. This contrasts with previous reports from maize roots (Silk, 1992, Int J Plant Sci 153, S49-S58) and leaves (Ban-Haj-Salah and Tardieu, 1995, Plant Physiol 109, 861-870), and also with our own data presented in this manuscript, all of which report(ed) rather stable cell length but increased cell flux and/or total cell production across a temperature range. Obviously, these partially adverse findings (we did also see an increase in cell elongation, see new Fig. EV3E) lead to somewhat different conclusions regarding the primary driver of temperature-promoted root growth being either cell elongation or cell division. It remains to be seen whether this is due to differences in cultivation methods, as suggested by Nagel et al. (2009, Funct Plant Biol 36, 947-959) and Yang et al. (2017), or other technical specificities.

In any case, our own data support the predominant role of cell cycle acceleration as the process that drives elevated temperature promoted root growth. And, as this reviewer mentions, we substantiated these observations with a number of cell cycle reporters showing that more cells undergo division at high temperatures in the root apical meristem. Later in the manuscript we also showed that this process depends on increased auxin levels and an intact polar auxin transport system. In the revised version of the manuscript we now also show defective (and conditional) temperature responsive root growth data of *e2f* mutants. Being mutated in central regulators of cell cycle entry (E2F transcription factors, Fig. EV3F revised version of the manuscript), this provides solid genetic evidence for the cell cycle's role in this process. While it is impossible to include a full report on the various studies from the last 50 years that addressed the effect of temperature on all sorts of root growth related processes including cell division rates, we realized that we need to do a better job in explaining the historic background and also pointing out the specific aspects of knowhow that served as the basis for the comprehensive model we are proposing in our manuscript. We have therefore rewritten parts of the *Introduction* and *Discussion* sections to account for this.

Specific comments:

- Author should pay attention to the terminology used when describing the root meristem phenotype - promoted cell division rate, and accelerated cell cycle (figure legend 4) seem to be used as equal terms. I believe that based on phenotype (at elevated temperature root meristem size does not significantly alter it can be speculated about the acceleration of cell cycle, but I do not see there clear experimental evidence showing that cell cycle at elevated temperature is accelerated).

Response: Agreed. We have edited the terminology used throughout the manuscript including the title accordingly.

- Figure 4B - the identity of markers (red and green fluorescent signals) should be explained in the figure legend

Response: This figure and the data have been omitted from the revised version of the manuscript.

2. Authors address the question of whether roots sense and respond to elevated temperature in the organ autonomous manner (challenging also previous findings reporting the contribution of non-organ autonomous mechanisms involving shoot thermo-sensing components such as PHY PIFs and HY5 Gaillochet et al., 2020). They elaborate on experiments performed in their lab and published previously (Bellstaedt et al., 2019) and employ also grafting experiments to corroborate previous findings about root autonomous mechanisms and excluding shoot contribution of components of shoot thermo-sensing pathway (Phy, PIFs and Hy5). This I find the most novel and important part of the whole study, which opens a key question about a sensing mechanism acting in the root.

Specific comments:

-Please note a discrepancy between the description of the experimental set-up (Figure 2B - 9 days old seedlings and material and methods (hypocotyl grafting) - stating that 7 days old seedlings were grafted

Response: Thanks a lot for pointing this mistake out. Changed accordingly.

-Fig 2B according to material and methods, graph 2B shows an increase in root length after the transfer of seedlings to 20{degree sign}C or 28{degree sign}C, (which is different from graphs where the lengths of whole roots are measured)

Response: Thank you for pointing this out. We have measured only the growth after recovery (not the whole root). The y-axis description of the graph has been changed to 'Root growth after graft recovery [mm]'.

-YHB line (Figure 2C) - should be properly described in the text and also in the figure legend (as it is not self-explanatory)

Response: This figure has been moved to the extended version figures. The corresponding data are now included in Fig. EV2C. We now also include a description and reference of the YHB line in the figure legend. To avoid expanding this part of the manuscript, which displays only an introductory side aspect of our study, we, however, prefer to refrain from adding this also to the main text. The main message (various shoot temperature signaling mutants still respond to temperature in terms of root growth) is hopefully clear also without going into the details of this specific mutant line.

3. The role of auxin in the root response to elevated temperature has been addressed previously, (auxin reporter DII-VENUs was used to show that elevated temperature promotes auxin response at root meristem by Hanzawa et al., 2013; mutants in auxin perception pathway were tested as well (Gaillochet et al., 2020). The study provides additional support for auxin implementing different tools such as inhibitors of auxin biosynthesis and signalling to visualize and correlate auxin with promoted cell division. However, overall the results are rather confirmatory.

Response: Partially agreed, partially disagreed. Of course we need to show that the basic behavior of auxin reporters is the same as in previous studies, which we do and acknowledge as confirmatory in the manuscript. However, while the mentioned studies do not continue to ask what exactly this enhanced auxin reporter activity causes, we show in the following experiments what these elevated levels of auxin itself (which is also new) and as a consequence also auxin response activity most likely do in the context of temperature responses in the root: they connect high temperature with increased rates of cell division in the root apical meristem (e.g., Fig. 4G, Fig. 5C revised version of the manuscript). Instead of regarding this as overall confirmatory, our follow-up experiments actually add the molecular mechanism in the context of temperature response to the previously rather descriptive use of auxin reporter activities, which we see as an important advance of our current understanding.

Specific comments:
-YUCCA8 and TAA1 auxin biosynthetic genes were identified as thermo-responsive in a shoot (both a direct target of PIF4; (Franklin et al., 2011; Sun et al.,2012). Taking into consideration that TAA1 is also expressed in the root (Stepanova et al., 2008), analyses of their expression and mutant phenotypes would inform about their role in root growth response to elevated temperature. This would not only complement experiments using kynurenine and yuccasin, but also provide additional information about the thermo-responsive expression of these genes in the root.

Response: Thank you for the suggestion. We performed additional root growth and DR5 reporter assays with *wei8-1 tar1-1 [DR5NLS]* double mutants. While their root growth phenotype was rather moderate (new Fig. EV4A; however, *yucQ* mutants had a severe phenotype), DR5NLS reporter assays show that *de novo* auxin biosynthesis via TAA1/WEI8 and TAR1 is active in the root apical meristem in response to elevated ambient temperatures, therefore supporting our previous conclusions. The new data have been included in the revised version of the manuscript as Fig. 4C. Furthermore, to complement the inhibitor dose response data (kyn+yuc for biosynthesis, PEO-IAA for signaling), we add similar dose response data for the auxin transport inhibitor NPA, showing not only inhibition of auxin biosynthesis or signaling, but also of auxin flow likewise blocks the growth promoting temperature effect (new Fig. 4E).

-Figure 5A and material and methods - please make clear whether concentrations are for Kynurenine and Yuccasin, each individual.

Response: Agreed and description included.

-Manuscript would benefit from a better explanation of why both inhibitors were used. Did the authors test them also separately (?)

Response: Auxin biosynthesis is more effectively inhibited when both inhibitors are used simultaneously. We have used the combination of both successfully in the past for the inhibition of

hypocotyl growth (Ibanez et al., 2018, Current Biology 28, 303-310). A corresponding explanation including the reference has been added to the main text.

-Figure 5E - Figure legend does not correspond to material and methods (1 hour versus 2-3hours treatment with EdU and auxin, PEO-IAA)

Response: Thank you for pointing this mistake out. We have corrected this in the revised version of the manuscript.

4. Analysis of mutants in PIN auxin efflux carriers is performed to explore the role of auxin transport in the root adaptation to elevated temperature, revealing PIN1, and PIN2 as positive, while PIN4 as a negative regulator. This is an interesting finding, particularly specific contributions of different PINs to the root adaptation to temperature. However, I find this set of results still rather preliminary and worth more attention in several aspects.
Specific comments:
-Root lengths are measured to characterise pin mutant phenotype (Figure 6). Taking into consideration the key role of auxin in promoting cell divisions at root meristem (as shown in Figure 5E), I would expect the same assay would be used to characterize also pin mutants. Data presented in Figure EV5 are much less informative.

Response: Reviewer 1 is of course correct. We have now performed EdU staining experiments in *pin* loss-of-function backgrounds and show the new data in Fig. 5C. While the wt responds with increased staining to high temperatures (and therefore an increased number of dividing cells), neither *pin1-1* nor *eir1-1* mutants respond to elevated temperatures in terms of staining patterns. These data complement and substantiate the previous root growth data and cellular measurements and support the original conclusion.
The role of PIN4 may be rather complex in this context. As hyperelongation of *pin4-2* roots at elevated temperature is most likely due to an increased meristem size (Fig. EV5A-B) and our EdU staining data are inconclusive (and therefore not shown), we do not yet understand this mechanism well enough to report it.

-Analyses of auxin levels and auxin response (DR5, DII-Venus) in pin mutants would be another important experiment to dissect the role of individual PINs in this process.

Response: Agreed. We have tried to get our hands on *pin* loss-of-function lines carrying DR5 reporters, which was more difficult than expected. While we were successful for *pin1-1* and *pin4-2*, the only *pin2* allele with a DR5 reporter we could get from colleagues (we asked Friml, Kleine-Vehn, Luschnig, and Robert-Boissivon labs, all of whom are always very helpful) was *eir1-4* (we used *eir1-1* for the other assays), which is apparently a rather weak allele. It did not show a root growth phenotype and was therefore not informative for these analyses. The phenotype of *pin1-1* (no DR5 response to high temperature) was in line with their root growth and EdU phenotypes. DR5 activity of *pin4-2* mutants was a) responsive to temperature, and b) higher than wt at both temperatures. While this pattern is not conditional, it generally fits with hyperelongation of *pin4-2* roots. Together, these data further substantiate the proposed model and are now included in Fig. 5B.

-Expression analyses of PINs are still rather preliminary and other approaches (RT-qPCR, PIN: GUS reporters, immunolocalisation should be performed.

Response: As suggested here and in addition to the PIN-GFP data we are already showing, we have performed qRT-PCR analyses of a full range of root expressed auxin related genes (biosynthesis, transport, signaling —> ∑16) in whole roots and root tips only of seedlings grown at different temperatures. The bottom line is, these genes do not seem to be particularly temperature responsive, suggesting that temperature sensitivity is rather independent of transcriptional regulation of genes encoding proteins with functions in auxin biosynthesis, transport and perception. The qRT-PCR data of *PIN1-4* have been added to the manuscript as Fig. 5D.

- It seems to be neglected that previous findings by Hanzawa et al., 2013, demonstrated the role of PIN2 (along with AUX1 auxin influx carrier) in root thermo-response. In this article PIN2

expression and polarity have been analyzed and linked with SNX1-dependent trafficking. As these results suggested that at elevated temperatures trafficking rather than the expression of PINs are targeted, it would be of interest to test whether trafficking of other PINs (PIN1 and PIN4) is affected (assay such as sensitivity to Brefeldin A used for addressing these questions could be implemented).

Response: While we mentioned the Hanzawa study in the manuscript, the reviewer is right in noting that we should have invested more to test the connection between this study and ours. We have now pointed out the complementarity of Hanzawa et al.'s observations and our data at several additional points in the manuscript. Furthermore, trafficking is certainly an interesting aspect to analyze with regard to getting to the bottom of how temperature regulates PIN2 and other auxin transporters. However, we believe that this together with a number of additional aspects will be a project in itself and would exceed the aim of this study to provide the missing links between the available pieces of information to derive a model for root thermomorphogenesis signaling.

-Lateralization of PIN2 basal::lateral (Figure 6G) is interesting and worth attention. It has been shown, that auxin promotes lateralization of PIN2 and PIN1 so that PIN2 accumulates in the cortex lateral membranes facing towards the epidermis and PIN1 in endodermis towards central vasculature (Sauer et al., 2006).
- From the manuscript pattern of PIN2 lateralization is not obvious and should be carefully analyzed (is PIN2 enriched at lateral membranes towards epidermis or endodermis, or both?) Another interesting aspect is that while auxin promotes PIN2 lateralization, here lateralization is detected under conditions of lower auxin activity at the root meristem.

Response: This is a good point we weren't aware of. Sauer *et al.* (Genes Dev 2006) described that exogenous application of IAA or NAA resulted in cortical PIN2 shifting towards the lateral membrane facing the epidermis. In fact, we did not see the same response in response to temperature. In elevated temperatures, we do also have high auxin levels, but apparently a different outcome with a) lateral PIN2 shifting to the apical (lower) membrane, and b) we do not observe a specific orientation of lateral PIN2-GFP signal. We apologize for the not always optimal quality of our confocal microscopy images. We are not an imaging lab. However, we thoroughly quantified the confocal images we took, repeated these experiments at least three times (as all other experiments performed in our lab) and can guarantee for the reproducibility of the data presented. A possible explanation for the differences between Sauer *et al.* (2006) and our study may be sought on several levels: a) in the concentrations of auxin that are influencing the system in the experiments. While our temperature induced high auxin levels are within the physiological range, the concentrations applied in the study from the Benkova lab likely exceed this by far. Possibly, this may affect also the localization of the fusion proteins. b) The length of the treatment: While Sauer *et al*., treated for 4 and 12 hrs with auxin, our seedlings grew from day 1 on at different temperatures and therefore likely also in different auxin levels in the root tip. As we see in the PIN-GFP analyses, transient treatments differ from continuous treatments in high temperature. We may have the same effect here. We have added this to the *Discussion* section of the revised version of the manuscript, but admit to have kept it very short. In any case, together with the observations of Hanzawa *et al*. (2013) who showed a shift from the lateral to the basal (upper) membrane in epidermal cells upon high temperature treatment, this suggests an increased auxin flow through the root apical meristem. We have now made this more clear in the manuscript by slightly rephrasing the corresponding parts in the text.

Referee #2:

I really liked some aspects of the paper - all experiments were carefully designed and executed, the data look beautiful. The manuscript was well written.

Response: We greatly appreciate this evaluation.

However, I have two major concerns. First, how novel it is about the independent role of HY5 in the roots given the recent report by the Huq lab: https://www.nature.com/articles/s41467-021-24018-7 - this paper highlights the distinct roles of PIF4 in the shoot and HY5 in the root. In additional, this

paper shows the importance of HY5 phosphorylation in its warm-temperature-dependent accumulation in darkness.

Response: The reviewer is absolutely correct in that the *hy5* root growth data (Fig. 2d) were more or less confirmatory of the paper from the Huq lab, which we had acknowledged several times already in the initially submitted version of the manuscript. The reason why we included these data in the manuscript was that we needed to show that the majority of shoot thermomorphogenesis mutants have either no or only weak root growth effects, suggesting a different signaling pathway for root thermomorphogenesis. What we add to the Huq paper, which entirely focused on HY5, are the micrografting data displayed in supplemental Fig. EV2c. However, the HY5 data play only a secondary role in our study and we do not at all go into detail here. Hence, the novelty of our study is independent of any HY5 related data. For the revised version of the manuscript we will move the *hy5* mutant data to the supplemental figures.

Second, the authors concluded that root thermomorphogenesis was not reugulated by phytochromes. I was puzzled by the fact that they did not see a temperature phenotype in the phytochrome mutants, which is contradictory to what was reported by Gaillochet et al. https://pubmed.ncbi.nlm.nih.gov/33144393/. One noticeable difference is that the authors used mutants in the Ler background for those experiments - i.e., all the lines in Figure 2C including phyABCBE, phyB1, and YHB. I was wondering whether the discrepancies was due to a ecotype-specific difference in temperature responses.

Response: Indeed, our phytochrome (and other shoot-root communication) data are in contradiction to the data from Gaillochet et al. (2020). However, we discuss this at length in the manuscript and substantiate our conclusion (= phytochromes play only a minor role in root thermomorphogenesis) by the micrografting assays shown in Fig. EV2d (now Fig. 2C in the revised version of the manuscript). Here, '*graft combinations including phyB-9 shoots displayed significantly shorter roots at high temperature when compared to graft combinations with wild type shoots. However, these grafting combinations were still able to respond to the temperature stimulus, suggesting a rather minor role for shoot-localized or -derived phyB in this process.*' (l. 140-145 in the initially submitted manuscript). Furthermore, Jorge Casal's lab (who discovered phyBs thermosensory role in shoots together with Phil Wigge's lab) has recently published a letter in *New Phytologist* where they convincingly showed that none of the to date discovered shoot thermosensors (phyB, ELF3, PIF7) act as thermosensors in the primary root growth response to temperature (Belén Borniego et al., 2022, *New Phytol* 236, 9-14), confirming our conclusions. Based on this, we would argue against an accession specific effect.

Referee #3:

I would first like to congratulate the authors on their manuscript. It's clear that they have put a lot of work into obtaining these results. Several of these experiments are extremely laborious and some are very technically challenging. I'm particularly impressed by the phenotypic work performed on grafted seedlings! The work presented will have a large impact on the field, and the manuscript is likely to be highly cited. The authors establish that root thermomorphogenesis is largely based on increased cell division and they go on to show that this is likely due to increased auxin in the root tip. They show important mechanistic insight into the role of PINs in controlling this process, tying together strands of evidence from the literature into a coherent model.

The authors conclusions are generally upheld by the data, but there is one major point that I feel needs attention. The authors claim that "the elevated auxin levels at high temperatures in the root tip are root-derived", and cite their data from grafting experiments. These experiments clearly shows that PIN function in the root is required for root thermomorphogenesis, but I don't think it says anything about the location of auxin synthesis. If the authors want to make this claim, I would suggest that they measure auxin levels in detached roots. At the very least, they should image the detached roots of their DR5v2 reporter. If the authors cannot perform extra experiments at this time, I would be happy to see the conclusions modified.

Response: This is a very good point. Nonetheless, we do not think that IAA measurements or DR5 imaging in detached roots are the best approach to show this because these experiments would 'only' be able to show that auxin levels or response activities, respectively, increase in elevated temperatures. The increase might, however, also be caused by wounding responses, which are also increased in high temperatures as we could show in a recent collaborative study with Charles Melnyk's lab (Serivichyaswat et al., 2022, Development 149, dev200079). A less invasive way to substantiate our conclusion would probably be to block shoot-to-root auxin transport in intact seedlings. We therefore performed a root growth assay in the presence of the auxin transport inhibitor NPA applied specifically to the shoot-root junction. Blocking auxin transport at the shoot-root junction had no effect on the root growth response, supporting our hypothesis of temperature-induced local auxin biosynthesis in the root. These novel data are now displayed in Fig. 4D in the revised version of the manuscript. They are complemented by additional new data on DR5 reporter activity in *wei8-1 tar1-1* double mutants (Fig. 4C) and *wei8-1 tar1-1* as well as *yucQ* mutant growth phenotypes (Fig. EV4A), all of which further support our model.

A more minor concern is the conclusions made about the role of shoot derived signals in root elongation. The authors state "The only line with a potential shoot-to-root effect in these experiments was the shoot thermosensory mutant phyB-9." I would argue that the fact that hy5-51 root stocks can be rescued with WT scions (Figure EV2C) implies that (in addition to local root effects), shoot-derived HY5 controls temperature-dependent root elongation.

Response: We agree that this may be a bit difficult to interpret. In Fig. EV2c we showed that '*hy5-51 mutant shoots on wild type rootstocks behaved like wild type, arguing against a role for shoot localized or shoot-derived HY5 in root thermomorphogenesis*' (l. 135-136 in the originally submitted version of the manuscript). And we stand by this conclusion based on this piece of data. Whether *hy5-51* root stocks are rescued with wt scions, as the reviewer suggests, is - in our opinion - very difficult to say because the *hy5*/*hy5* self grafts are shorter at both temperatures. So it seems that wt scions on *hy5-51* rootstocks (wt/*hy5*) result in increased root growth independent of temperature. In any case, these are very delicate experiments, but based on what we observed, the most parsimonious interpretation is that HY5, probably derived from the shoot, does play a role in root growth, but in a temperature-independent context. We have adjusted this part in the manuscript accordingly.

Another minor concern is the tone in which some of the conclusions are made. Conclusions could be modified to reflect a level of uncertainty. For example, instead of 'the only reasonable explanation is...' they could say something to the effect of 'a likely explanation is that....'. The same goes for other terms such as 'obviously' and 'certainly'.

Response: Agreed and changed accordingly.

The manuscript is generally well written. The methods section is especially complete and I thank the authors for the effort they have gone to make this work reproducible. I do have some extra suggestions that could enhance the quality of the writing:

Line 70, the authors imply, but don't explicitly state why it is unlikely that root thermomorphogenesis is not dependent on light signalling.

Response: We are not quite sure what the reviewer suggests to change here. Although quite reasonable, it is only a hypothesis that thermosensors that depend on light activation play a rather minor role in below-ground roots. We would therefore prefer to keep this rather implicative phrasing.

Line 85 & 117, Gaillochet et al. (2020) argue that root temp responses are influenced by the shoot, not that they require the shoot.

Response: Disagreed. Gaillochet et al. (2020) explicitly state for example in the abstract of their paper that '*a <u>shoot</u> signaling module that includes HY5, the phytochromes and the PIFs exerts a <u>central function</u> in coupling these growth responses and maintaining auxin levels in the root*.' In our understanding 'central function' is much closer to 'required' than to 'influenced'. In the *Discussion* section we explicitly state that we do not rule out a minor role for shoot-root communication: our

data '*favor[s] a scenario in which roots are to be regarded as autonomous systems that can independently sense and respond to temperature cues. This does not rule out the presence of temperature-sensitive shoot-to-root communication, possibly involving phyB (Fig. EV2D), but renders it non-essential for temperature-induced root elongation*' (l. 364-367 in the initially submitted version of the manuscript). We feel that this phrasing provides a balanced interpretation of our data including the contradiction to the conclusions from the Gaillochet et al. study, and would prefer to keep it as is.

Line 186, 'likely' instead of 'therefore' as this is not measured.

Response: Changed as suggested.

Fig. S4D = Fig. EV4D

Response: Thank you for pointing this out. Changed accordingly.

Fig4. It would be helpful to explain a bit more about what the images represent (e.g. what are the green and red colours in the Cytrap lines?). Also, other colour combination are recommended for colourblind readers.

Response: As it should suffice to display cells in early (EdU) and late (DAPI) phases of the cell cycle, we now exclude the cytrap data from the revised version of the manuscript.

Line 360. "lack of phenotype", this could be more specific.

Response: 'lack of phenotype' has been replaced by 'absence of a growth-inhibiting effect'.

Line 382. I'm not sure if it is known whether adult shoots increase cell division at warm temperature. Possibly change to 'juvenile shoots'.

Response: Done.

Line 455. "which would enable (but also require) to integrate" or "which would enable (or be required) to integrate".

Response: Changed as suggested.

The previous finding of Yang et al, that warm temps promote cell division could be given more prominence in the MS.

Response: Agreed. We have explained their findings in the revised version of the manuscript in more detail.

Dear Marcel,

Thank you again for sharing your work and the submission of your manuscript (EMBOJ-2021-111921) to The EMBO Journal. Please accept my sincere apologies for the unusually long peer-review period take for your study. Your revised manuscript was sent back to three reviewers for evaluation; we have now received a report from one of them, which I enclose below. Please note that while feedback from referees #1 and #2 is still pending at this stage I have, in light of referee #3's positive feedback and in order to expedite the manuscript's processing, decided to proceed towards publication of your work, pending no technically overriding concerns are presented by referee #1. I will share the comments from referee #1 as soon as we receive them.

Please pay attention to referee #1's comment about Figure 5C. In addition, there are some small editorial points I would like you to take care of. In this regard would you please:
- save the manuscript as a .docx file with no figures and no track changes,
- present refernces using et al. after the tenth author,
- rename the conflict of interest statement the "Disclosure and competing interests" statement",
- remove the author credit section from the manuscript,
- remove the callout to Figure EV6G as there are only 5 EV figures,
- use the coloured template for the author checklist,
- include legends with the EV figures in the format explained in our guide to authors, and
- add an Appendix 1 file containing a table of contents with page numbers.

We now require the publication of source data, particularly for electrophoretic gels and blots, with the aim of making primary data more accessible and transparent to the reader. Please provide me with a PDF file per figure that contains the original, uncropped and unprocessed scans gels used in the figures. The PDF files should be labeled with the appropriate figure/panel number, and should have molecular weight markers; further annotation could be useful but is not essential. The PDF files will be published online with the article as supplementary "Source Data" files. Source Data should also include Excel tables to accompany your graphs. We anticipate that their inclusion will make your work more discoverable and usable to scientists in the future.

We include a synopsis of the paper (see http://emboj.embopress.org/). Please provide me with a general summary statement and 3-5 bullet points that capture the key findings of the paper.

We also need a summary figure for the synopsis. The size should be 550 wide by [200-400] high (pixels). You can also use something from the figures if that is easier.

EMBO Press is an editorially independent publishing platform for the development of EMBO scientific publications.

Best wishes,

William

William Teale, PhD
Editor
The EMBO Journal
w.teale@embojournal.org

Use the link below to submit your revision:

https://emboj.msubmit.net/cgi-bin/main.plex

Referee #3:

I really enjoyed the opportunity to read this manuscript again. The authors have put in a lot of work to address the reviewers comments and in my view, this has resulted in a better paper. I agree with the author's argument that this work should be seen as providing a global mechanism for root thermomorphogenic responses. I stand by my belief that this work will be highly cited, as sets the stage for future, more in-depth studies into how temperature controls root meristem cell division. That's not to say this paper does not offer mechanistic insights; The extra data that the authors have provided has really helped to help strengthen these aspects of the study. All of my previous comments have been addressed.

I did notice a duplicate image in Figure 5C. Given the similarity of these images, this is an easy mistake to make, but I would urge the authors to thoroughly check through all other images to be sure that this is an isolated incident.

Response to the reviewer

Referee #3

I really enjoyed the opportunity to read this manuscript again. The authors have put in a lot of work to address the reviewers comments and in my view, this has resulted in a better paper. I agree with the author's argument that this work should be seen as providing a global mechanism for root thermomorphogenic responses. I stand by my belief that this work will be highly cited, as sets the stage for future, more in-depth studies into how temperature controls root meristem cell division. That's not to say this paper does not offer mechanistic insights; The extra data that the authors have provided has really helped to help strengthen these aspects of the study. All of my previous comments have been addressed. I did notice a duplicate image in Figure 5C. Given the similarity of these images, this is an easy mistake to make, but I would urge the authors to thoroughly check through all other images to be sure that this is an isolated incident.

Response: Thank you very much for the positive feedback. We especially appreciate spotting the figure assembly error, which naturally has immediately been corrected.

3rd Revision - Editorial Decision 21st Mar 2023

Dear Marcel,

I am pleased to inform you that your manuscript has been accepted for publication in the EMBO Journal.

Congratulations! This is a really exciting study - I'm really proud to have it in The EMBO Journal.

Please note that it is EMBO Journal policy for the transcript of the editorial process (containing referee reports and your response letter) to be published as an online supplement to each paper. If you do NOT want this, you will need to inform the Editorial Office via email immediately. More information is available here:
https://www.embopress.org/page/journal/14602075/authorguide#transparentprocess

Your manuscript will be processed for publication in the journal by EMBO Press. Manuscripts in the PDF and electronic editions of The EMBO Journal will be copy edited, and you will be provided with page proofs prior to publication. Please note that supplementary information is not included in the proofs.

You will be contacted by Wiley Author Services to complete licensing and payment information. The required 'Page Charges Authorization Form' is available here: https://www.embopress.org/pb-assets/embo-site/tej_apc.pdf - please download and complete the form and return to embopressproduction@wiley.com

EMBO Press participates in many Publish and Read agreements that allow authors to publish Open Access with reduced/no publication charges. Check your eligibility: https://authorservices.wiley.com/author-resources/Journal-Authors/open-access/affiliation-policies-payments/index.html

Should you be planning a Press Release on your article, please get in contact with embojournal@wiley.com as early as possible, in order to coordinate publication and release dates.

If you have any questions, please do not hesitate to call or email the Editorial Office. Thank you for your contribution to The EMBO Journal.

Best wishes,

William

William Teale, PhD
Editor
The EMBO Journal
w.teale@embojournal.org

** Click here to be directed to your login page: https://emboj.msubmit.net

EMBO Press Author Checklist

Corresponding Author Name: Marcel Quint	
Journal Submitted to: EMBO JOURNAL	
Manuscript Number: EMBOJ-2022-111926R1	

USEFUL LINKS FOR COMPLETING THIS FORM
The EMBO Journal - Author Guidelines
EMBO Reports - Author Guidelines
Molecular Systems Biology - Author Guidelines
EMBO Molecular Medicine - Author Guidelines

Reporting Checklist for Life Science Articles (updated January 2022)
This checklist is adapted from Materials Design Analysis Reporting (MDAR) Checklist for Authors. MDAR establishes a minimum set of requirements in transparent reporting in the life sciences (see Statement of Task: 10.31222/osf.io/9sm4x). Please follow the journal's guidelines in preparing your manuscript.
Please note that a copy of this checklist will be published alongside your article.

Abridged guidelines for figures
1. Data
The data shown in figures should satisfy the following conditions:
- the data were obtained and processed according to the field's best practice and are presented to reflect the results of the experiments in an accurate and unbiased manner.
- ideally, figure panels should include only measurements that are directly comparable to each other and obtained with the same assay.
- plots include clearly labeled error bars for independent experiments and sample sizes. Unless justified, error bars should not be shown for technical replicates.
- if n<5, the individual data points from each experiment should be plotted. Any statistical test employed should be justified.
- Source Data should be included to report the data underlying figures according to the guidelines set out in the authorship guidelines on Data Presentation.

2. Captions
Each figure caption should contain the following information, for each panel where they are relevant:
- a specification of the experimental system investigated (eg cell line, species name).
- the assay(s) and method(s) used to carry out the reported observations and measurements.
- an explicit mention of the biological and chemical entity(ies) that are being measured.
- an explicit mention of the biological and chemical entity(ies) that are altered/varied/perturbed in a controlled manner.
- the exact sample size (n) for each experimental group/condition, given as a number, not a range;
- a description of the sample collection allowing the reader to understand whether the samples represent technical or biological replicates (including how many animals, litters, cultures, etc.).
- a statement of how many times the experiment shown was independently replicated in the laboratory.
- definitions of statistical methods and measures:
  - common tests, such as t-test (please specify whether paired vs. unpaired), simple $\chi 2$ tests, Wilcoxon and Mann-Whitney tests, can be unambiguously identified by name only, but more complex techniques should be described in the methods section;
  - are tests one-sided or two-sided?
  - are there adjustments for multiple comparisons?
  - exact statistical test results, e.g., P values = x but not P values < x;
  - definition of 'center values' as median or average;
  - definition of error bars as s.d. or s.e.m.

Please complete ALL of the questions below.
Select "Not Applicable" only when the requested information is not relevant for your study.

Materials

Newly Created Materials	Information included in the manuscript?	In which section is the information available? (Reagents and Tools Table, Materials and Methods, Figures, Data Availability Section)
New materials and reagents need to be available; do any restrictions apply?	Not Applicable	

Antibodies	Information included in the manuscript?	In which section is the information available? (Reagents and Tools Table, Materials and Methods, Figures, Data Availability Section)
For **antibodies** provide the following information:<br>- Commercial antibodies: RRID (if possible) or supplier name, catalogue number and or/clone number<br>- Non-commercial: RRID or citation	Not Applicable	

DNA and RNA sequences	Information included in the manuscript?	In which section is the information available? (Reagents and Tools Table, Materials and Methods, Figures, Data Availability Section)
**Short novel DNA or RNA including primers, probes**: provide the sequences.	Yes	Materials and Methods

Cell materials	Information included in the manuscript?	In which section is the information available? (Reagents and Tools Table, Materials and Methods, Figures, Data Availability Section)
**Cell lines:** Provide species information, strain. Provide accession number in repository **OR** supplier name, catalog number, clone number, and/**OR** RRID.	Not Applicable	
**Primary cultures:** Provide species, strain, sex of origin, genetic modification status.	Not Applicable	
Report if the cell lines were recently **authenticated** (e.g., by STR profiling) and tested for mycoplasma contamination.	Not Applicable	

Experimental animals	Information included in the manuscript?	In which section is the information available? (Reagents and Tools Table, Materials and Methods, Figures, Data Availability Section)
**Laboratory animals or Model organisms:** Provide species, strain, sex, age, genetic modification status. Provide accession number in repository **OR** supplier name, catalog number, clone number, **OR** RRID.	Yes	Materials and Methods
**Animal observed in or captured from the field:** Provide species, sex, and age where possible.	Not Applicable	
Please detail **housing and husbandry conditions**.	Not Applicable	

Plants and microbes	Information included in the manuscript?	In which section is the information available? (Reagents and Tools Table, Materials and Methods, Figures, Data Availability Section)
**Plants:** provide species and strain, ecotype and cultivar where relevant, unique accession number if available, and source (including location for collected wild specimens).	Yes	Materials and Methods
**Microbes:** provide species and strain, unique accession number if available, and source.	Not Applicable	

Human research participants	Information included in the manuscript?	In which section is the information available? (Reagents and Tools Table, Materials and Methods, Figures, Data Availability Section)
If collected and within the bounds of privacy constraints report on age, sex and gender or ethnicity for all study participants.	Not Applicable	

Core facilities	Information included in the manuscript?	In which section is the information available? (Reagents and Tools Table, Materials and Methods, Figures, Data Availability Section)
If your work benefited from core facilities, was their service mentioned in the acknowledgments section?	Not Applicable	

Design

Study protocol	Information included in the manuscript?	In which section is the information available? (Reagents and Tools Table, Materials and Methods, Figures, Data Availability Section)
If study protocol has been **pre-registered, provide DOI in the manuscript**. For clinical trials, provide the trial registration number **OR** cite DOI.	Not Applicable	
Report the **clinical trial registration number** (at ClinicalTrials.gov or equivalent), where applicable.	Not Applicable	

Laboratory protocol	Information included in the manuscript?	In which section is the information available? (Reagents and Tools Table, Materials and Methods, Figures, Data Availability Section)
Provide DOI OR other citation details if **external detailed step-by-step protocols** are available.	Not Applicable	

Experimental study design and statistics	Information included in the manuscript?	In which section is the information available? (Reagents and Tools Table, Materials and Methods, Figures, Data Availability Section)
Include a statement about **sample size** estimate even if no statistical methods were used.	Yes	Figures
Were any steps taken to minimize the effects of subjective bias when allocating animals/samples to treatment (e.g. **randomization procedure**)? If yes, have they been described?	Not Applicable	
Include a statement about **blinding** even if no blinding was done.	Not Applicable	
Describe **inclusion/exclusion criteria** if samples or animals were excluded from the analysis. Were the criteria pre-established?<br><br>If sample or data points were omitted from analysis, report if this was due to attrition or intentional exclusion and provide justification.	Not Applicable	
For every figure, are **statistical tests** justified as appropriate? Do the data meet the assumptions of the tests (e.g., normal distribution)? Describe any methods used to assess it. Is there an estimate of variation within each group of data? Is the variance similar between the groups that are being statistically compared?	Yes	Figures

Sample definition and in-laboratory replication	Information included in the manuscript?	In which section is the information available? (Reagents and Tools Table, Materials and Methods, Figures, Data Availability Section)
In the figure legends: state number of times the experiment was **replicated** in laboratory.	Yes	Figures
In the figure legends: define whether data describe **technical or biological replicates**.	Yes	Figures

Ethics

Ethics	Information included in the manuscript?	In which section is the information available? (Reagents and Tools Table, Materials and Methods, Figures, Data Availability Section)
Studies involving **human participants**: State details of **authority granting ethics approval** (IRB or equivalent committee(s), provide reference number for approval.	Not Applicable	
Studies involving **human participants**: Include a statement confirming that **informed consent** was obtained from all subjects and that the experiments conformed to the principles set out in the WMA Declaration of Helsinki and the Department of Health and Human Services Belmont Report.	Not Applicable	
Studies involving **human participants:** For publication of **patient photos**, include a statement confirming that consent to publish was obtained.	Not Applicable	
Studies involving experimental **animals**: State details of **authority granting ethics approva**l (IRB or equivalent committee(s), provide reference number for approval. Include a statement of compliance with ethical regulations.	Not Applicable	
Studies involving **specimen and field samples:** State if relevant **permits** obtained, provide details of authority approving study; if none were required, explain why.	Not Applicable	

Dual Use Research of Concern (DURC)	Information included in the manuscript?	In which section is the information available? (Reagents and Tools Table, Materials and Methods, Figures, Data Availability Section)
Could your study fall under dual use research restrictions? Please check biosecurity documents and list of **select agents and toxins** (CDC): https://www.selectagents.gov/sat/list.htm	Not Applicable	
If you used a select agent, is the security level of the lab appropriate and reported in the manuscript?	Not Applicable	
If a study is subject to dual use research of concern regulations, is the name of the **authority granting approval and reference number** for the regulatory approval provided in the manuscript?	Not Applicable	

Reporting

The MDAR framework recommends adoption of discipline-specific guidelines, established and endorsed through community initiatives. Journals have their own policy about requiring specific guidelines and recommendations to complement MDAR.

Adherence to community standards	Information included in the manuscript?	In which section is the information available? (Reagents and Tools Table, Materials and Methods, Figures, Data Availability Section)
State if relevant guidelines or checklists (e.g., **ICMJE, MIBBI, ARRIVE, PRISMA**) have been followed or provided.	Not Applicable	
For **tumor marker prognostic studies**, we recommend that you follow the **REMARK** reporting guidelines (see link list at top right). See author guidelines, under 'Reporting Guidelines'. Please confirm you have followed these guidelines.	Not Applicable	
For **phase II and III randomized controlled trials**, please refer to the **CONSORT** flow diagram (see link list at top right) and submit the CONSORT checklist (see link list at top right) with your submission. See author guidelines, under 'Reporting Guidelines'. Please confirm you have submitted this list.	Not Applicable	

Data Availability

Data availability	Information included in the manuscript?	In which section is the information available? (Reagents and Tools Table, Materials and Methods, Figures, Data Availability Section)
Have **primary datasets** been deposited according to the journal's guidelines (see 'Data Deposition' section) and the respective accession numbers provided in the Data Availability Section?	Not Applicable	
Were **human clinical and genomic datasets** deposited in a public access-controlled repository in accordance to ethical obligations to the patients and to the applicable consent agreement?	Not Applicable	
Are **computational models** that are central and integral to a study available without restrictions in a machine-readable form? Were the relevant accession numbers or links provided?	Not Applicable	
If publicly available data were reused, provide the respective **data citations in the reference list**.	Not Applicable	
